# [3 + 2] Cycloaddition of thioformylium methylide with various arylidene-azolones in the synthesis of 7-thia-3-azaspiro[4.4]nonan-4-ones

**DOI:** 10.3762/bjoc.21.141

**Published:** 2025-09-05

**Authors:** Daniil I Rudik, Irina V Tiushina, Anatoly I Sokolov, Alexander Yu Smirnov, Alexander R Romanenko, Alexander A Korlyukov, Andrey A Mikhaylov, Mikhail S Baranov

**Affiliations:** 1 Institute of Bioorganic Chemistry, Russian Academy of Sciences, Miklukho-Maklaya 16/10, 117997, Moscow, Russia; 2 Pirogov Russian National Research Medical University, Ostrovitianov 1, 117997, Moscow, Russia; 3 Nesmeyanov Institute of Organoelement Compounds, Vavilova Street 28, 119334, Moscow, Russia

**Keywords:** arylidene-azolones, cycloaddition, nitrogen heterocycles, sulfur heterocycles, thioformylium methylide

## Abstract

Thioformylium methylide, which is readily generated from chloromethyl(trimethylsilyl)methyl sulfide by the action of fluoride, is used for the synthesis of spirocyclic derivatives from arylidene-azolones. Four types of the corresponding heterocycles have been studied. A series of 7-thia-3-azaspiro[4.4]nonan-4-ones was obtained with yields varying from 17 to 99%. The stereochemical study revealed selective formation of single either *cis* or *trans* stereoisomers, dependent on the heterocycle core used.

## Introduction

**Scheme 1:**
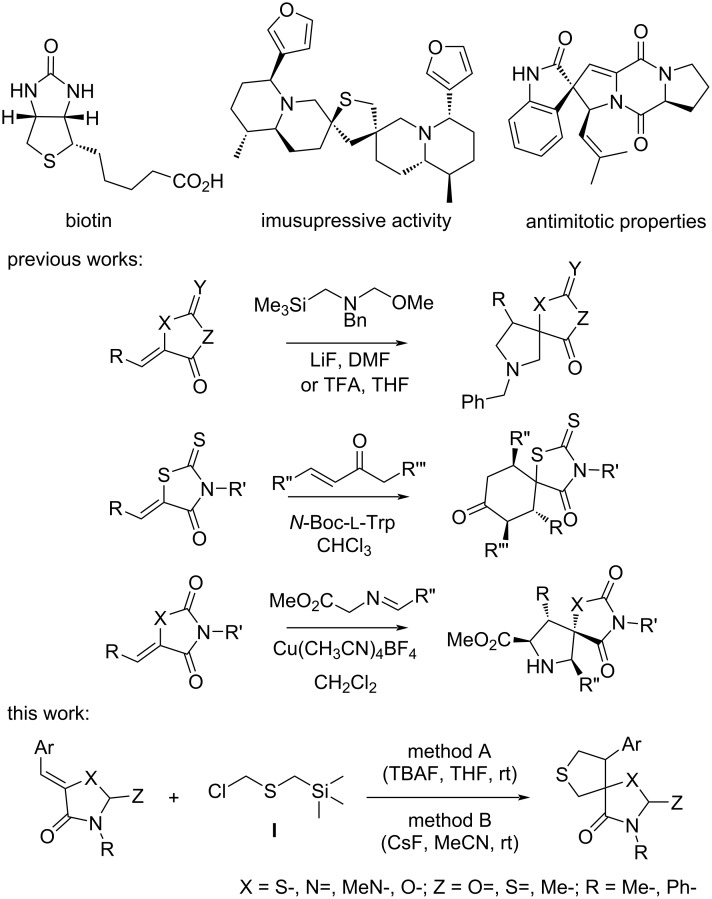
Synthetic and natural spirocyclic tetrahydrothiophene derivatives with pharmacological activities. Known and presented in this study is the cycloaddition reaction of various arylidene-azolones and thioformylium methylide.

Spirocyclic derivatives of heterocycles occupy an important place in modern organic and bioorganic chemistry [1]. Such substances are actively investigated in drug design, since their rigid 3D structure allows them to bind more selectively and effectively to biological targets in comparison to classical planar heterocycles [1–5]. Spirocyclic derivatives containing at least one five-membered heterocyclic ring are of particular interest, since they exhibit a wide range of pharmacological activity (Scheme 1) [6–11]. The most straightforward and powerful methods of such substance’s syntheses are based on cycloaddition reactions [12–15]. Previously, we and other authors have shown that arylidene-azolones are very promising substrates for these transformations, since their exo-cyclic double bond easily reacts with various 1,3-dipoles [16–20]. Thioformylium methylide is a well known 1,3-dipole, which is readily generated from chloromethyl(trimethylsilyl)methyl sulfide (compound **I**, Scheme 1) by the action of fluorides [21]. It reacts with a wide variety of compounds to form five-membered sulfur-containing heterocycles [22–24]. In particular, it can be used for the synthesis of 3,4-disubstituted tetrahydrothiophenes [25]. Partially substituted tetrahydrothiophenes are known to exhibit different biological activities [8]. However, to date the use of this reagent in the synthesis of spirocyclic derivatives to our knowledge is underinvestigated [26–27].

## Results and Discussion

In this work we present a systematic study of [3 + 2] cycloaddition of thioformylium methylide with various arylidene-azolones.

**Scheme 2:**
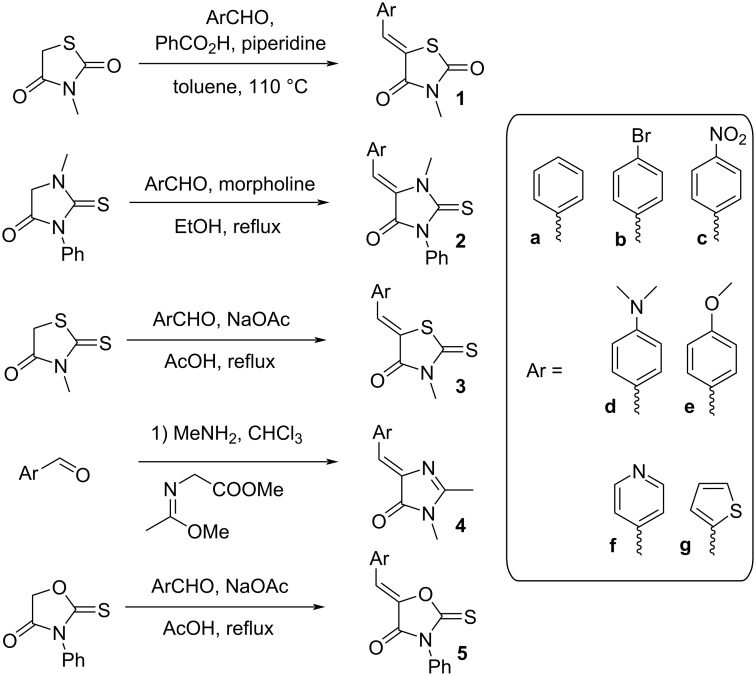
Synthesis of starting azolones **1**–**5**.

First, a series of various arylidene-azolones **1**–**5** were prepared (Scheme 2). All compounds were created in accordance with our previously published protocols [20,28–29].

Next, optimization of [3 + 2]-cycloaddition reaction condition was performed using derivatives **1–5e** with para-methoxy group in the aromatic ring (Table 1, Supporting Information File 1, part 2). Typically thioformylium methylide is generated from compound **I** by action of CsF or TBAF [16,23–24]. We also tested other fluorides and trifluoroacetic acid (Supporting Information File 1, part 2) and found that only these two abovementioned approaches are capable to perform desired cycloaddition (Supporting Information File 1, part 2). For various azolones **1**–**5e**, these two methods demonstrated different efficiency – Table 1. Derivative **6e** was formed from azolone **1e** in approximately equal yield provided by both methods (with cesium fluoride being preferred), while for rhodanine **3e** this method was able to produce almost quantitative yield of cycloadduct **8e**. In contrast, for hydantoin **2e** and imidazolone **4e** the use of TBAF was preferable. It is interesting to note that in all cases, we obtained only a single diastereomer with traces or no of the other one (dr >19:1). For the derivative **5e** with an oxygen-containing heterocycle, we failed to obtain the corresponding products under any of the proposed conditions. In all cases, we observed only very complex mixtures with traces of the target product.

**Table 1:** Optimization of the reaction conditions^a^.

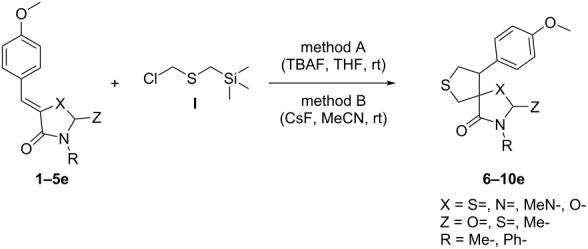			

Entry^a^	Azolone	Method A	Method B

1	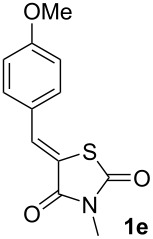	72% single isomer	**73%** **single isomer**
2	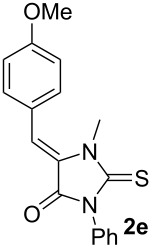	**98%** **single isomer**	84% single isomer
3	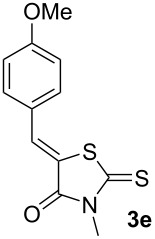	85% single isomer	**97%** **single isomer**
4	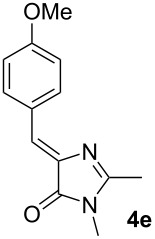	**99%** **single isomer**	58% single isomer
5	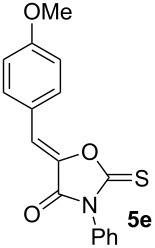	complex reaction mixture	complex reaction mixture

^a^Reaction conditions: 0.35 mmol of **1**–**5e** was dissolved in 3 mL of solvent, (((chloromethyl)thio)methyl)trimethylsilane and corresponding initiator were added (see Supporting Information File 1, part 2 for more details). Isolated yields are presented.

**Scheme 3:**
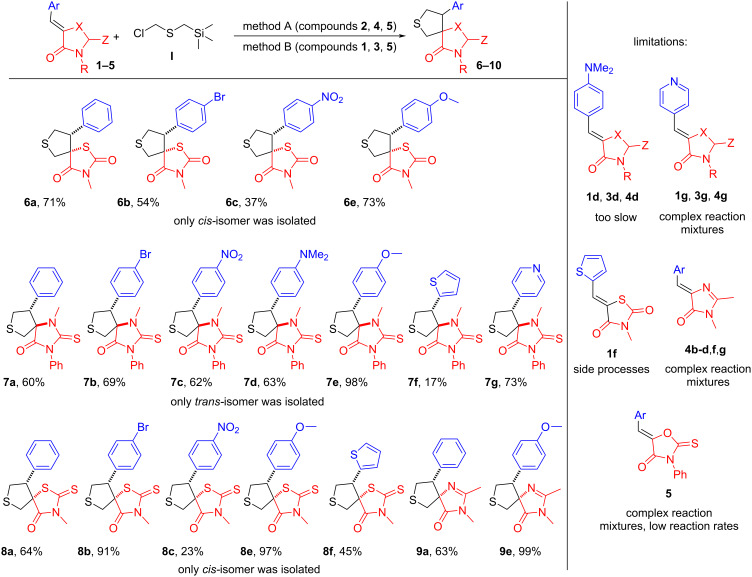
Reaction scope.

Next, using the revealed optimal reaction conditions, we studied its scope on a variety of arylidene-azolones **1**–**4** (Scheme 3). We also tried to apply both proposed approaches (methods A and B) to the series of derivatives **5**.

In contrast to the results obtained previously for *N*-benzylazomethine methylide [20]. generality and overall yields of the [2 + 3] cycloaddition of thioformylium methylide with azolones was not as effective. Possible alkylation of pyridine derivatives **1**,**3** and **4g** by initial compound **I** did not allow us to obtain the corresponding products. A similar problem was observed for imidazolone derivatives and **4b**–**d**,**f**,**g** for which we were also unable to obtain the corresponding spirocyclic products. Also, rate of the reaction for aniline derivatives **1**,**3** and **4d** was too slow, and therefore, we were able to obtain only the corresponding derivative of thiohydantoin **7d**. In case of thiophene derivatives the reaction was accompanied by a number of side-processes, which resulted in inseparable by-products, and thus we were unable to isolate the product of the reaction of the derivative **1f**. In addition, a decrease in yields was observed in the presence of a nitrophenyl group (compounds **6**–**8c**). Other compounds were obtained in moderate to good yields. Of particular note is the series of thiohydantoin derivatives **7**, for which all seven corresponding products were obtained. For all derivatives **5**, neither of the two proposed methods yielded the target products. In each case, only highly complex mixtures were formed, and the transformation rate was extremely low. This outcome may be attributed to the electron-acceptor properties of the oxygen atom, which likely disrupt the cycloaddition process.

**Figure 1:**
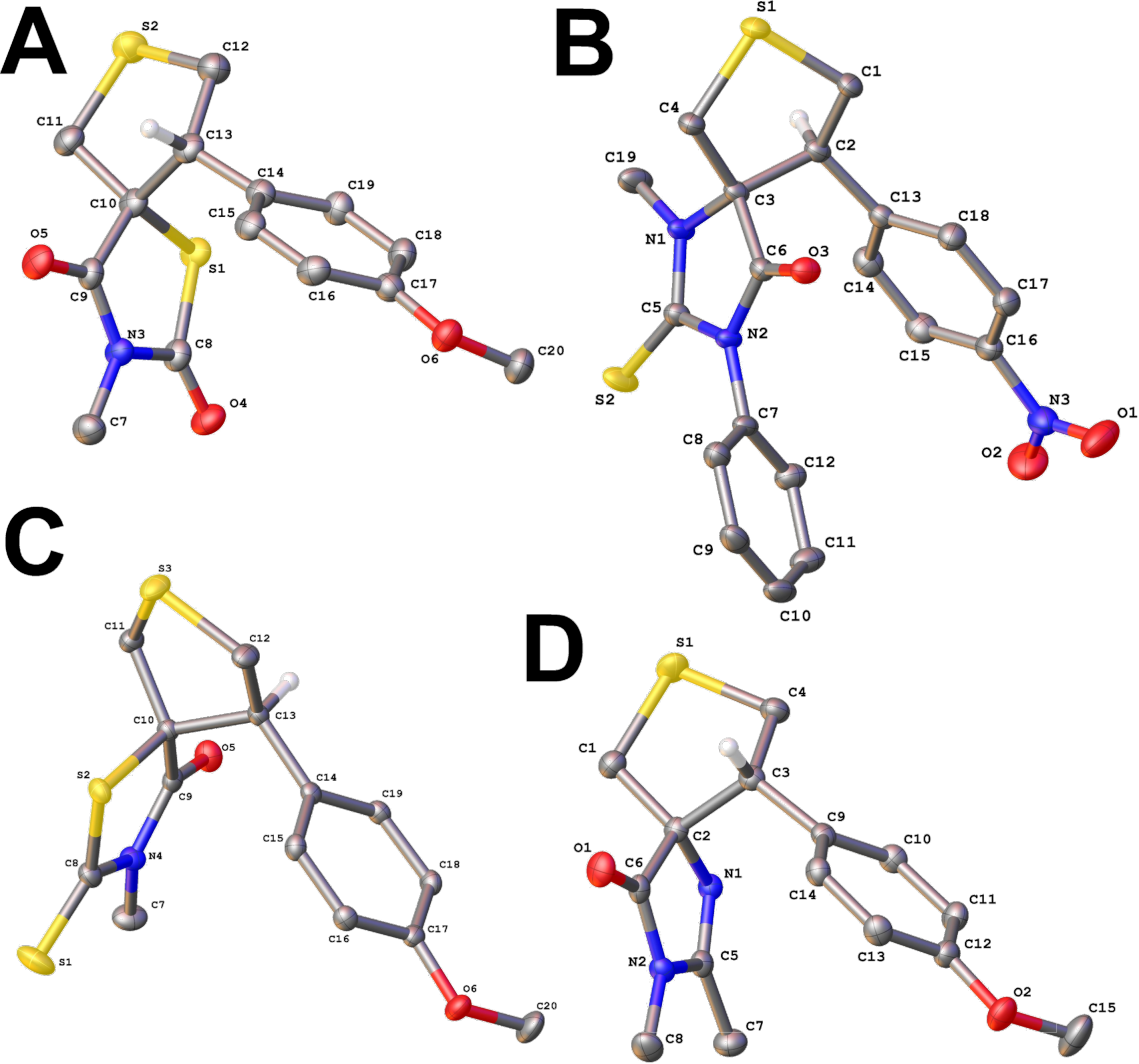
Single crystal X-ray analysis for the compounds **6e** (A), **7d** (B), **8e** (C) and **9e** (D). Atoms are shown as thermal ellipsoids at 50% probability. All hydrogen atoms, except the one at the stereogenic center, are omitted for clarity.

NMR analysis confirmed that each heterocyclic ring formed products exclusively as a single diastereomer, with only trace amounts (or none) of the alternative form (dr >19:1). Moreover, comparison of NMR data within each series of heterocycles allowed us to reliably assert that the same isomer was observed in each specific group of substances. However, NMR data alone could not unambiguously assign the relative configuration (*cis* or *trans*) of the substituents. Therefore, the structures of the obtained compounds were determined by single crystal X-ray analysis (Figure 1). Thus, all the obtained derivatives of oxo-rhodanine **6**, rhodanine **8** and imidazolone **9** had a *cis* configuration, while the derivatives of thiohydantoin **7**, on the contrary, have a *trans* configuration of two vicinal stereocenters (Scheme 3 and Figure 1).

Thus, in all cases we have obtained, probably, the most thermodynamically favorable isomers. This result allows us to assume a stepwise mechanism of the ylide addition to the double bond of thiohydantoin derivatives, since the initial compounds **2** had a predominant *Z*-configuration. At the same time, for cases **1**, **3** and **4**, both a synchronous and stepwise mechanism are possible. The discovered stereochemistry of the reaction correlates well with the previously obtained data, when the most thermodynamically stable *cis*- and *trans*- isomers were also obtained in the reaction with azomethine ylides [20].

**Scheme 4:**
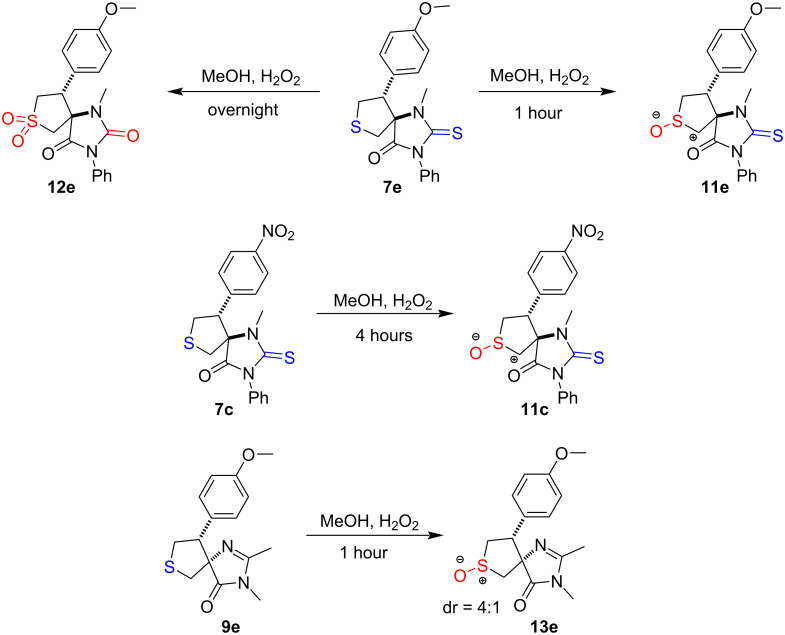
Oxidation of thioether group.

Finally, we performed preliminary derivatization studies of the obtained spiro-tetrahydrothiophenes. Compounds **7** and **9** demonstrated remarkable stability under both acidic and basic hydrolysis conditions (Supporting Information File 1, part 4). In contrast, compounds **8** and **6** quickly transformed into the single product (by TLC analysis) under the action of alkali, but this product was unstable in its individual form after evaporation from the solution (Supporting Information File 1, part 4). Oxidation, however, proved to be a more promising derivatization approach (Scheme 4). Thus, the short-term action of hydrogen peroxide successfully converted compounds **7e**, **7c**, and **9e** to their corresponding sulfoxides **11e**, **11c**, and **13e**. However, prolonged oxidation led not only to sulfone formation but also to deeper transformations. For example, compound **7e** underwent both sulfone formation and S/O atoms exchange in the heterocyclic core. For derivatives **6** and **8**, any oxidation conditions resulted in even more complex product mixtures (presumably due to the additional sulfur atom in the second ring), whose structures could not be determined.

## Conclusion

A study on [3 + 2]-cycloaddition reaction of various arylidene-azolones, with thioformylium methylide was performed. We have shown that cesium fluoride or TBAF can be an effective source of fluoride iones for activation of chloromethyl(trimethylsilyl)methyl sulfide. The reaction is shown to proceed with exclusive formation of a single diastereomer of the spirocyclic product, the exact configuration of which is dependent on the nature of heterocycle employed. Key stereo- and electronic factors for successful realization of the cycliaddition reaction were discovered, which would be of help in further drug design of spirocyclic scaffolds.

## Supporting Information

File 1 Experimental part, X-ray data and copies of NMR spectra.

## Data Availability

All data that supports the findings of this study is available in the published article and/or the supporting information of this article.
