# Supplementary material for: [3 + 2] Cycloaddition of thioformylium methylide with various arylidene-azolones in the synthesis of 7-thia-3-azaspiro[4.4]nonan-4-ones
Source: Beilstein J Org Chem. 2025 Sep 5;21:1791–8. doi: 10.3762/bjoc.21.141 (PMC12415905; doi:10.3762/bjoc.21.141)
Supplement: File 1 — Experimental part, X-ray data and copies of NMR spectra. [file Beilstein_J_Org_Chem-21-1791-s001.pdf]

BEILSTEIN JOURNAL OF ORGANIC CHEMISTRY

Supporting Information

for

[3 + 2] Cycloaddition of thioformylium methylide with various arylidene-azolones in the synthesis of 7-thia-3-azaspiro[4.4]nonan-4-ones

Daniil I. Rudik, Irina V. Tiushina, Anatoly I. Sokolov, Alexander Yu. Smirnov, Alexander R. Romanenko, Alexander A. Korlyukov, Andrey A. Mikhaylov and Mikhail S. Baranov

Beilstein J. Org. Chem. **2025,** *21,* 1791–1798. doi:10.3762/bjoc.21.141

Experimental part, X-ray data and copies of NMR spectra

License and Terms: This is a supporting information file under the terms of the Creative Commons Attribution License (https://creativecommons.org/licenses/by/4.0). Please note that the reuse, redistribution and reproduction in particular requires that the author(s) and source are credited and that individual graphics may be subject to special legal provisions.
The license is subject to the *Beilstein Journal of Organic Chemistry* terms and conditions: (https://www.beilstein-journals.org/bjoc/terms)

Contents

1. General S2
2. Optimization S3
   2.1. Step 1 .... S3
   2.2. Step 2 .... S6
3. Synthesis of spiro-products **6–9** S7
4. Derivatization study S18
   4.1. Hydrolysis of compounds **6a**, **7a**, **8c** and **9a** S18
   4.2. Oxidation of compounds **7c**, **7e** and **9e** S19
5. X-ray crystallography S22
6. References S24
7. Copies of NMR spectra S25
8. Copies of mass-spectra S48

1. General

NMR spectra were recorded on a 700 MHz Bruker Avance III NMR, 800 MHz Bruker Avance III 800 and Bruker Fourier 300 at 303°K. Chemical shifts are reported relative to residue peaks of $CDCl_3$ (7.27 ppm for ^{1}H and 77.2 ppm for ^{13}C) or DMSO-d_6 (2.50 ppm for ^{1}H and 39.5 ppm for ^{13}C). Melting points were measured on a SMP 30 apparatus without correction. High-resolution mass spectra (HRMS) spectra were recorded on AB Sciex TripleTOF® 5600+ System using electrospray ionization (ESI). The measurements were done in a positive ion mode (interface capillary voltage – 5500 V); mass range from *m*/*z* 50 to *m*/*z* 3000; external or internal calibration was done with ESI Tuning Mix, Agilent. A syringe injection was used for solutions in methanol +0.1% formic acid (flow rate 100 µL/min). Nitrogen was applied as a dry gas; interface temperature was set at 200 °C.

Commercially available reagents were used without additional purification. Solvents were distilled before use. E. Merck Kieselgel 60 was used for column chromatography. Thin layer chromatography (TLC) was performed on silica gel $60F_{254}$ glass-backed plates (MERCK). Visualization was performed using UV light (254 or 312 nm) or staining with ethanol solution of phosphomolybdic acid.

Arylidene-azolones **1–3, 5**[1] and arylidene-imidazolones **4**[2,3] was synthesized and described in previous works. (((Chloromethyl)thio)methyl)trimethylsilane was synthesized according to literature.[4]

2. Optimization

2.1. Step 1

The first optimization was carried out on azolone **3e** in order to determine the solvent and fluorine source suitable for the cycloaddition reaction.

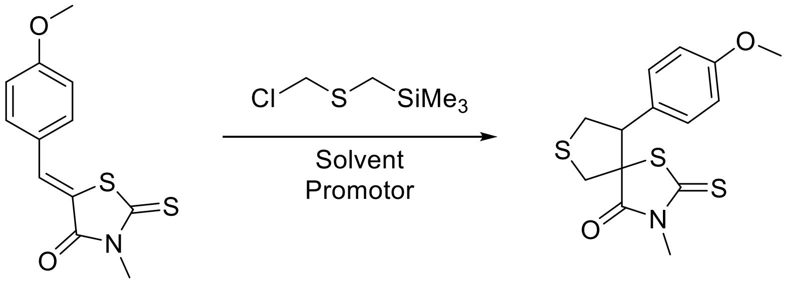

Table S1. First optimization.

№	Solvent	Promoter	T (°C)	Time (h)	Yield (%)
1	DMF	LiF	25	72	-
2	DMF	LiF	155	72	-
3	MeCN	LiF	25	72	-
4	MeCN	LiF	80	72	-
**5**	**THF**	**TBAF**	**25**	**72**	**85**
**6**	**MeCN**	**CsF**	**25**	**72**	**97**
7	THF	TFA	25	72	-

Cycloaddition procedure variant 1:

Arylideneazolone (0.35 mmol, 1 equiv) was dissolved in DMF (3 mL) then (((chloromethyl)thio)methyl)trimethylsilane (88 mg, 1.5 equiv) and LiF (18 mg, 2 equiv) was added to the solution. The reaction mixture was stirred for 72 h at room tempetature. The reaction progress was monitored with TLC. After 72 h aliquot (75 µL) was evaporated, dissolved in $CDCl_3$ and ^1H NMR spectrum was recorded. The obtained spectrum demonstrated that there is no desired product formed.

Cycloaddition procedure variant 2:

Arylideneazolone (0.35 mmol, 1 equiv was dissolved in DMF (3 mL) then (((chloromethyl)thio)methyl)trimethylsilane (88 mg, 1.5 equiv) and LiF (18 mg, 2 equiv) was added to the solution. The reaction mixture was stirred for 72 h on oil bath at 155 °C. The reaction progress was monitored with TLC. After 72 h aliquot (75 µL) was evaporated, dissolved in $CDCl_3$ and ^1H NMR spectrum was recorded. The obtained spectrum demonstrated that there is no desired product formed.

Cycloaddition procedure variant 3:

Arylideneazolone (0.35 mmol, 1 equiv) was dissolved in MeCN (3 mL) then (((chloromethyl)thio)methyl)trimethylsilane (88 mg, 1.5 equiv) and LiF (18 mg, 2 equiv) was added to the solution. The reaction mixture was stirred for 72 h at room temperature. The reaction progress was monitored with TLC. After 72 h aliquot (75 µL) was evaporated, dissolved in $CDCl_3$ and ^1H NMR spectrum was recorded. The obtained spectrum demonstrated that there is no desired product formed.

Cycloaddition procedure variant 4:

Arylideneazolone (0.35 mmol, 1 equiv) was dissolved in MeCN (3 mL) then (((chloromethyl)thio)methyl)trimethylsilane (88 mg, 1.5 equiv) and LiF (18 mg, 2 equiv) was added to the solution. The reaction mixture was stirred for 72 h on oil bath at 80 °C. The reaction progress was monitored with TLC. After 72 h aliquot (75 µL) was evaporated, dissolved in $CDCl_3$ and ^1H NMR spectrum was recorded. The obtained spectrum demonstrated that there is no desired product formed.

Cycloaddition procedure variant 5 (Method A):

Arylideneazolone (0.35 mmol, 1 equiv) was dissolved in THF (2 mL) then (((chloromethyl)thio)methyl)trimethylsilane (88 mg, 1.5 equiv) and TBAF·$3H_2O$ (144 mg, 1.3 equiv) was added to the solution. The reaction mixture was stirred at least 24 h. The reaction progress was monitored with TLC. In cases when reaction progress stopped after 24 hours a new portion of (((chloromethyl)thio)methyl)trimethylsilane (88 mg, 1.5 equiv) and TBAF·$3H_2O$ (144 mg, 1.3 equiv) was added and the reaction mixture was stirred for additional 12–24 h (this step should be repeated if the starting material was still observed at TLC).Once complete, the reaction mixture was diluted with EtOAc (50 mL) and transferred to a separatory funnel. The mixture was washed with saturated KCl solution (3 × 10 mL). The organic phase was dried over anhydrous Na_2SO_4 and concentrated in vacuo. The crude product was purified by flash column chromatography (eluent – EtOAc/Hex).

Cycloaddition procedure variant 6 (Method B):

Arylideneazolone (0.35 mmol, 1 equiv) was dissolved in MeCN (3 mL) then (((chloromethyl)thio)methyl)trimethylsilane (88 mg, 1.5 equiv) and CsF (107 mg, 2 equiv) was added to the solution. The reaction mixture was stirred at least 24 h. The reaction progress was monitored with TLC. In cases when reaction progress stopped after 24 hours a new portion of (((chloromethyl)thio)methyl)trimethylsilane (1.5 equiv) and CsF (107 mg, 2 equiv) was added and the reaction mixture was stirred for additional 12–24 h (this step should be repeated if the

starting material was still observed at TLC). Once complete, the reaction mixture was diluted with EtOAc (50 mL) and transferred to a separatory funnel. The mixture was washed with saturated K_2CO_3 solution (2 × 15 mL) and then with sat. KCl solution (3 × 15 mL). The organic phase was dried over anhydrous Na_2SO_4 and concentrated in vacuo. The crude product was purified by flash column chromatography (eluent – EtOAc/Hex).

Cycloaddition procedure variant 7:

Arylideneazolone (0.35 mmol, 1 equiv) was dissolved in 1% TFA in THF (3 mL, 0.17 equiv) then (((chloromethyl)thio)methyl)trimethylsilane (88 mg, 1.5 equiv) was added to the solution. The reaction mixture was stirred for 72 h at room temperature. The reaction progress was monitored with TLC. After 72 h aliquot (75 µL) was evaporated, dissolved in $CDCl_3$ and ^1H NMR spectrum was recorded. The obtained spectrum demonstrated that there is no desired product formed.

2.2. Step 2

The second optimization was performed for each series of azolones using the most successful methods A and B. For the second optimization, para-methoxy substituted azolones were also used.

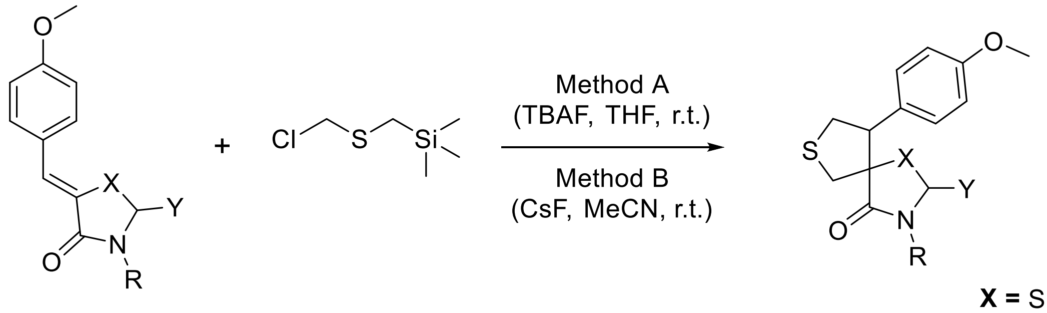

1-5e **6-10e**

X = S, N=, NMe, O
Y = O=, S=, NMe
R = Me, Ph

Table S2. Second optimization.

№	Azolone	Method A	Method B
1	1e	72% (Single isomer)	**73% (Single isomer)**
2	2e	**98% (Single isomer)**	84% (Single isomer)
3	3e	85% (Single isomer)	**97% (Single isomer)**
4	4e	**99% (Single isomer)**	58% (Single isomer)
5	5e	Complex reaction mixture	Complex reaction mixture

3. Synthesis of spiro-products 6–9

Method A

Arylideneazolone (0.35 mmol, 1 equiv) was dissolved in THF (2 mL) then (((chloromethyl)thio)methyl)trimethylsilane (88 mg, 1.5 equiv) and TBAF·3H_2O (144 mg, 1.3 equiv) was added to the solution. The reaction mixture was stirred at least 24 h. The reaction progress was monitored with TLC. In cases when reaction progress stopped after 24 hours a new portion of (((chloromethyl)thio)methyl)trimethylsilane (88 mg, 1.5 equiv) and TBAF·3H_2O (144 mg, 1.3 equiv) was added and the reaction mixture was stirred for additional 12–24 h (this step should be repeated if the starting material was still observed at TLC).Once complete, the reaction mixture was diluted with EtOAc (50 mL) and transferred to a separatory funnel. The mixture was washed with saturated KCl solution (3 × 10 mL). The organic phase was dried over anhydrous Na_2SO_4 and concentrated in vacuo. The crude product was purified by flash column chromatography (eluent – EtOAc/Hex).

Method B

Arylideneazolone (0.35 mmol, 1 equiv) was dissolved in MeCN (3 mL) then (((chloromethyl)thio)methyl)trimethylsilane (88 mg, 1.5 equiv) and CsF (107 mg, 2 equiv) was added to the solution. The reaction mixture was stirred at least 24 h. The reaction progress was monitored with TLC. In cases when reaction progress stopped after 24 hours a new portion of (((chloromethyl)thio)methyl)trimethylsilane (1.5 equiv) and CsF (107 mg, 2 equiv) was added and the reaction mixture was stirred for additional 12-24 h (this step should be repeated if the starting material was still observed at TLC). Once complete, the reaction mixture was diluted with EtOAc (50 mL) and transferred to a separatory funnel. The mixture was washed with saturated K_2CO_3 solution (2 × 15 mL) and then with sat. KCl solution (3 × 15 mL). The organic phase was dried over anhydrous Na_2SO_4 and concentrated in vacuo. The crude product was purified by flash column chromatography (eluent – EtOAc/Hex).

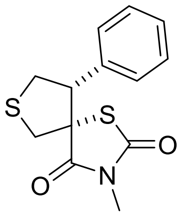

6a

***cis*-3-Methyl-9-phenyl-1,7-dithia-3-azaspiro[4.4]nonane-2,4-dione (6a)**

The compound was synthesized according to **Method B.** Three additional portions of (((chloromethyl)thio)methyl)trimethylsilane (1.5 equiv) and CsF (2 equiv) were added.

Yield: 70 mg (71%). White solid. M.p. 81-85 °C.

^{1}H NMR (800 MHz, $CDCl_3$) δ ppm 7.29 - 7.32 (m, 3 H), 7.24 - 7.26 (m, 2 H), 4.05 (dd, *J*=12.0, 7.4 Hz, 1 H), 3.94 (d, *J*=11.2 Hz, 1 H), 3.36 (t, *J*=11.5 Hz, 1 H), 3.26 - 3.30 (m, 2 H), 2.85 (s, 3 H).

^{13}C NMR (201 MHz, $CDCl_3$) δ ppm 174.2, 169.6, 133.7, 128.7, 128.6, 128.3, 72.0, 57.3, 42.2, 32.5, 27.6.

HRMS (ESI) m/z $[M+H]^+$ calcd for $C_{13}H_{14}NO_2S_2^+$: 280.0460, found: 280.0461.

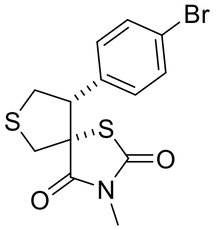

6b

***cis*-9-(4-Bromophenyl)-3-methyl-1,7-dithia-3-azaspiro[4.4]nonane-2,4-dione (6b)**

The compound was synthesized according to **Method B.** Three additional portions of (((chloromethyl)thio)methyl)trimethylsilane (1.5 equiv) and CsF (2 equiv) were added.

Yield: 68 mg (54%). White solid. M.p. 87-89 °C.

^{1}H NMR (800 MHz, $CDCl_3$) δ ppm 7.41 - 7.46 (m, 2 H), 7.11 - 7.15 (m, 2 H), 4.01 (dd, *J*=10.7, 8.5 Hz, 1 H), 3.92 (d, *J*=11.3 Hz, 1 H), 3.24 - 3.31 (m, 3 H), 2.90 (s, 3 H)

^{13}C NMR (201 MHz, $CDCl_3$) δ ppm 174.0, 169.3, 132.8, 131.6, 130.2, 122.9, 71.7, 56.5, 42.5, 32.6, 27.8.

HRMS (ESI) m/z $[M+H]^+$ calcd for $C_{13}H_{13}BrNO_2S_2^+$: 357.9566, found: 357.9566.

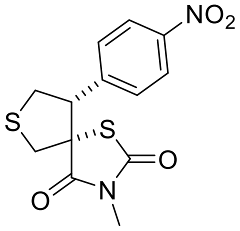

6c

***cis*-3-Methyl-9-(4-nitrophenyl)-1,7-dithia-3-azaspiro[4.4]nonane-2,4-dione (6c)**

The compound was synthesized according to **Method B.** Three additional portions of (((chloromethyl)thio)methyl)trimethylsilane (1.5 equiv) and CsF (2 equiv) were added.

Yield: 42 mg (37%). White solid. M.p. 203-204 °C.

^{1}H NMR (800 MHz, $CDCl_3$) δ ppm 8.18 (m, *J*=8.7 Hz, 2 H), 7.46 (m, *J*=8.6 Hz, 2 H), 4.17 (dd, *J*=11.4, 7.7 Hz, 1 H), 3.95 (d, *J*=11.3 Hz, 1 H), 3.29 - 3.38 (m, 3 H), 2.91 (s, 3 H)

^{13}C NMR (201 MHz, $CDCl_3$) δ ppm 173.7, 168.8, 148.1, 141.3, 129.7, 123.5, 71.6, 56.4, 42.9, 32.6, 28.0.

HRMS (ESI) m/z [M+H]+ calcd for $C_{13}H_{13}N_2O_4S_2^+$: 325.0311, found: 325.0309.

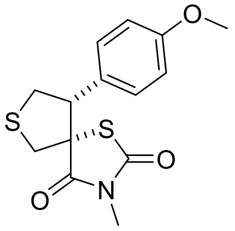

6e

***cis*-9-(4-Methoxyphenyl)-3-methyl-1,7-dithia-3-azaspiro[4.4]nonane-2,4-dione (6e)**

The compound was synthesized according to **Method B.** Two additional portions of (((chloromethyl)thio)methyl)trimethylsilane (1.5 equiv) and CsF (2 equiv) were added.

Yield: 79 mg (73%). slightly yellow solid. M.p. 144-146 °C.

^{1}H NMR (800 MHz, $CDCl_3$) δ ppm 7.18 (m, *J*=8.4 Hz, 2 H), 6.82 (m, *J*=8.5 Hz, 2 H), 4.01 (dd, *J*=11.9, 7.4 Hz, 1 H), 3.92 (d, *J*=11.3 Hz, 1 H), 3.79 (s, 3 H), 3.30 (t, *J*=11.4 Hz, 1 H), 3.22 - 3.27 (m, 2 H), 2.87 (s, 3 H).

^{13}C NMR (201 MHz, $CDCl_3$) δ ppm 174.3, 169.7, 159.7, 129.7, 125.5, 113.7, 72.1, 56.7, 55.2, 42.1, 32.7, 27.7.

HRMS (ESI) m/z [M+H]+ calcd for $C_{14}H_{16}NO_3S_2^+$: 310.0566, found: 310.0568.

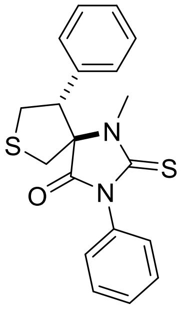

7a

***trans*-1-Methyl-3,9-diphenyl-2-thioxo-7-thia-1,3-diazaspiro[4.4]nonan-4-one (7a)**

The compound was synthesized according to **Method A.** Two additional portions of (((chloromethyl)thio)methyl)trimethylsilane (1.5 equiv) and TBAF (1.3 equiv) were added.

Yield: 74 mg (60%). Beige solid. M.p. 210-212 °C.

^{1}H NMR (800 MHz, $CDCl_3$) δ ppm 7.35 - 7.39 (m, 3 H), 7.30 - 7.34 (m, 3 H), 7.19 (d, *J*=7.3 Hz, 2 H), 6.58 (d, *J*=6.4 Hz, 2 H), 3.97 (t, *J*=11.7 Hz, 1 H), 3.89 (dd, *J*=12.6, 5.8 Hz, 1 H), 3.54 (s, 3 H), 3.52 (d, *J*=12.5 Hz, 1 H), 3.30 (d, *J*=12.6 Hz, 1 H), 3.10 (dd, *J*=11.0, 5.6 Hz, 1 H)

^{13}C NMR (201 MHz, $CDCl_3$) δ ppm 181.7, 173.3, 134.1, 132.6, 129.0, 128.9, 128.9, 128.6, 128.0, 127.9, 77.0, 55.4, 33.5, 32.0, 30.0..

HRMS (ESI) m/z $[M+H]^+$ calcd for $C_{19}H_{19}N_2OS_2^+$: 355.0933, found: 355.0932.

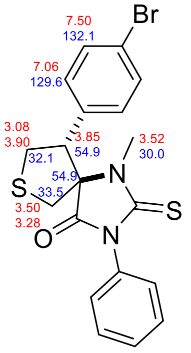

7b

***trans*-9-(4-Bromophenyl)-1-methyl-3-phenyl-2-thioxo-7-thia-1,3-diazaspiro[4.4]nonan-4-one (7b)**

The compound was synthesized according to **Method A.** Two additional portions of (((chloromethyl)thio)methyl)trimethylsilane (1.5 equiv) and TBAF (1.3 equiv) were added.

Yield: 105 mg (69%). White solid. M.p. 189-190 °C decomp.

^{1}H NMR (800 MHz, $CDCl_3$) δ ppm 7.50 (d, *J*=7.0 Hz, 2 H), 7.37 (d, *J*=4.1 Hz, 3 H), 7.06 (d, *J*=7.3 Hz, 2 H), 6.61 (br. s., 2 H), 3.90 (t, *J*=11.7 Hz, 1 H), 3.83 - 3.87 (m, 1 H), 3.49 - 3.55 (m, 4 H), 3.28 (d, *J*=12.6 Hz, 1 H), 3.08 (dd, *J*=10.8, 5.5 Hz, 1 H)

^{13}C NMR (201 MHz, $CDCl_3$) δ ppm 181.7, 173.2, 133.2, 132.5, 132.1, 129.6, 129.2, 129.0, 127.9, 122.8, 76.9, 54.9, 33.5, 32.1, 30.0.

HRMS (ESI) m/z $[M+H]^+$ calcd for $C_{19}H_{18}BrN_2OS_2^+$: 433.0038, found: 433.0037.

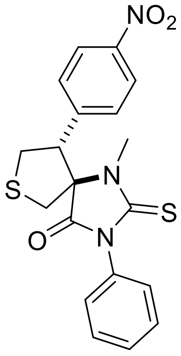

7c

***trans*-1-Methyl-9-(4-nitrophenyl)-3-phenyl-2-thioxo-7-thia-1,3-diazaspiro[4.4]nonan-4-one (7c)**

The compound was synthesized according to **Method A.** Two additional portions of (((chloromethyl)thio)methyl)trimethylsilane (1.5 equiv) and TBAF (1.3 equiv) were added.

Yield: 87 mg (62%). White solid. M.p. >250 °C decomp.

^{1}H NMR (800 MHz, $CDCl_3$) δ ppm 8.22 (m, *J*=8.7 Hz, 2 H), 7.38 (m, *J*=8.6 Hz, 2 H), 7.32 - 7.36 (m, 3 H), 6.61 (d, *J*=7.1 Hz, 2 H), 3.95 - 4.01 (m, 2 H), 3.56 (s, 3 H), 3.56 (d, J=12.6 Hz, 2 H), 3.33 (d, *J*=12.6 Hz, 1 H), 3.14 (dd, *J*=9.4, 4.0 Hz, 1 H)

^{13}C NMR (201 MHz, $CDCl_3$) δ ppm 181.5, 172.9, 148.0, 141.9, 132.2, 129.3, 129.1, 129.0, 127.6, 123.9, 55.0, 34.0, 32.3, 30.0.

HRMS (ESI) m/z $[M+H]^+$ calcd for $C_{19}H_{18}N_3O_3S_2^+$: 400.0784, found: 400.0782.

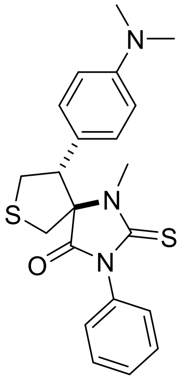

7d

***trans*-9-(4-(Dimethylamino)phenyl)-1-methyl-3-phenyl-2-thioxo-7-thia-1,3-diazaspiro[4.4]nonan-4-one (7d)**

The compound was synthesized according to **Method A.** Five additional portions of (((chloromethyl)thio)methyl)trimethylsilane (1.5 equiv) and TBAF (1.3 equiv) were added.

Yield: 88 mg (63%). White solid. M.p. 202-203 °C.

^{1}H NMR (800 MHz, $CDCl_3$) δ ppm 7.31 - 7.35 (m, 3 H), 7.04 (d, *J*=8.3 Hz, 2 H), 6.67 (d, *J*=8.2 Hz, 4 H), 3.91 (t, *J*=11.7 Hz, 1 H), 3.81 (dd, *J*=12.5, 5.8 Hz, 1 H), 3.51 (s, 3 H), 3.48 (d, *J*=12.5 Hz, 1 H), 3.27 (d, *J*=12.5 Hz, 1 H), 3.05 (dd, *J*=11.0, 5.8 Hz, 1 H), 2.95 (s, 6 H)

^{13}C NMR (201 MHz, $CDCl_3$) δ ppm 181.8, 173.7, 150.8, 132.9, 129.0, 128.8, 128.6, 128.3, 120.9, 112.6, 77.1, 55.0, 40.5, 33.2, 32.2, 30.0.

HRMS (ESI) m/z $[M+H]^+$ calcd for $C_{21}H_{24}N_3OS_2^+$: 398.1355, found: 398.1353.

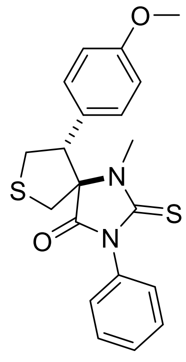

7e

***trans*-9-(4-Methoxyphenyl)-1-methyl-3-phenyl-2-thioxo-7-thia-1,3-diazaspiro[4.4]nonan-4-one (7e)**

The compound was synthesized according to **Method A.** One additional portion of (((chloromethyl)thio)methyl)trimethylsilane (1.5 equiv) and TBAF (1.3 equiv) was added.

Yield: 132 mg (98%). Beige solid. M.p. 190-191 °C decomp.

^{1}H NMR (800 MHz, $CDCl_3$) δ ppm 7.34 (d, *J*=3.4 Hz, 3 H), 7.11 (m, *J*=8.4 Hz, 2 H), 6.88 (m, *J*=8.4 Hz, 2 H), 6.66 (d, *J*=3.4 Hz, 2 H), 3.92 (t, *J*=11.7 Hz, 1 H), 3.85 (dd, *J*=12.4, 5.7 Hz, 1 H), 3.81 (s, 3 H), 3.52 (s, 3 H), 3.49 (d, *J*=12.5 Hz, 1 H), 3.28 (d, *J*=12.5 Hz, 1 H), 3.07 (dd, *J*=11.0, 5.8 Hz, 1 H)

^{13}C NMR (201 MHz, $CDCl_3$) δ ppm 181.7, 173.4, 159.9, 132.7, 129.1, 129.0, 128.9, 128.1, 125.8, 114.3, 77.0, 55.4, 54.7, 33.3, 32.2, 30.0.

HRMS (ESI) m/z $[M+H]^+$ calcd for $C_{20}H_{21}N_2O_2S_2^+$: 385.1039, found: 385.1040.

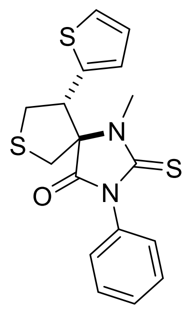

7f

***trans*-1-Methyl-3-phenyl-9-(thiophen-2-yl)-2-thioxo-7-thia-1,3-diazaspiro[4.4]nonan-4-one (7f)**

The compound was synthesized according to **Method A.** Five additional portions of (((chloromethyl)thio)methyl)trimethylsilane (1.5 equiv) and TBAF (1.3 equiv) were added.

Yield: 22 mg (17%). Beige solid. M.p. 145-146 °C.

^{1}H NMR (800 MHz, $CDCl_3$) δ ppm 7.35 - 7.38 (m, 3 H), 7.31 (d, *J*=5.0 Hz, 1 H), 7.04 (t, *J*=4.3 Hz, 1 H), 6.95 (d, *J*=3.1 Hz, 1 H), 6.75 (d, *J*=3.1 Hz, 2 H), 4.13 (dd, *J*=12.2, 6.0 Hz, 1 H), 3.94 (t, *J*=11.6 Hz, 1 H), 3.51 (s, 3 H), 3.47 (d, *J*=12.3 Hz, 1 H), 3.33 (d, *J*=12.3 Hz, 1 H), 3.23 (dd, *J*=10.9, 6.1 Hz, 1 H)

^{13}C NMR (201 MHz, $CDCl_3$) δ ppm 182.3, 172.9, 135.9, 132.7, 129.1, 129.0, 128.1, 127.3, 125.9, 125.5, 76.6, 49.8, 32.8, 32.6, 29.7.

HRMS (ESI) m/z $[M+H]^+$ calcd for $C_{17}H_{17}N_2OS_3^+$: 361.0498, found: 361.0501.

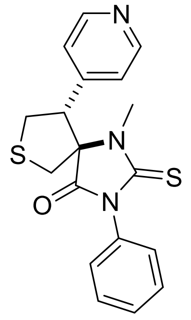

7g

***trans*-1-Methyl-3-phenyl-9-(pyridin-4-yl)-2-thioxo-7-thia-1,3-diazaspiro[4.4]nonan-4-one (7g)**

The compound was synthesized according to **Method A.** Three additional portions of (((chloromethyl)thio)methyl)trimethylsilane (1.5 equiv) and TBAF (1.3 equiv) were added.

Yield: 91 mg (73%). White solid. M.p. 187-188 °C decomp.

^{1}H NMR (800 MHz, $CDCl_3$) δ ppm 8.61 (d, *J*=5.7 Hz, 2 H), 7.33 - 7.37 (m, 3 H), 7.11 (d, *J*=5.8 Hz, 2 H), 6.59 (d, *J*=5.6 Hz, 2 H), 3.95 (t, *J*=11.8 Hz, 1 H), 3.86 (dd, *J*=12.4, 5.8 Hz, 1 H), 3.52 - 3.55 (m, 4 H), 3.31 (d, *J*=12.6 Hz, 1 H), 3.11 (dd, *J*=11.1, 5.8 Hz, 1 H)

^{13}C NMR (201 MHz, $CDCl_3$) δ ppm 181.7, 172.9, 150.4, 143.5, 132.3, 129.3, 129.1, 127.8, 122.9, 76.7, 54.4, 33.9, 31.7, 30.0.

HRMS (ESI) m/z $[M+H]^+$ calcd for $C_{18}H_{18}N_3OS_2^+$: 356.0886, found: 356.0888.

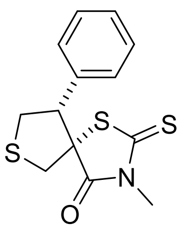

8a

***cis*-3-Methyl-9-phenyl-2-thioxo-1,7-dithia-3-azaspiro[4.4]nonan-4-one (8a)**

The compound was synthesized according to **Method B.** Three additional portions of (((chloromethyl)thio)methyl)trimethylsilane (1.5 equiv) and CsF (2 equiv) were added.

Yield: 66 mg (64%). White solid. M.p. 104-105 °C.

^1H NMR (800 MHz, $CDCl_3$) δ ppm 7.28 - 7.32 (m, 3 H), 7.24 - 7.26 (m, 2 H), 4.03 (dd, *J*=11.8, 7.4 Hz, 1 H), 3.90 (d, *J*=11.3 Hz, 1 H), 3.37 (t, *J*=11.4 Hz, 1 H), 3.27 - 3.32 (m, 2 H), 3.11 (s, 3 H)

^{13}C NMR (201 MHz, $CDCl_3$) δ ppm 198.5, 175.5, 133.5, 128.8, 128.4, 73.0, 57.8, 41.4, 32.7, 31.0.

HRMS (ESI) m/z $[M+H]^+$ calcd for $C_{13}H_{14}NOS_3^+$: 296.0232, found: 296.0237.

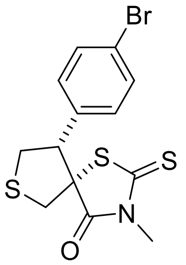

8b

***cis*-9-(4-Bromophenyl)-3-methyl-2-thioxo-1,7-dithia-3-azaspiro[4.4]nonan-4-one (8b)**

The compound was synthesized according to **Method B.** Three additional portions of (((chloromethyl)thio)methyl)trimethylsilane (1.5 equiv) and CsF (2 equiv) were added.

Yield: 119 mg (91%). Beige solid. M.p. 110-112 °C.

^1H NMR (800 MHz, $CDCl_3$) δ ppm 7.42 (m, *J*=8.4 Hz, 2 H), 7.14 (m, *J*=8.4 Hz, 2 H), 3.97 - 4.02 (m, 1 H), 3.88 (d, *J*=11.3 Hz, 1 H), 3.26 - 3.30 (m, 3 H), 3.16 (s, 3 H)

^{13}C NMR (201 MHz, $CDCl_3$) δ ppm 198.0, 175.4, 132.7, 131.6, 130.1, 123.0, 72.6, 56.9, 41.8, 32.8, 31.2.

HRMS (ESI) m/z $[M+H]^+$ calcd for $C_{13}H_{13}BrNOS_3^+$: 373.9337, found: 373.9337.

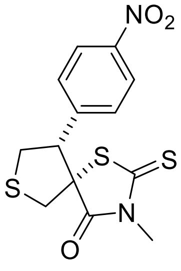

8c

***cis*-3-Methyl-9-(4-nitrophenyl)-2-thioxo-1,7-dithia-3-azaspiro[4.4]nonan-4-one (8c)**

The compound was synthesized according to **Method B.** Three additional portions of (((chloromethyl)thio)methyl)trimethylsilane (1.5 equiv) and CsF (2 equiv) were added.

Yield: 27 mg (23%). Slightly yellow solid. M.p. 176-177 °C.

^{1}H NMR (800 MHz, $CDCl_3$) δ ppm 8.16 (m, *J*=8.6 Hz, 2 H), 7.46 (m, *J*=8.6 Hz, 2 H), 4.15 (dd, *J*=11.2, 8.0 Hz, 1 H), 3.92 (d, *J*=11.4 Hz, 1 H), 3.34 - 3.38 (m, 2 H), 3.32 (d, *J*=11.4 Hz, 1 H), 3.16 (s, 3 H)

^{13}C NMR (201 MHz, $CDCl_3$) δ ppm 197.3, 175.1, 148.1, 141.1, 129.6, 123.5, 72.5, 56.8, 42.1, 32.7, 31.3.

HRMS (ESI) m/z $[M+H]^+$ calcd for $C_{13}H_{13}N_2O_3S_3^+$: 341.0083, found: 341.0082.

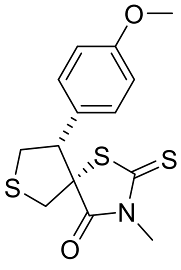

8e

***cis*-9-(4-Methoxyphenyl)-3-methyl-2-thioxo-1,7-dithia-3-azaspiro[4.4]nonan-4-one (8e)**

The compound was synthesized according to **Method B.** Three additional portions of (((chloromethyl)thio)methyl)trimethylsilane (1.5 equiv) and CsF (2 equiv) were added.

Yield: 110 mg (97%). White solid. M.p. 96-98 °C.

^{1}H NMR (800 MHz, $CDCl_3$) δ ppm 7.17 (m, *J*=8.6 Hz, 2 H), 6.80 (m, *J*=8.7 Hz, 2 H), 4.00 (dd, *J*=11.7, 7.5 Hz, 1 H), 3.87 (d, *J*=11.3 Hz, 1 H), 3.78 (s, 3 H), 3.30 (t, *J*=11.4 Hz, 1 H), 3.24 - 3.28 (m, 2 H), 3.13 (s, 3 H)

^{13}C NMR (201 MHz, $CDCl_3$) δ ppm 198.7, 175.6, 159.8, 129.5, 125.3, 113.7, 73.1, 57.2, 55.2, 41.3, 32.9, 31.1.

HRMS (ESI) m/z $[M+H]^+$ calcd for $C_{14}H_{16}NO_2S_3^+$: 326.0338, found: 326.0343.

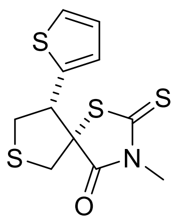

8f

***cis*-3-Methyl-9-(thiophen-2-yl)-2-thioxo-1,7-dithia-3-azaspiro[4.4]nonan-4-one (8f)**

The compound was synthesized according to **Method B.** Three additional portions of (((chloromethyl)thio)methyl)trimethylsilane (1.5 equiv) and CsF (2 equiv) were added.

Yield: 47 mg (45%). White solid. M.p. 93-94 °C.

^{1}H NMR (800 MHz, $CDCl_3$) δ ppm 7.22 - 7.25 (m, 1 H), 6.92 - 6.95 (m, 2 H), 4.34 (dd, *J*=11.8, 7.2 Hz, 1 H), 3.86 (d, *J*=11.3 Hz, 1 H), 3.40 (dd, *J*=11.0, 7.3 Hz, 1 H), 3.29 (d, *J*=11.3 Hz, 1 H), 3.21 - 3.25 (m, 4 H)

^{13}C NMR (201 MHz, $CDCl_3$) δ ppm 198.7, 175.4, 136.2, 126.8, 126.7, 125.8, 72.6, 53.5, 41.2, 34.5, 31.3.

HRMS (ESI) m/z $[M+H]^+$ calcd for $C_{11}H_{12}NOS_4^+$: 301.9796, found: 301.9799.

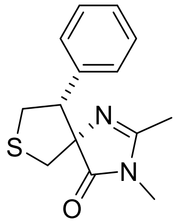

9a

***cis*-2,3-Dimethyl-9-phenyl-7-thia-1,3-diazaspiro[4.4]non-1-en-4-one (9a)**

Yield: 57 mg (63%). White solid. M.p. 112-113 °C.

The compound was synthesized according to **Method A.** One additional portion of (((chloromethyl)thio)methyl)trimethylsilane (1.5 equiv) and TBAF (1.3 equiv) was added.

^{1}H NMR (800 MHz, $CDCl_3$) δ ppm 3.70 - 3.74 (m, 1 H), 3.66 (dd, *J*=12.3, 10.1 Hz, 1 H), 3.50 (d, *J*=11.0 Hz, 1 H), 3.24 (dd, *J*=10.0, 6.5 Hz, 1 H), 2.90 (d, *J*=10.9 Hz, 1 H), 2.72 (s, 3 H), 2.00 (s, 3 H)

^{13}C NMR (201 MHz, $CDCl_3$) δ ppm 181.5, 160.8, 134.9, 128.6, 127.6, 127.7, 80.7, 58.5, 38.6, 34.8, 26.4, 15.1.

HRMS (ESI) m/z $[M+H]^+$ calcd for $C_{14}H_{17}N_2OS^+$: 261.1056, found: 261.1057.

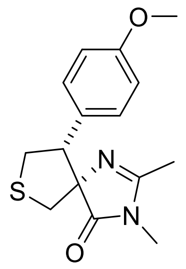

9e

***cis*-9-(4-Methoxyphenyl)-2,3-dimethyl-7-thia-1,3-diazaspiro[4.4]non-1-en-4-one (9e)**

The compound was synthesized according to **Method A.** One additional portion of (((chloromethyl)thio)methyl)trimethylsilane (1.5 equiv) and TBAF (1.3 equiv) was added.

Yield: 100 mg (99%). Slightly yellow solid. M.p. 135-137 °C.

^{1}H NMR (800 MHz, $CDCl_3$) δ ppm 7.12 (m, *J*=8.8 Hz, 2 H), 6.75 (m, *J*=8.8 Hz, 2 H), 3.77 (s, 3 H), 3.68 (dd, *J*=12.4, 6.7 Hz, 1 H), 3.60 (dd, *J*=12.3, 10.3 Hz, 1 H), 3.48 (d, *J*=11.0 Hz, 1 H), 3.20 (dd, *J*=10.2, 6.7 Hz, 1 H), 2.88 (d, *J*=11.0 Hz, 1 H), 2.74 (s, 3 H), 2.03 (s, 3 H)

^{13}C NMR (201 MHz, $CDCl_3$) δ ppm 181.6, 160.8, 159.0, 129.6, 126.9, 113.0, 80.6, 57.8, 55.1, 38.5, 35.0, 26.4, 15.1.

HRMS (ESI) m/z $[M+H]^+$ calcd for $C_{15}H_{19}N_2O_2S^+$: 291.1162, found: 291.1165.

4. Derivatization study

4.1. Hydrolysis of compounds 6a, 7a, 8c and 9a

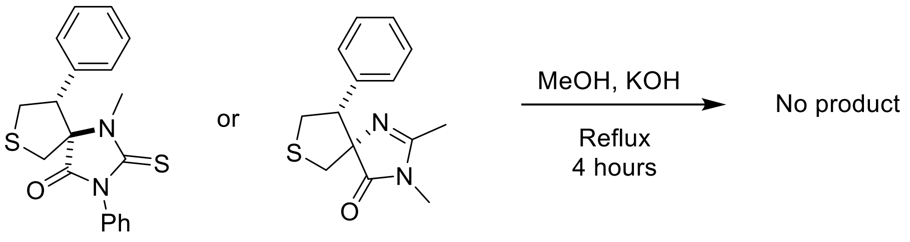

7a **9a**

Corresponding spiro-compound (0.1 mmol, 1 equiv) was dissolved in MeOH (2 mL) and KOH (20 equiv) was added to the solution. The reaction mixture was refluxed for 4 hours. The reaction progress was monitored with TLC.

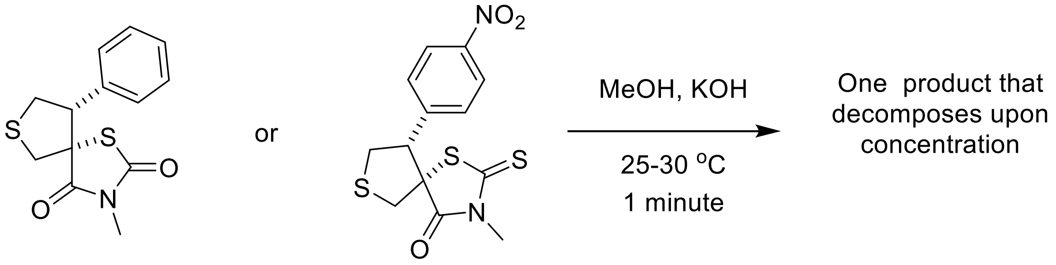

6a **8c**

Corresponding spiro-compound (0.1 mmol, 1 equiv) was dissolved in MeOH (2 mL) and KOH (20 equiv) was added to the solution. The reaction mixture was stirred at room temperature for 1 minute. The reaction mixture was diluted with EtOAc (20 mL) and transferred to a separatory funnel. The mixture was washed with water, saturated and KCl solution (3 × 20 mL). The organic phase was dried over anhydrous Na_2SO_4 and concentrated in vacuo. TLC showed a spot, which was no longer detectable after solvent removal under reduced pressure.

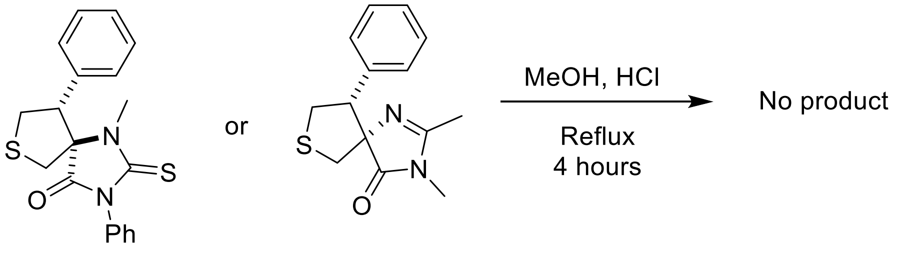

7a **9a**

Corresponding spiro-compound (0.1 mmol, 1 equiv) was dissolved in MeOH (1 mL) and concentrated HCl (1 mL) was added to the solution. The reaction mixture was refluxed for 4 hours. The reaction progress was monitored with TLC.

4.2. Oxidation of compounds 7c, 7e and 9e

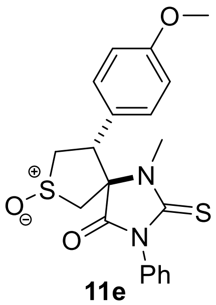

11e

***trans*-9-(4-Methoxyphenyl)-1-methyl-3-phenyl-2-thioxo-7-thia-1,3-diazaspiro[4.4]nonan-4-one 7-oxide (11e)**

Compound **7e** (0.1 mmol, 1 equiv) was dissolved in MeOH (2 mL) and H_2O_2 30% (40 equiv, 0.41 mL) was added to the solution. The reaction mixture was stirred at room temperature for 1 hour. The reaction mixture was diluted with $CHCl_3$ (20 mL) and transferred to a separatory funnel. The mixture was washed with water (1 × 10 mL), saturated and KCl solution (3 × 20 mL). The organic phase was dried over anhydrous Na_2SO_4 and concentrated in vacuo. The crude product was purified by flash column chromatography (eluent – EtOAc/Hex, v/v 3:1).

Yield: 32 mg (80%). White solid. M.p. 239-241 °C.

^{1}H NMR (300 MHz, $CDCl_3$) δ ppm 7.29 - 7.36 (m, 3 H), 7.12 (m, *J*=8.6 Hz, 2 H), 6.93 (m, *J*=8.6 Hz, 2 H), 6.47 – 6,55 (m, *J*=5.3, 2.5 Hz, 2 H), 4.85 (dd, *J*=14.4, 4.9 Hz, 1 H), 3.83 (s, 3 H), 3.71 (d, *J*=15.5 Hz, 1 H), 3.63 (s, 3 H), 3.56 (t, *J*=13.8 Hz, 1 H), 3.31 - 3.41 (m, 1 H), 3.21 (dd, *J*=15.4, 3.4 Hz, 1 H)

^{13}C NMR (75 MHz, $CDCl_3$) δ ppm 181.7, 173.4, 160.1, 132.4, 129.4, 129.2, 128.9, 127.9, 123.8, 114.5, 76.2, 57.1, 55.4, 52.9, 48.7, 31.1.

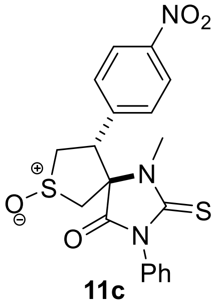

11c

***trans*-1-Methyl-9-(4-nitrophenyl)-3-phenyl-2-thioxo-7-thia-1,3-diazaspiro[4.4]nonan-4-one 7-oxide (11c)**

Compound 7c (0.1 mmol, 1 equiv) was dissolved in MeOH (2 mL) and H_2O_2 30% (0.41 mL, 40 equiv) was added to the solution. The reaction mixture was stirred at room temperature for 4 hours. The reaction mixture was diluted with $CHCl_3$ (20 mL) and transferred to a separatory funnel. The mixture was washed with water (1 × 10 mL) and saturated NaCl solution

(3x20 mL). The organic phase was dried over anhydrous Na_2SO_4 and concentrated in vacuo. The crude product was purified by flash column chromatography (eluent – EtOAc/Hex, v/v 3:1).

Yield: 30 mg (72%). White solid. M.p. > 250 °C.

^{1}H NMR (300 MHz, $CDCl_3$) δ ppm 8.27 (d, *J*=8.7 Hz, 2 H), 7.30 - 7.44 (m, 5 H), 6.43 - 6.54 (m, 2 H), 5.00 (dd, *J*=14.2, 4.8 Hz, 1 H), 3.74 (d, *J*=15.5 Hz, 1 H), 3.55 - 3.70 (m, 4 H), 3.40 - 3.49 (m, 1 H), 3.28 (dd, *J*=15.4, 3.3 Hz, 1 H)

^{13}C NMR (75 MHz, $CDCl_3$) δ ppm 181.5, 172.8, 148.2, 139.8, 132.0, 129.5, 129.4, 129.2, 127.4 , 124.1, 76.0, 57.4, 52.6, 48.9, 31.2.

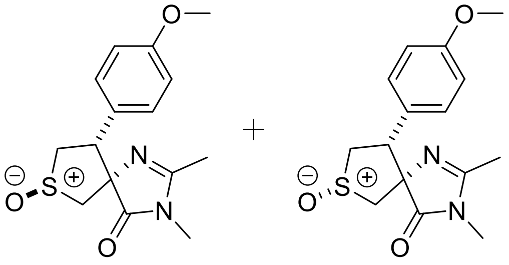

13e

***cis*-9-(4-methoxyphenyl)-2,3-dimethyl-7-thia-1,3-diazaspiro[4.4]non-1-en-4-one 7-oxide (13e)**

Compound 9e (0.1 mmol, 1 equiv) was dissolved in MeOH (2 mL) and H_2O_2 30% (0.41 mL, 40 equiv) was added to the solution. The reaction mixture was stirred at room temperature for 4 hours. The reaction mixture was diluted with $CHCl_3$ (20 mL) and transferred to a separatory funnel. The mixture was washed with water (1 × 10 mL) and saturated NaCl solution (3 × 20 mL). The organic phase was dried over anhydrous Na_2SO_4 and concentrated in vacuo. The crude product was purified by flash column chromatography (eluent – $CHCl_3$/MeOH, v/v 25:1).

Yield: 19 mg (63%). White viscous paste.

The compound was isolated as a diasteriomeric mixture (4:1). The description of the major isomer is given below.

^{1}H NMR (300 MHz, $CDCl_3$) δ ppm 7.09 (d, *J*=8.6 Hz, 2 H), 6.77 (d, *J*=8.7 Hz, 2 H), 4.49 (dd, *J*=13.8, 5.4 Hz, 1 H), 3.78 (s, 3 H), 3.68 (d, *J*=13.8 Hz, 1 H), 3.56 (t, *J*=13.6 Hz, 1 H), 3.27 (dd, *J*=13.4, 5.3 Hz, 1 H), 3.14 (dd, *J*=13.9, 0.8 Hz, 1 H), 2.78 (s, 3 H), 1.95 (s, 3 H)

^{13}C NMR (75 MHz, $CDCl_3$) δ ppm 180.2, 161.4, 159.2, 129.5, 124.8, 113.2, 79.3, 63.4, 56.2, 55.1, 52.7, 26.6, 15.1.

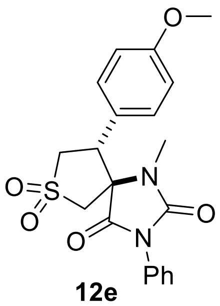

12e

***trans*-9-(4-Methoxyphenyl)-1-methyl-3-phenyl-7-thia-1,3-diazaspiro[4.4]nonane-2,4-dione 7,7-dioxide (12e)**

Compound 7e (0.1 mmol, 1 equiv) was dissolved in MeOH (2 mL) and H_2O_2 30% (5 equiv, 52 µL) was added to the solution. The reaction mixture was stirred at room temperature overnightand and subsequently concentrated in vacuo. The crude product was purified by flash column chromatography (eluent – EtOAc/Hex, v/v 1:1).

Yield: 22 mg (55%). White solid. M.p. 210-212 °C.

^{1}H NMR (300 MHz, $CDCl_3$) δ ppm 7.29 - 7.36 (m, 3 H), 7.11 (m, *J*=8.7 Hz, 2 H), 6.90 (m, *J*=8.6 Hz, 2 H), 6.69 - 6.79 (m, 2 H), 4.05 - 4.23 (m, 2 H), 3.88 (d, *J*=14.9 Hz, 1 H), 3.80 (s, 3 H), 3.35 - 3.49 (m, 2 H), 3.25 (s, 3 H)

^{13}C NMR (75 MHz, $CDCl_3$) δ ppm 171.4, 160.3, 153.9, 130.4, 129.1, 129.0, 128.6, 125.9, 122.6, 114.6, 68.8, 55.4, 53.7, 52.8, 44.9, 25.3.

5. X-ray crystallography

Single crystal X-ray studies of **6e**, **7d**, **8e** and **9e** were carried out in Center for Collective Use of INEOS RAS with Bruker APEX II diffractometer and APEX3 software.[5] The data collected were then integrated with SAINT. SADABS was used for scaling, empirical absorption corrections and the generation of data files for structure solution and refinement.

The structures were solved by dual-space algorithm and refined in anisotropic approximation for non-hydrogen atoms against $F^2(hkl)$. Hydrogen atoms of methyl, methylene and aromatic fragments were calculated according to those idealized geometry and refined with constraints applied to C–H lengths and equivalent displacement parameters. All structures were solved with the ShelXT[6] program and refined with the ShelXL[7] program. Molecular graphics was drawn using OLEX2[8] program.

Table S3. Crystallographic data for **6e**, **7d**, **8e** and **9e**

Compound	**6e**	**7d**	**8e**	**9e**
Formula moiety	$C_{14}H_{15}NO_3S_2$	$C_{19}H_{17}N_3O_3S_2$	$C_{14}H_{15}NO_2S_3$	$C_{15}H_{18}N_2O_2S$
Brutto formula	$C_{14}H_{15}NO_3S_2$	$C_{19}H_{17}N_3O_3S_2$	$C_{14}H_{15}NO_2S_3$	$C_{15}H_{18}N_2O_2S$
Formula weight	309.39	399.47	325.45	290.37
Diffractometer	Bruker APEX-II CCD	Bruker APEX-II CCD	Bruker APEX-II CCD	Bruker APEX-II CCD
Scan mode	ϕ and ω scans	ϕ and ω scans	ϕ and ω scans	ω and ϕ scans
Anode [Wavelength, Å]	MoKα [0.71073] ?	MoKα [0.71073] sealed tube	MoKα [0.71073] ?	MoKα [0.71073] sealed tube
Crystal Dimensions, mm	0.06 × 0.17 × 0.2	0.34 × 0.34 × 0.34	0.08 × 0.1 × 0.15	0.24 × 0.31 × 0.36
Crystal color	clear colourless	colourless	clear light yellow	colourless
Crystal system	monoclinic	orthorhombic	monoclinic	orthorhombic
a, Å	26.025(4)	9.3620(13)	8.9341(6)	7.7800(7)
b, Å	8.2676(12)	15.150(2)	9.2396(7)	18.5843(16)
c, Å	14.964(2)	26.466(4)	18.5187(14)	20.5791(18)
α, °	90	90	90	90
β, °	120.303(4)	90	99.163(2)	90
γ, °	90	90	90	90
Volume, Å^3	2779.8(7)	3754.0(9)	1509.17(19)	2975.4(5)
Density, gcm^{-3}	1.479	1.414	1.432	1.296
Temperature, K	120	120	120	120
T_{min}/T_{max}	0.5323/0.7461	0.6278/0.7461	0.6851/0.7461	0.6923/0.7461
μ, mm^{-1}	0.389	0.309	0.491	0.220

Space group	C12/c1	Pbca	$P12_1/n1$	Pbca
Z	8	8	4	8
F(000)	1296	1664	680	1232
Reflections collected	17844	34413	12815	48812
Independent reflections	4271	5741	4470	4570
Reflections (I>2σ(I))	2799	3762	3822	3306
Parameters	184	246	183	184
R_{int}	0.0841	0.0716	0.0193	0.0429
$2\theta_{min}$ - $2\theta_{max}$, °	3.626 - 61.344	5.330 - 61.108	4.456 - 61.060	3.958 - 61.260
wR_2 (all reflections)	0.1522	0.1104	0.0754	0.0989
R_1(I>σ(I))	0.0554	0.0477	0.0288	0.0364
GOF	1.018	1.026	1.061	1.092
ρ_{min}/ρ_{max}, eÅ^{-3}	-0.523/0.509	-0.324/0.347	-0.298/0.371	-0.306/0.312

6. References

1. I. V. Tiushina, A. I. Sokolov, A. Yu. Smirnov, M. A. Dubinnyi, D. S. Ivanov, A. A. Korlyukov, A. A. Mikhaylov, M. S. Baranov, *ChemistrySelect* **2024**, *9*, e202400930.

2. S. O. Zaitseva, S. V. Golodukhina, N. S. Baleeva, E. A. Levina, A. Yu. Smirnov, M. B. Zagudaylova, M. S. Baranov, *ChemistrySelect* **2018**, *3*, 8593–8596.

3. Y. G. Ermakova, T. Sen, Y. A. Bogdanova, A. Yu. Smirnov, N. S. Baleeva, A. I. Krylov, M. S. Baranov, *J. Phys. Chem. Lett.* **2018**, *9*, 1958–1963.

4. A. Thaqi, J. L. Scott, J. Gilbert, J. A. Sakoff, A. McCluskey, *European Journal of Medicinal Chemistry* **2010**, *45*, 1717–1723.

5. Bruker (2016). APEX3, RLATT, CELL_NOW, TWINABS, SAINT-Plus and SADABS. Bruker AXS Inc., Madison, Wisconsin, USA.

6. G. M. Sheldrick, *Acta Crystallogr A Found Adv* **2015**, 71, 3–8.

7. G. M. Sheldrick, *Acta Crystallogr C Struct Chem* **2015**, 71, 3–8.

8. O. V. Dolomanov, L. J. Bourhis, R. J. Gildea, J. A. K. Howard, H. Puschmann, *J Appl Crystallogr* **2009**, 42, 339–341.

7. Copies of NMR spectra

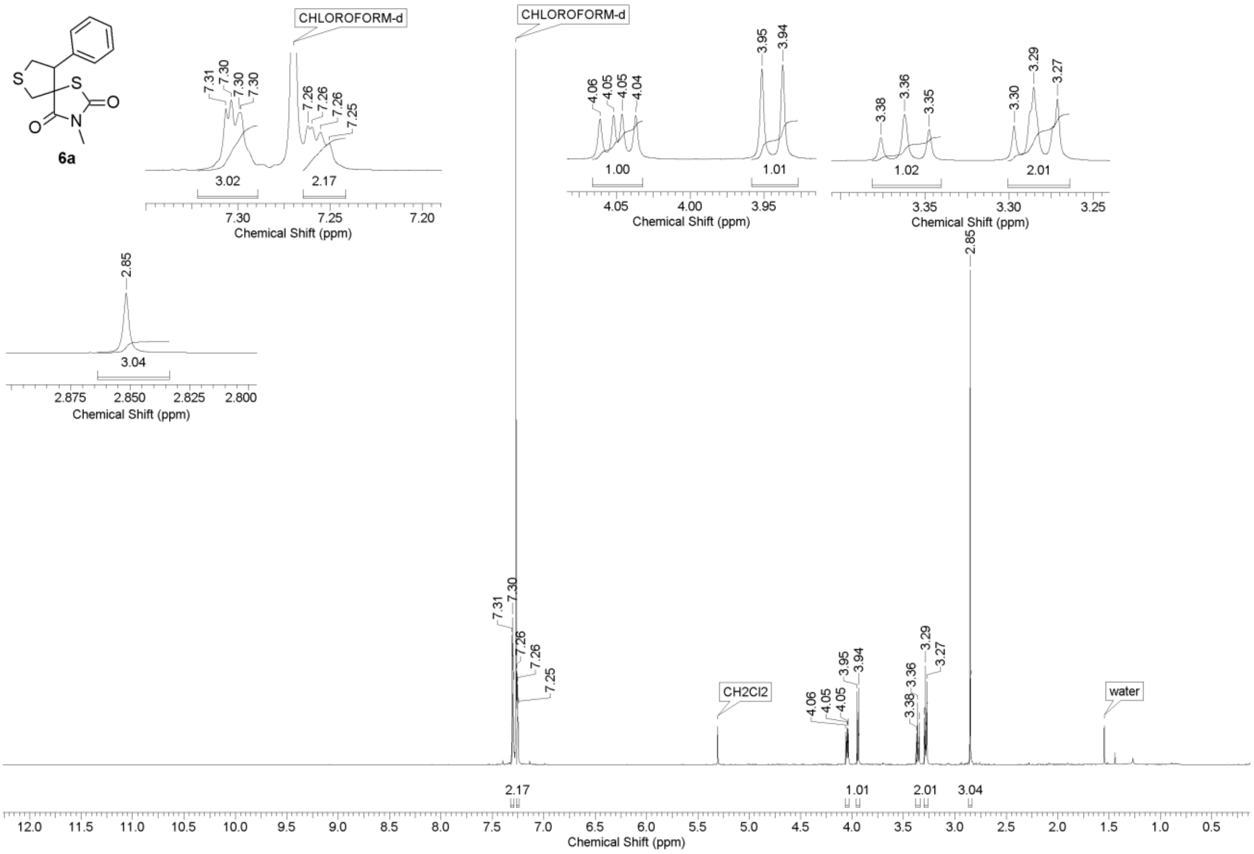

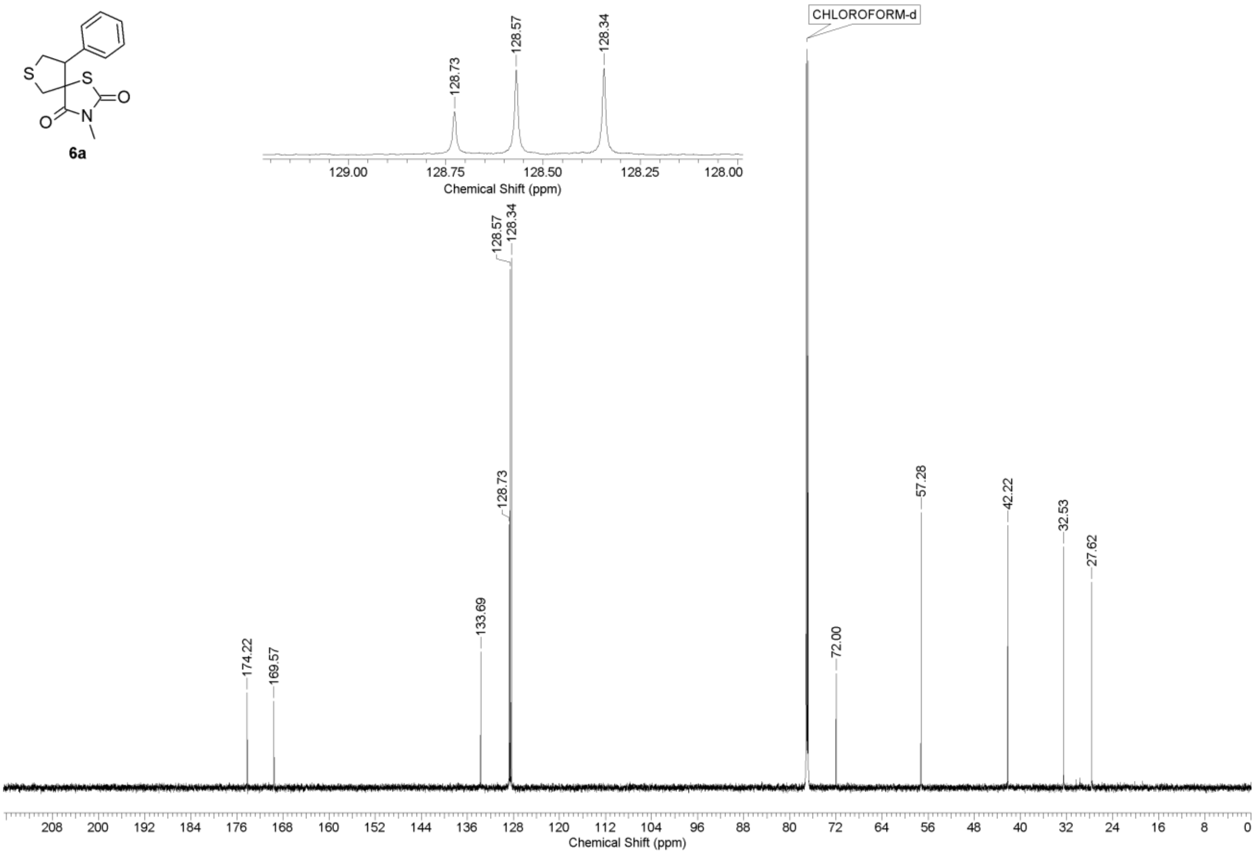

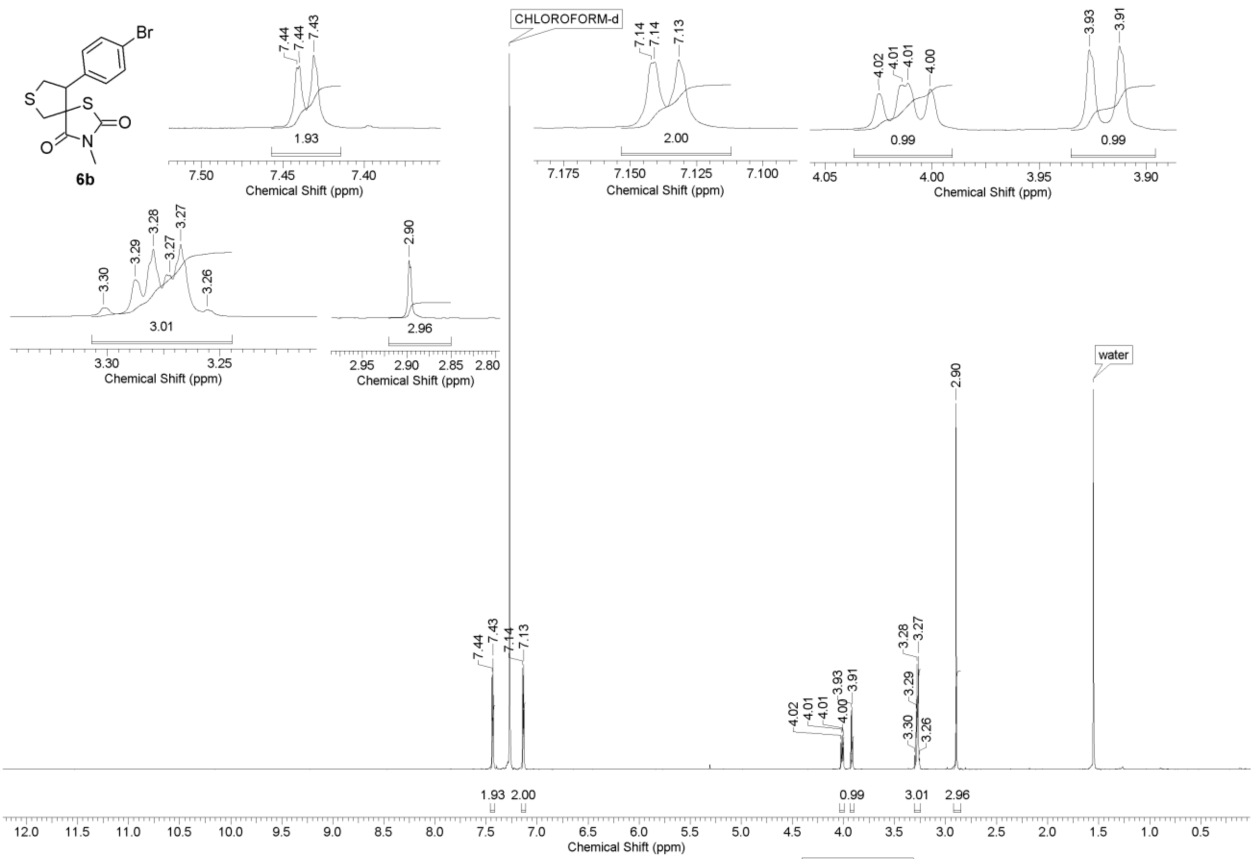
Br
S
S
O
O
N
6b
CHLOROFORM-d
water
7.44
7.44
7.43
7.14
7.14
7.13
4.02
4.01
4.01
4.00
3.93
3.91
3.30
3.29
3.28
3.27
3.27
3.26
2.90
1.93
2.00
0.99
0.99
3.01
2.96
Chemical Shift (ppm)

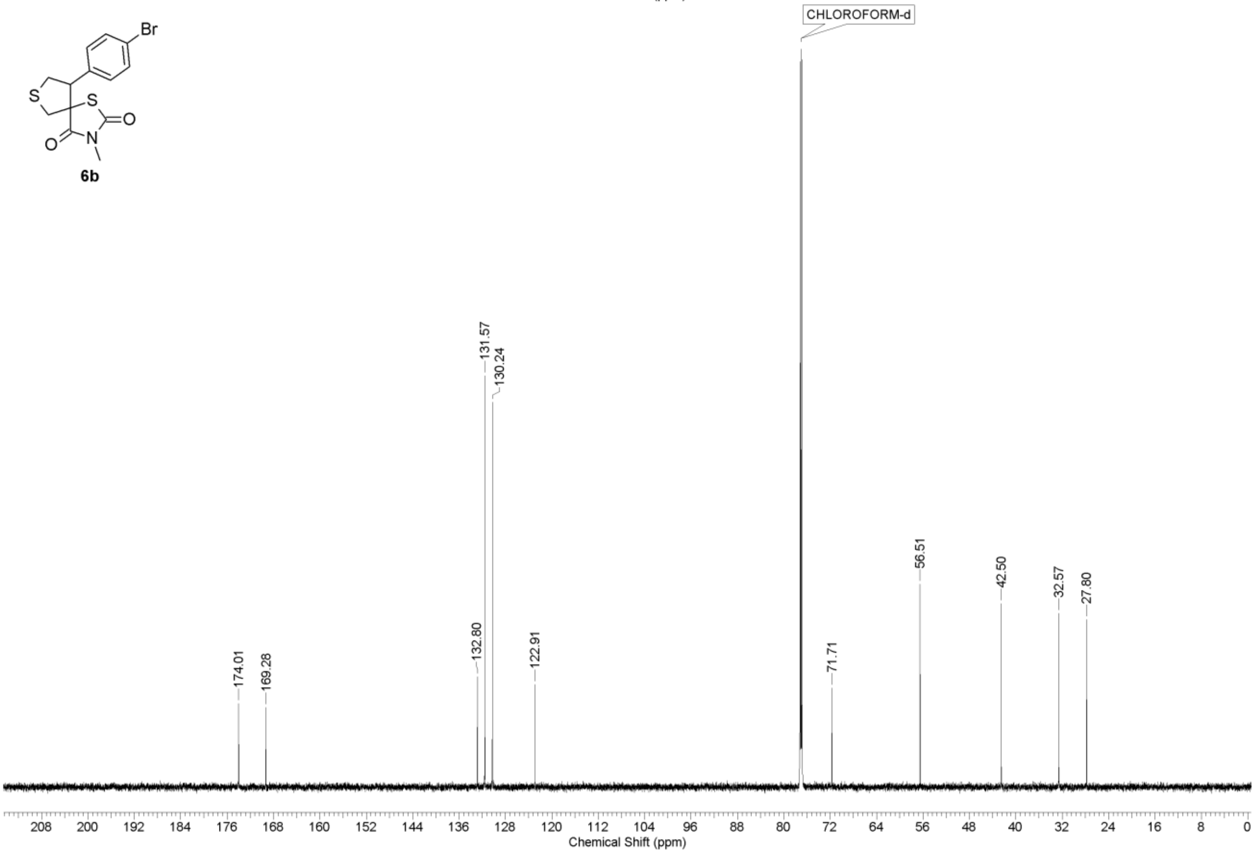
Br
S
S
O
O
N
6b
CHLOROFORM-d
174.01
169.28
132.80
131.57
130.24
122.91
71.71
56.51
42.50
32.57
27.80
Chemical Shift (ppm)

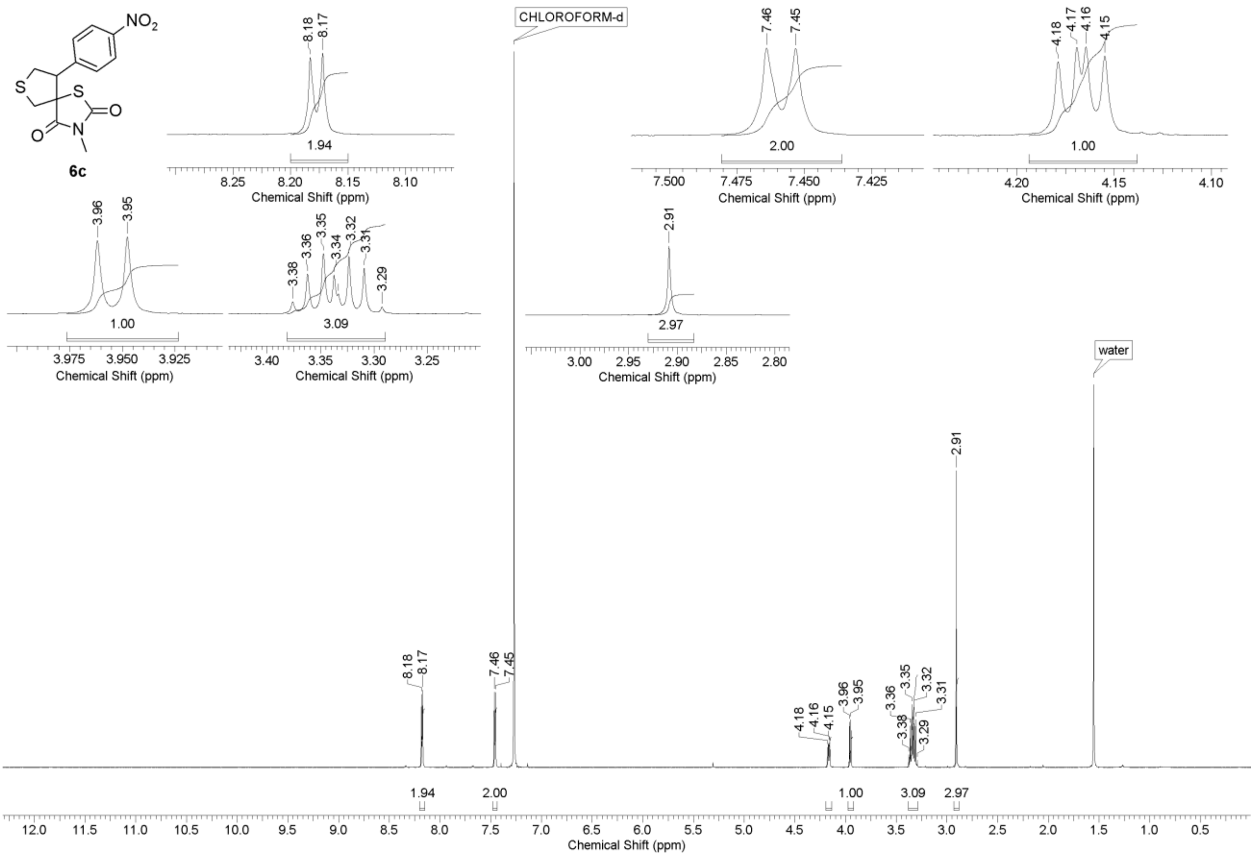
NO2
S
S
O
N
O
6c
CHLOROFORM-d
water
8.18
8.17
7.46
7.45
4.18
4.17
4.16
4.15
3.96
3.95
3.38
3.36
3.35
3.34
3.32
3.31
3.29
2.91
1.94
2.00
1.00
3.09
2.97
Chemical Shift (ppm)

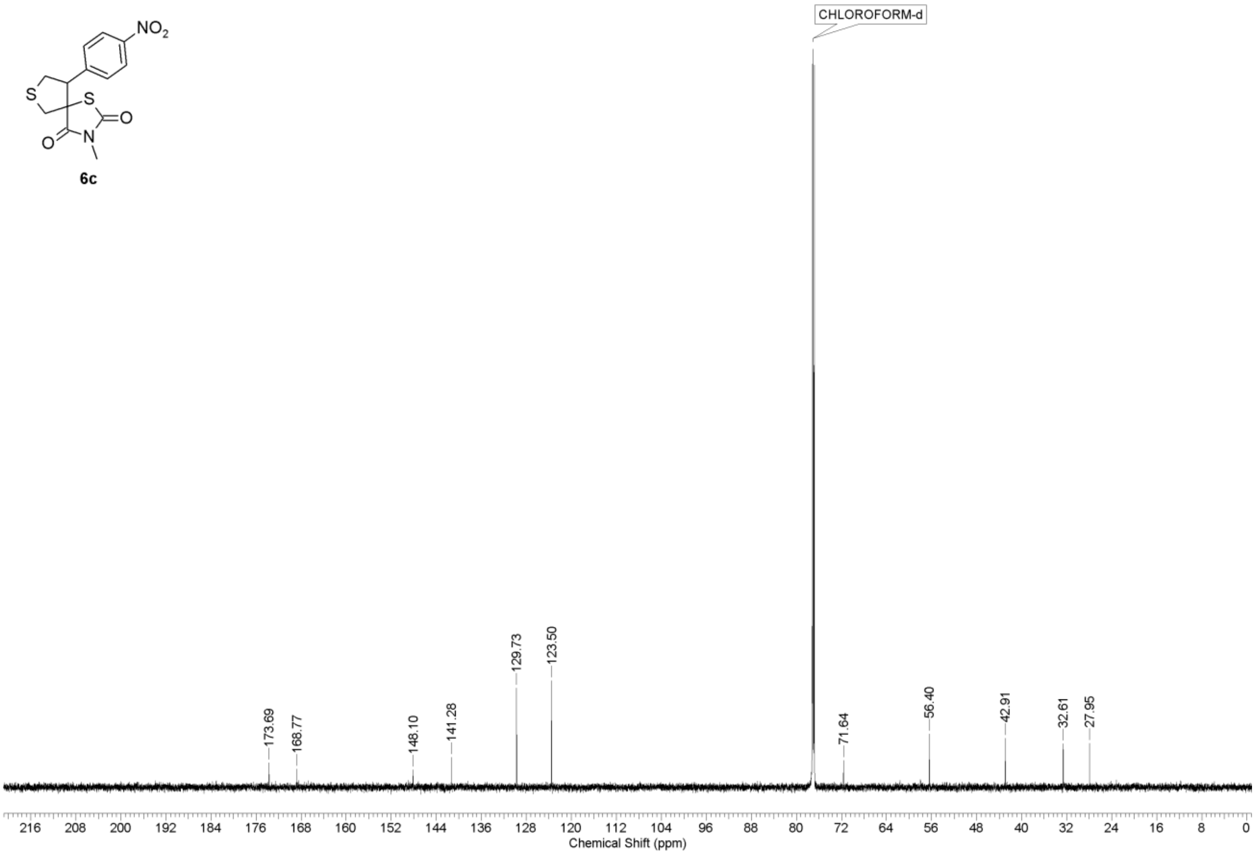
NO2
S
S
O
N
O
6c
CHLOROFORM-d
173.69
168.77
148.10
141.28
129.73
123.50
71.64
56.40
42.91
32.61
27.95
Chemical Shift (ppm)

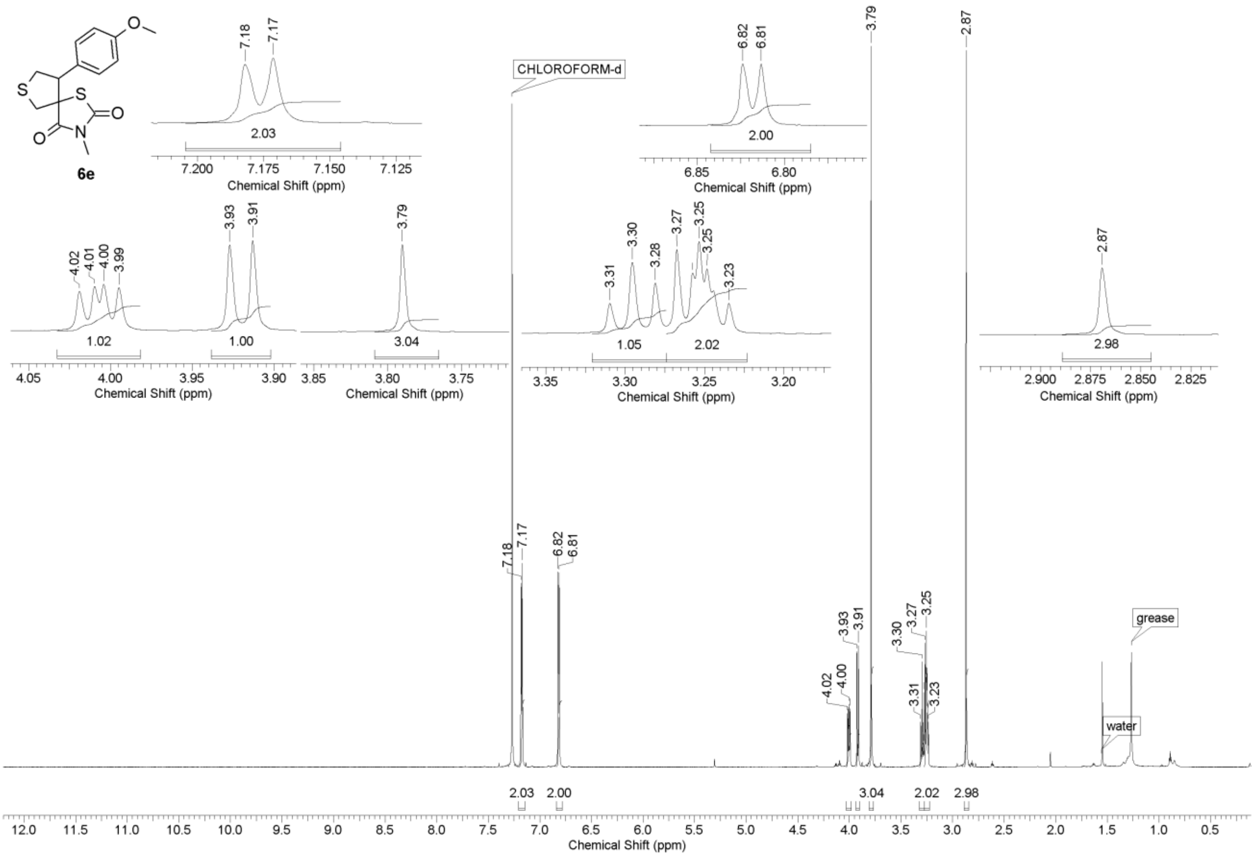

6e
CHLOROFORM-d
7.18
7.17
6.82
6.81
4.02
4.01
4.00
3.99
3.93
3.91
3.79
3.31
3.30
3.28
3.27
3.25
3.25
3.23
2.87
grease
water
2.03
2.00
1.02
1.00
3.04
1.05
2.02
2.98
Chemical Shift (ppm)

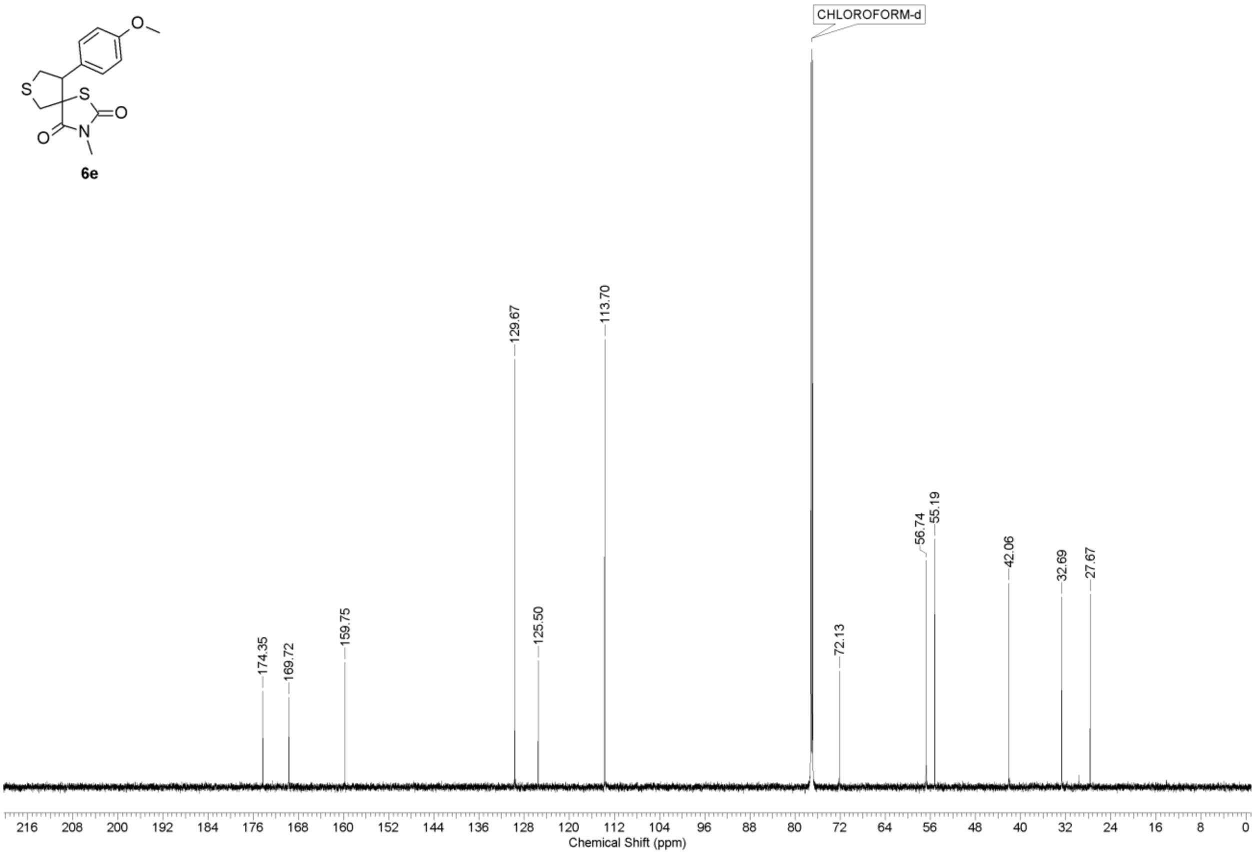

6e
CHLOROFORM-d
174.35
169.72
159.75
129.67
125.50
113.70
72.13
56.74
55.19
42.06
32.69
27.67
Chemical Shift (ppm)

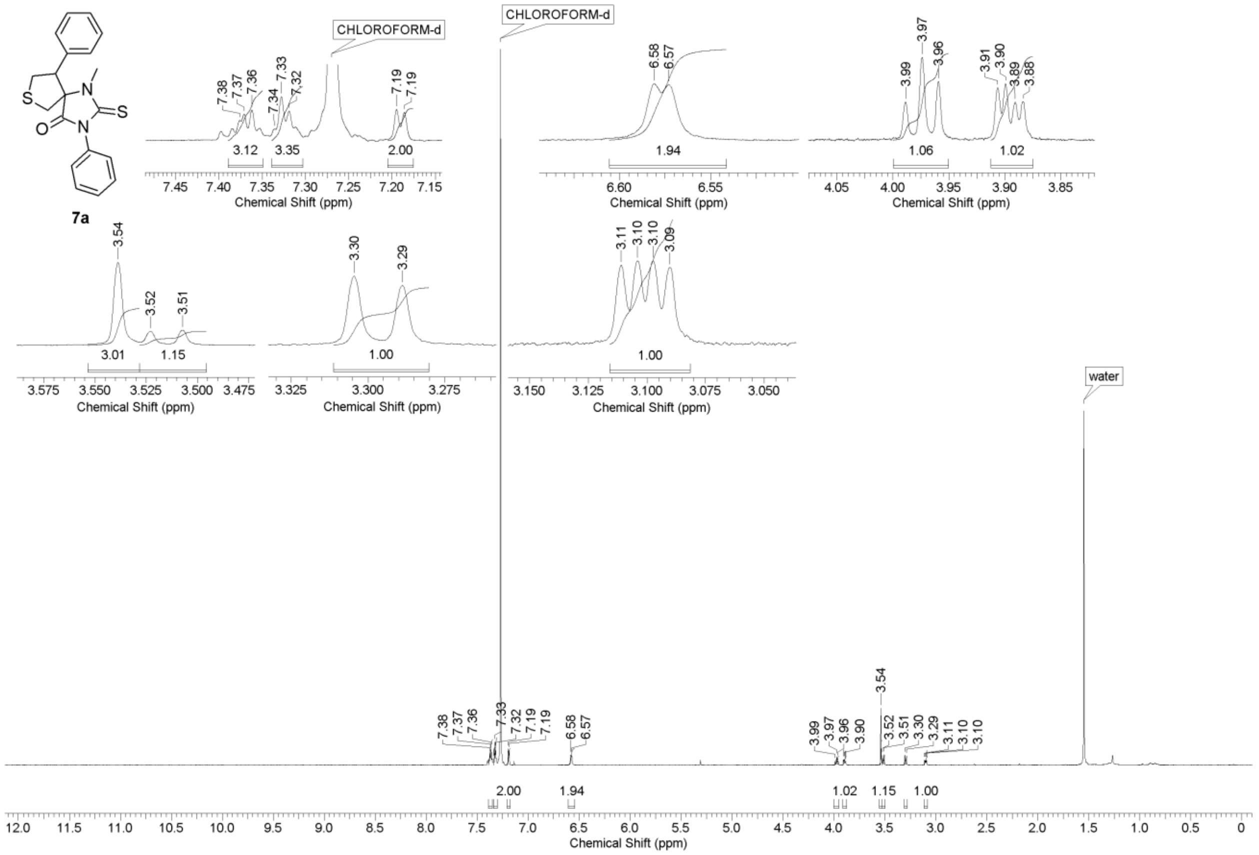
7a
CHLOROFORM-d
water
Chemical Shift (ppm)

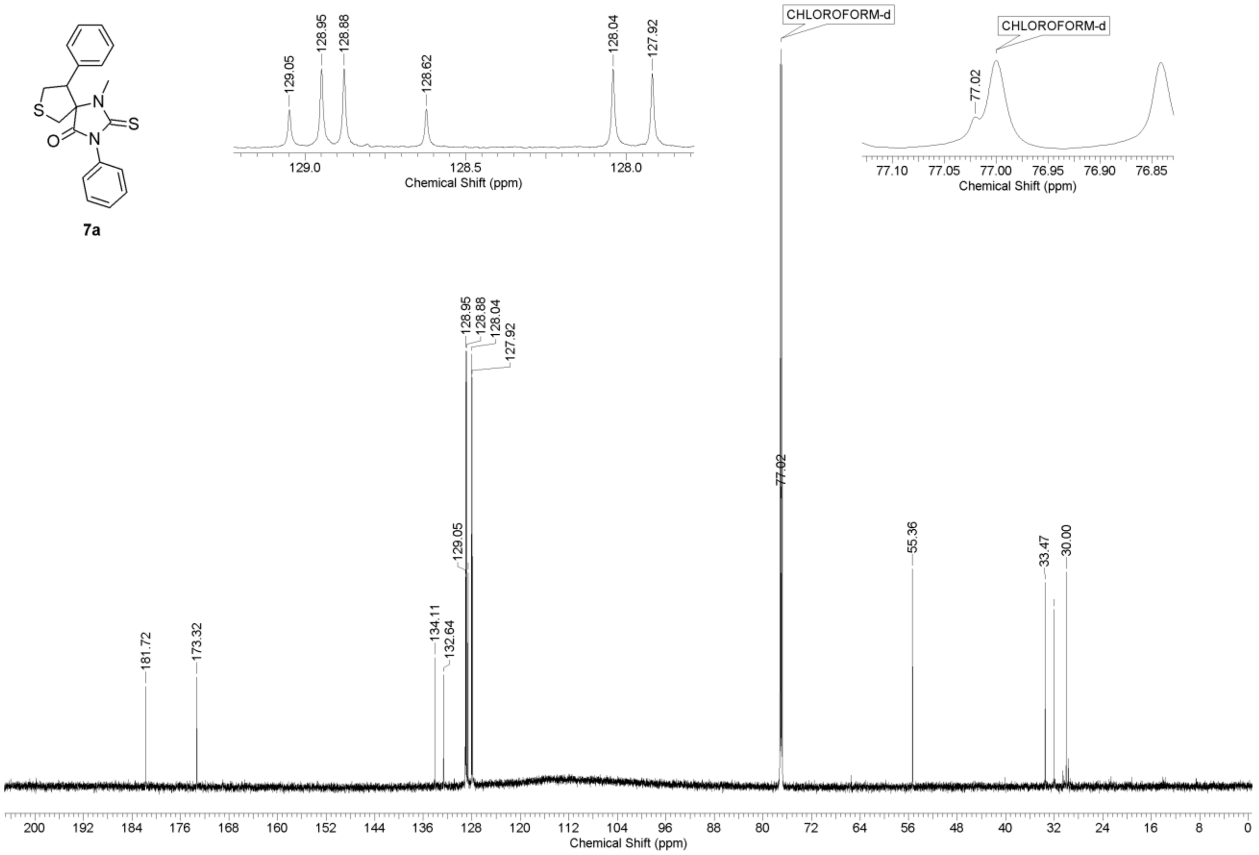
7a
CHLOROFORM-d
Chemical Shift (ppm)

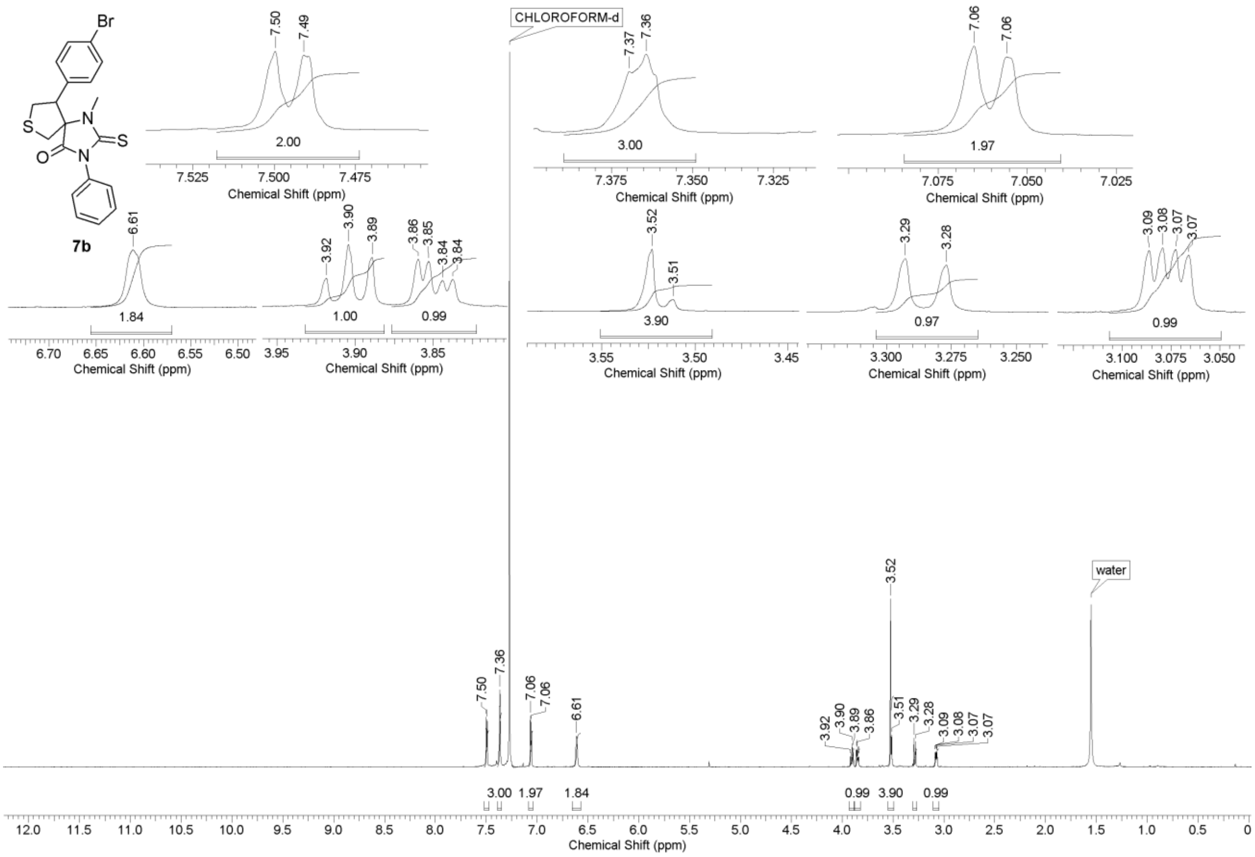

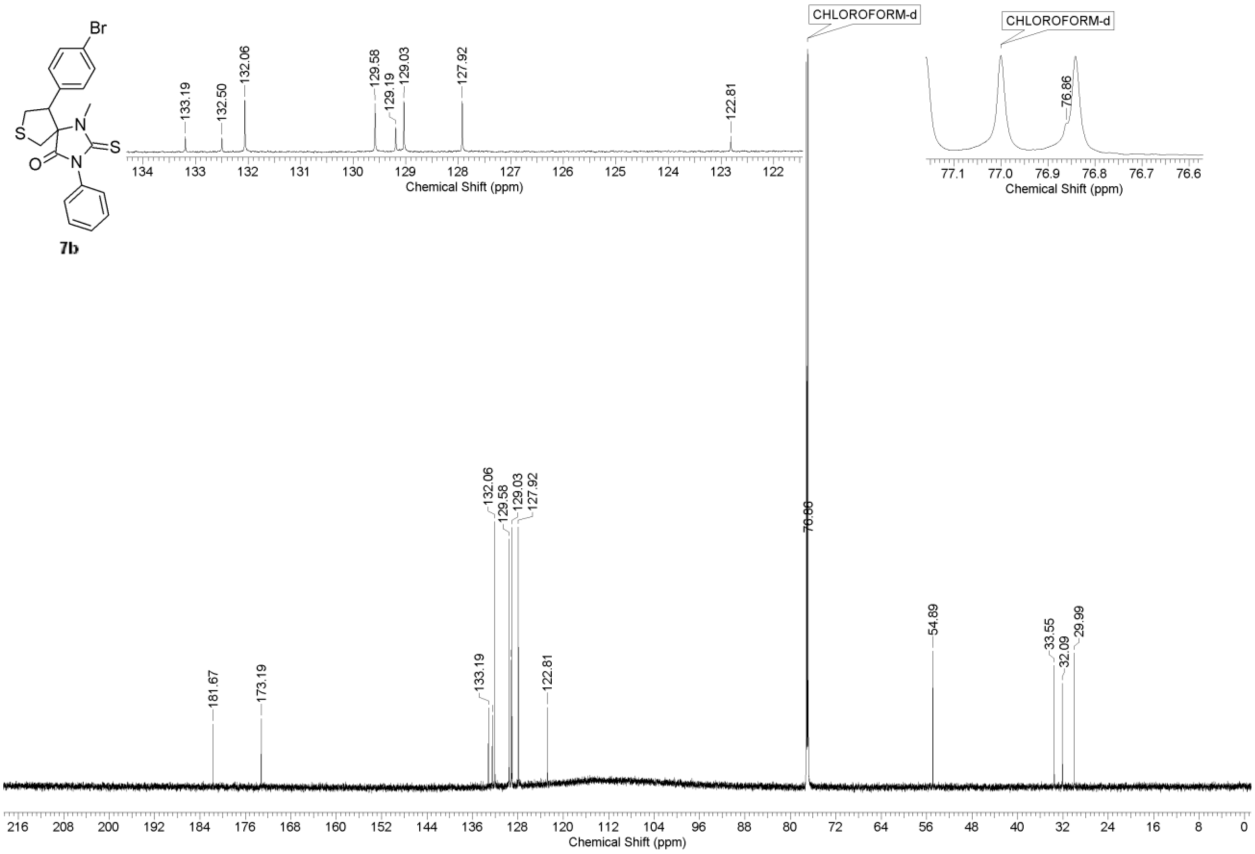

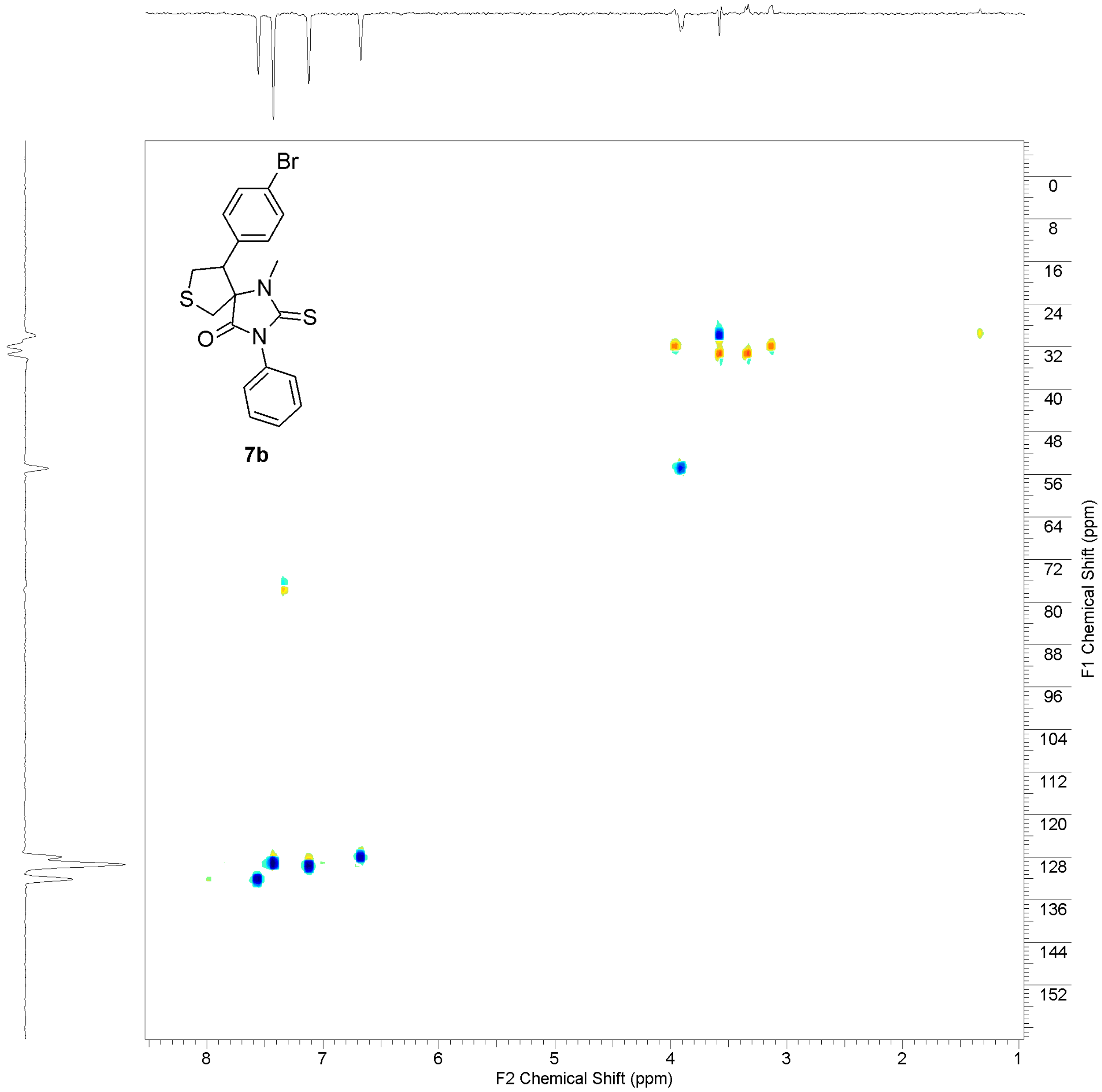

ME HSQC NMR spectrum of **6b**

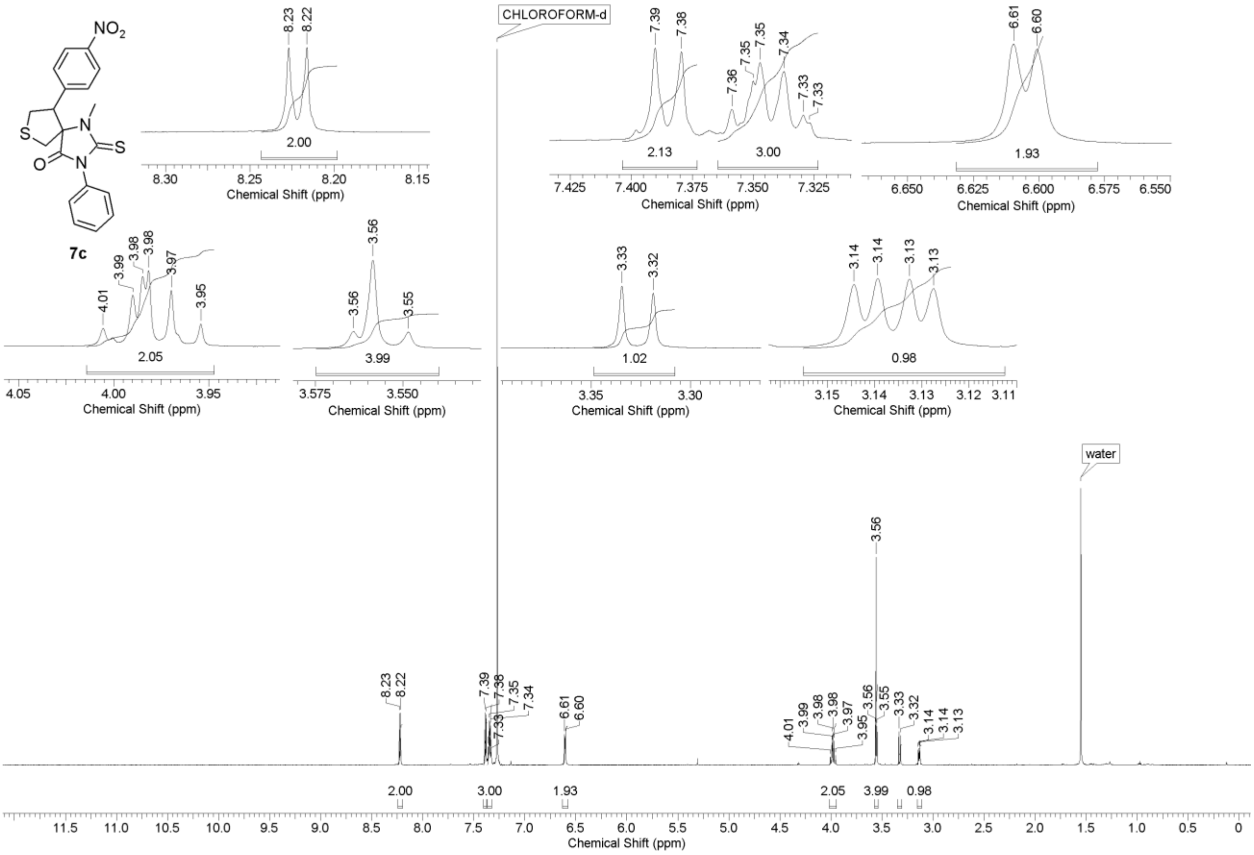

NO_2
S
N
O
S
7c
CHLOROFORM-d
water
Chemical Shift (ppm)

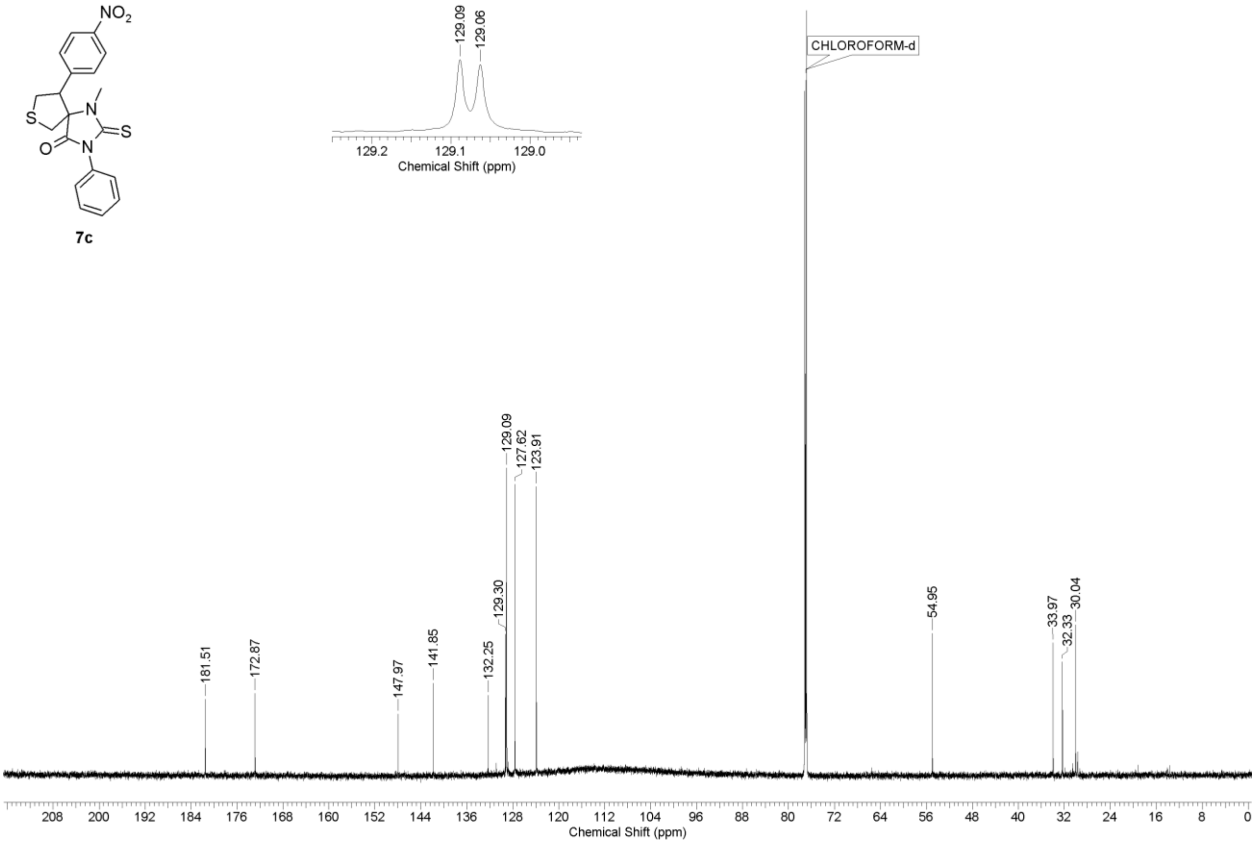

NO_2
S
N
O
S
7c
CHLOROFORM-d
Chemical Shift (ppm)

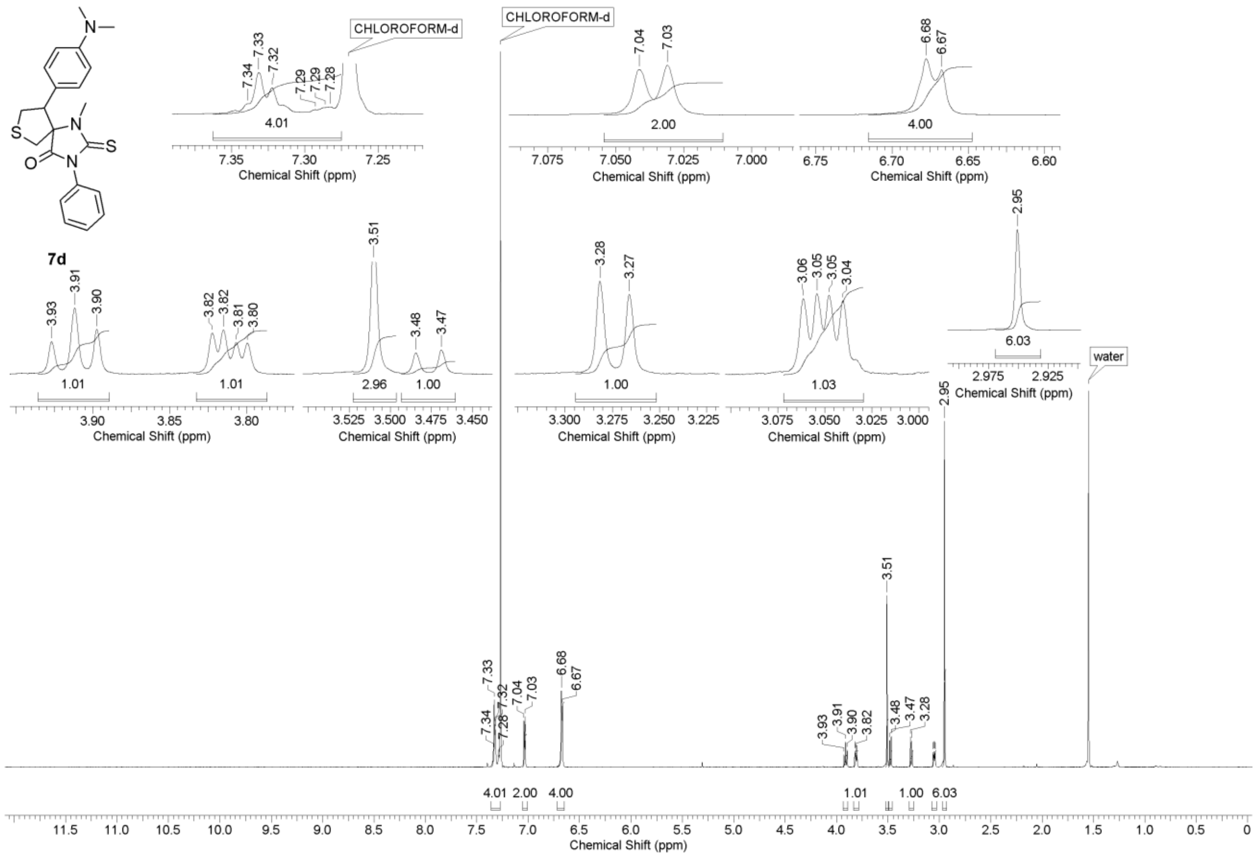
7d
CHLOROFORM-d
water
Chemical Shift (ppm)

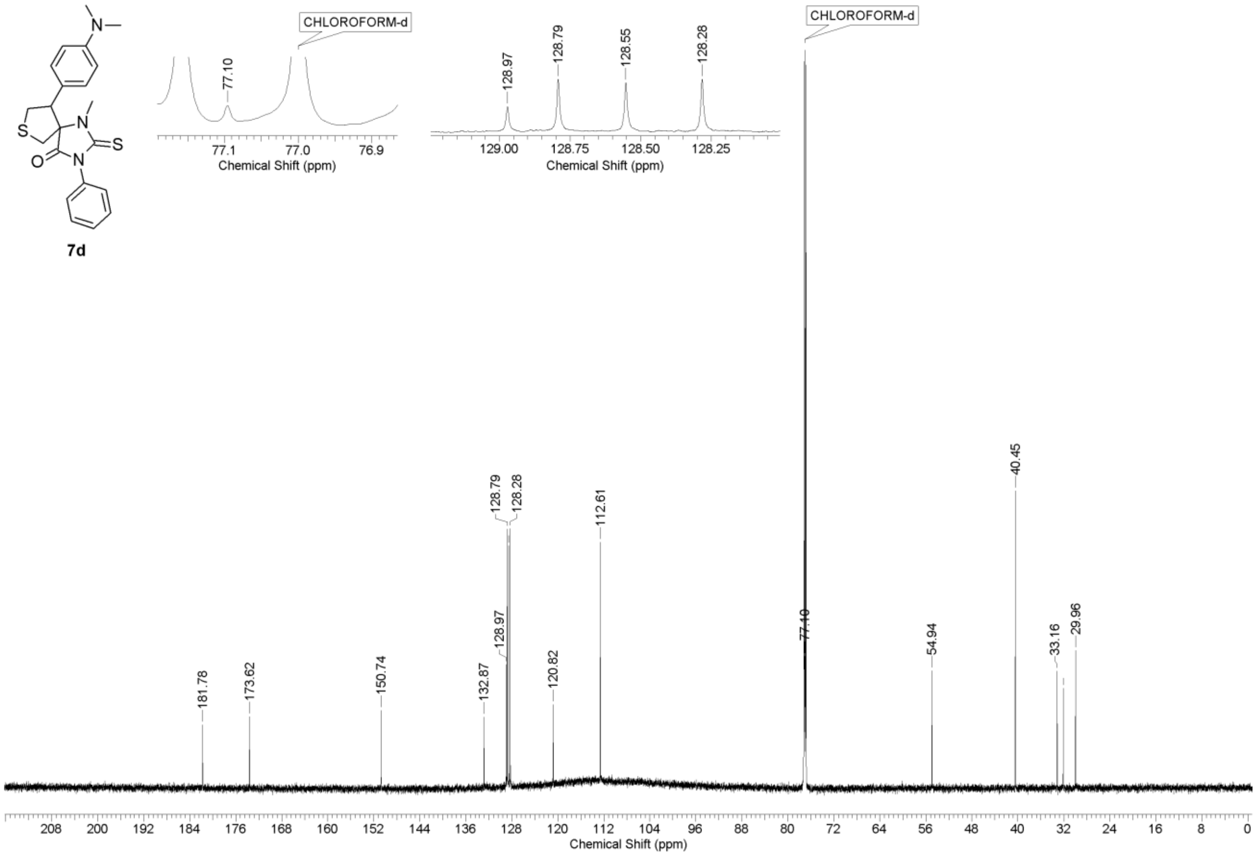
7d
CHLOROFORM-d
Chemical Shift (ppm)

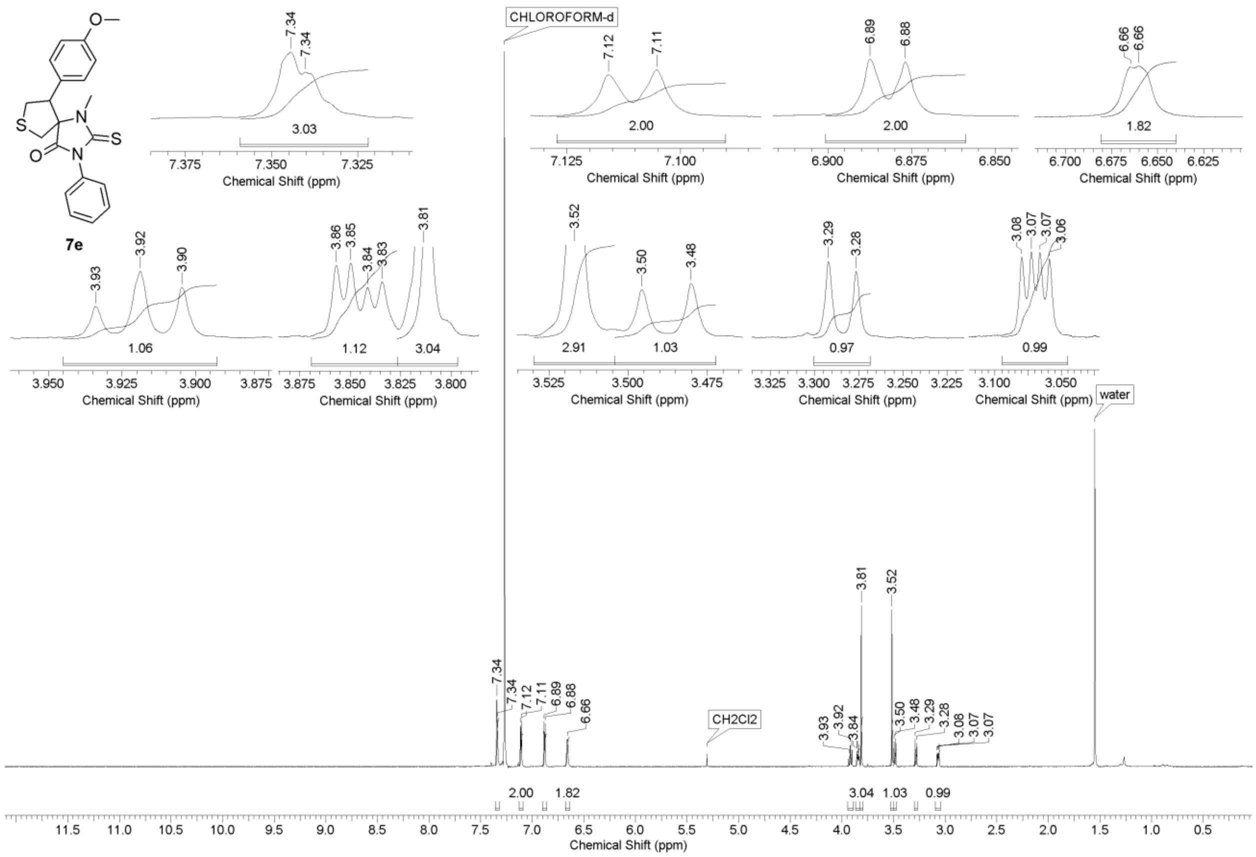
7e
CHLOROFORM-d
water
CH2Cl2
7.34
7.34
7.12
7.11
6.89
6.88
6.66
6.66
3.93
3.92
3.90
3.86
3.85
3.84
3.83
3.81
3.52
3.50
3.48
3.29
3.28
3.08
3.07
3.07
3.06
3.03
2.00
2.00
1.82
1.06
1.12
3.04
2.91
1.03
0.97
0.99
Chemical Shift (ppm)

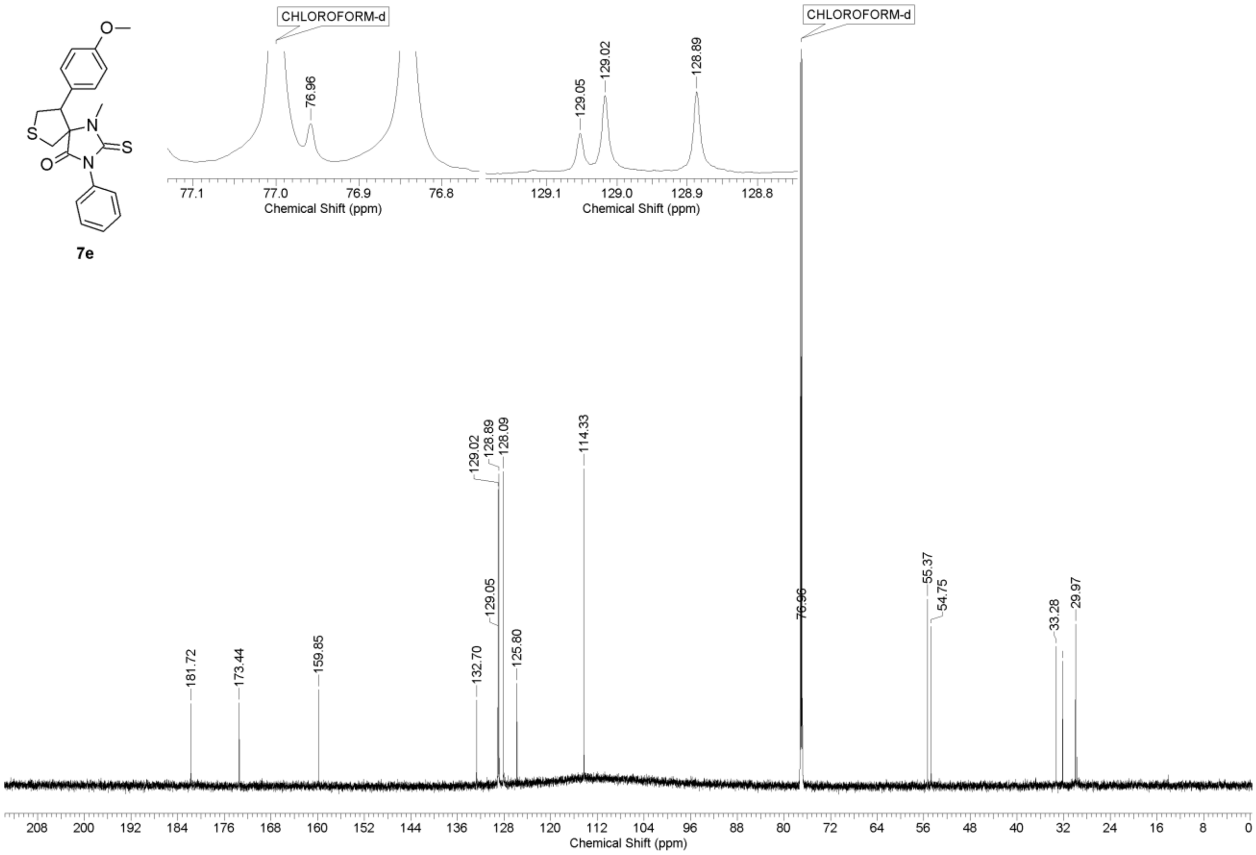
7e
CHLOROFORM-d
76.96
129.05
129.02
128.89
128.09
114.33
181.72
173.44
159.85
132.70
125.80
55.37
54.75
33.28
29.97
Chemical Shift (ppm)

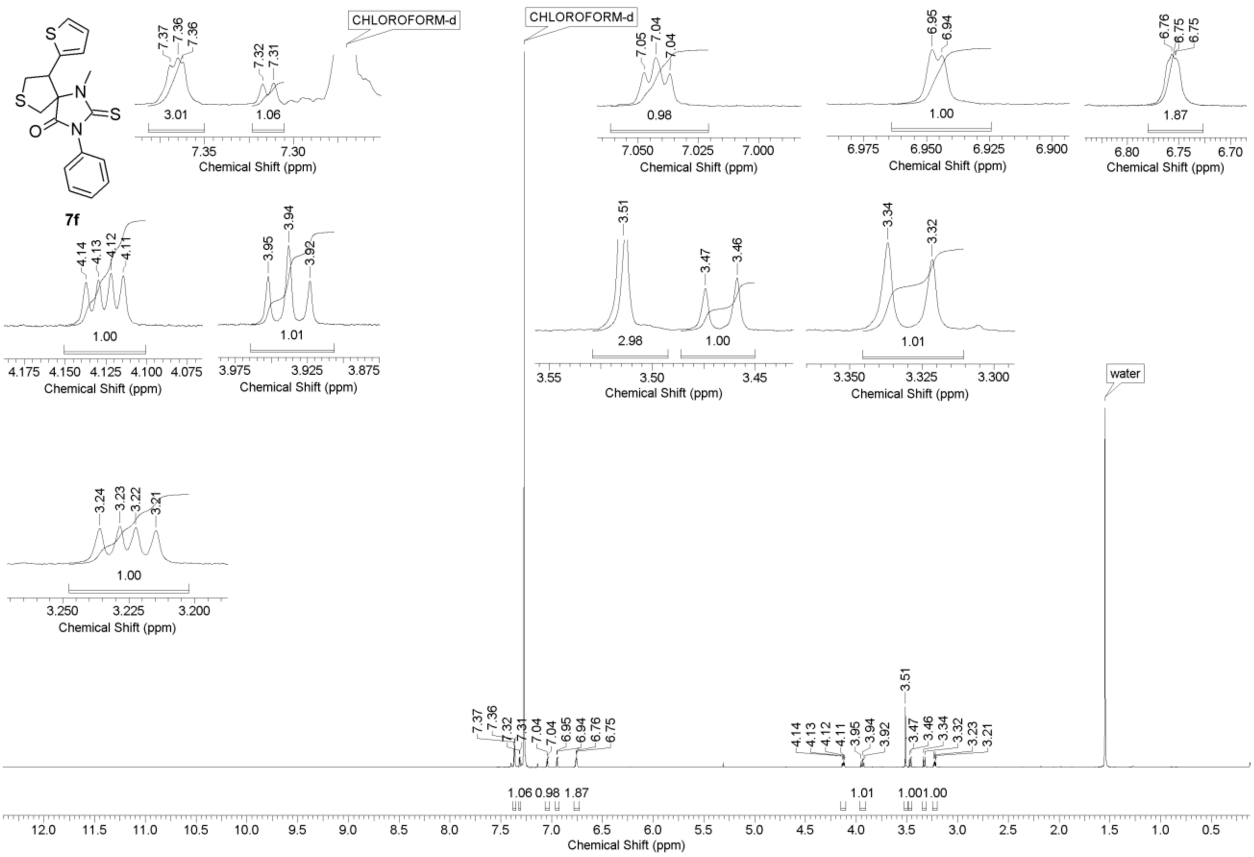
7f
CHLOROFORM-d
water
7.37 7.36 7.36 7.32 7.31
7.05 7.04 7.04
6.95 6.94
6.76 6.75 6.75
4.14 4.13 4.12 4.11
3.95 3.94 3.92
3.51 3.47 3.46
3.34 3.32
3.24 3.23 3.22 3.21
3.01 1.06 0.98 1.00 1.87 1.00 1.01 2.98 1.00 1.01 1.00
Chemical Shift (ppm)

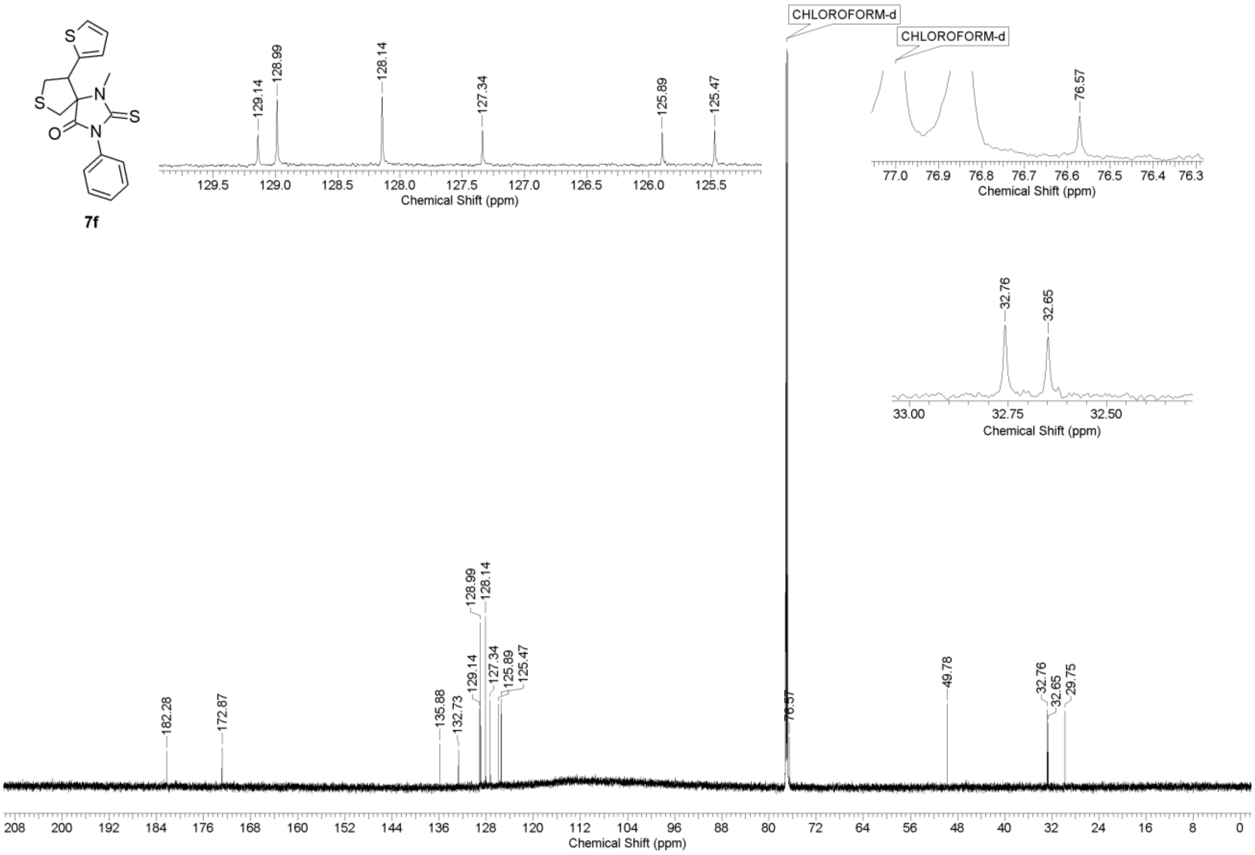
7f
CHLOROFORM-d
182.28 172.87 135.88 132.73 129.14 128.99 128.14 127.34 125.89 125.47 76.57 49.78 32.76 32.65 29.75
Chemical Shift (ppm)

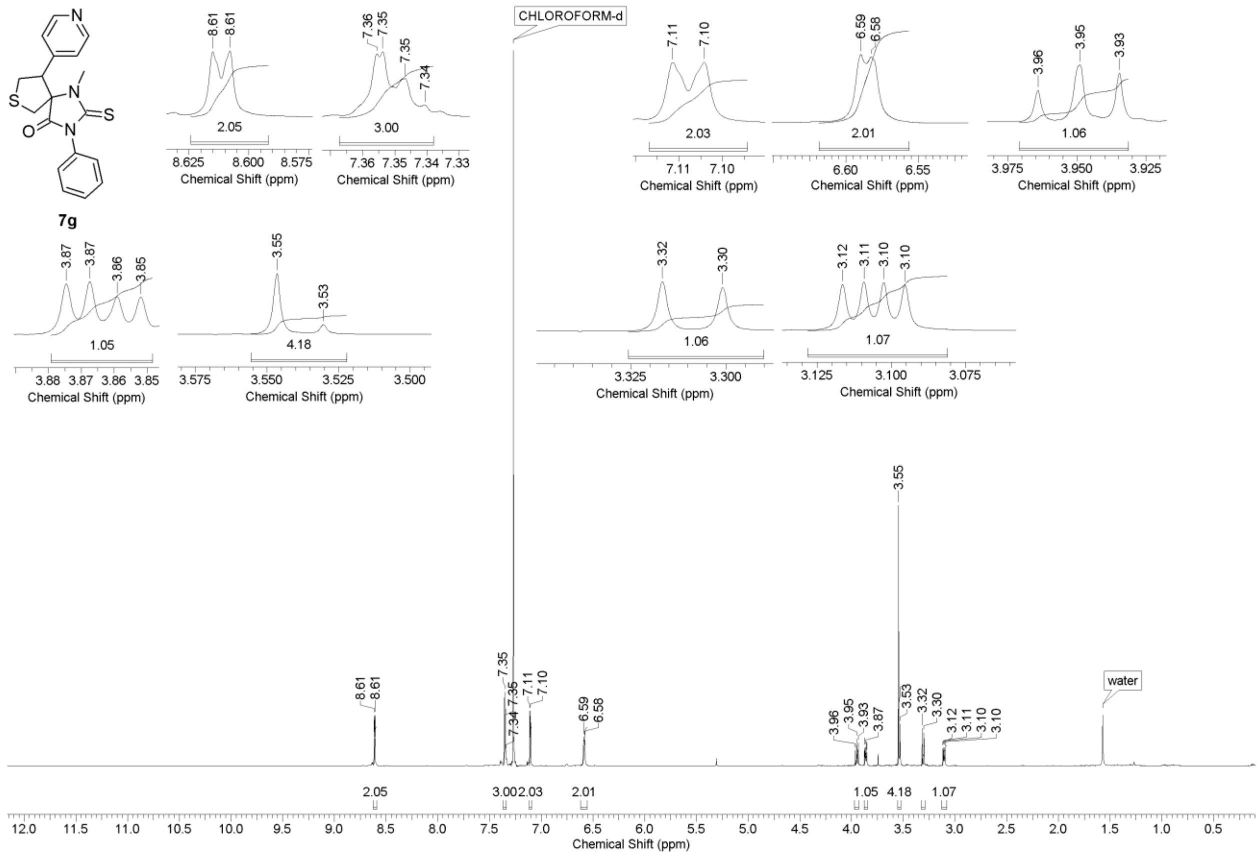

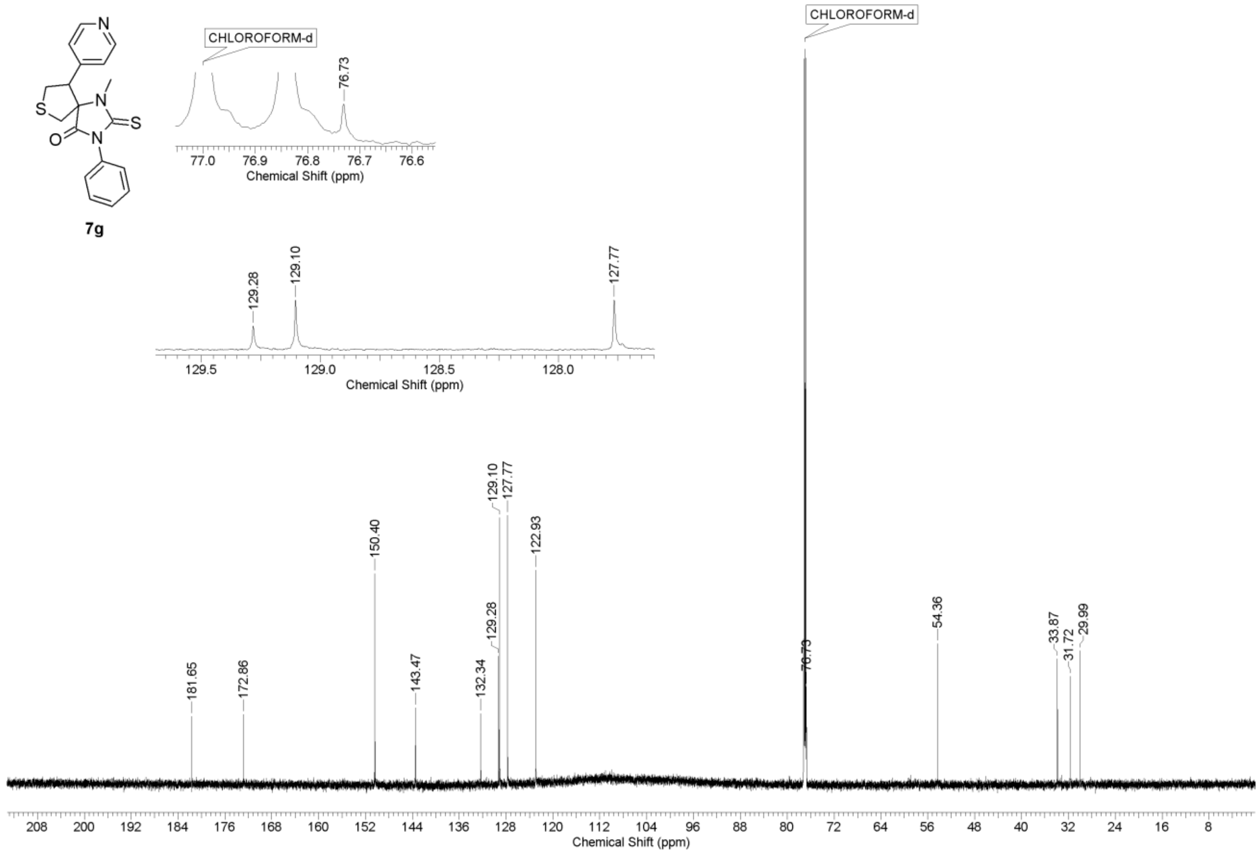

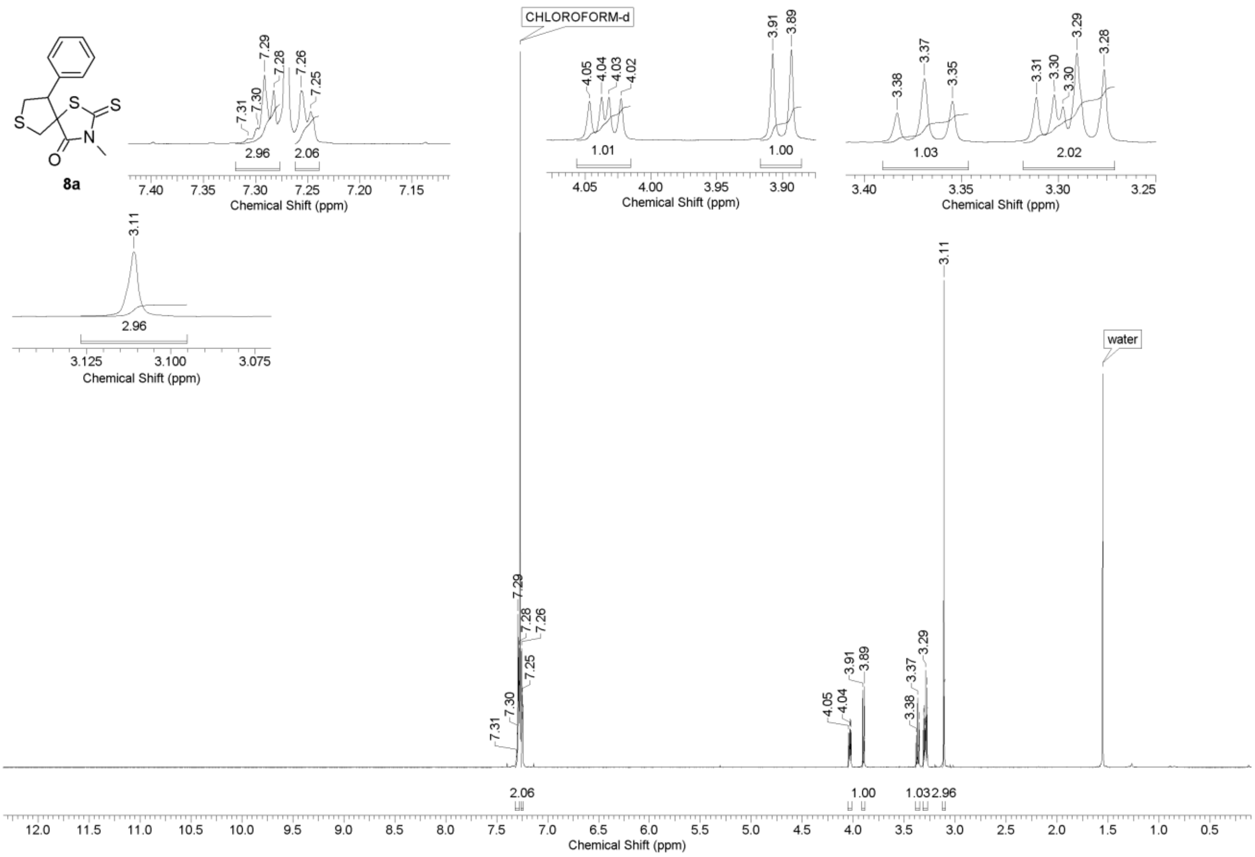
8a
CHLOROFORM-d
water
7.31
7.30
7.29
7.28
7.26
7.25
4.05
4.04
4.03
4.02
3.91
3.89
3.38
3.37
3.35
3.31
3.30
3.30
3.29
3.28
3.11
2.96
2.06
1.01
1.00
1.03
2.02
Chemical Shift (ppm)

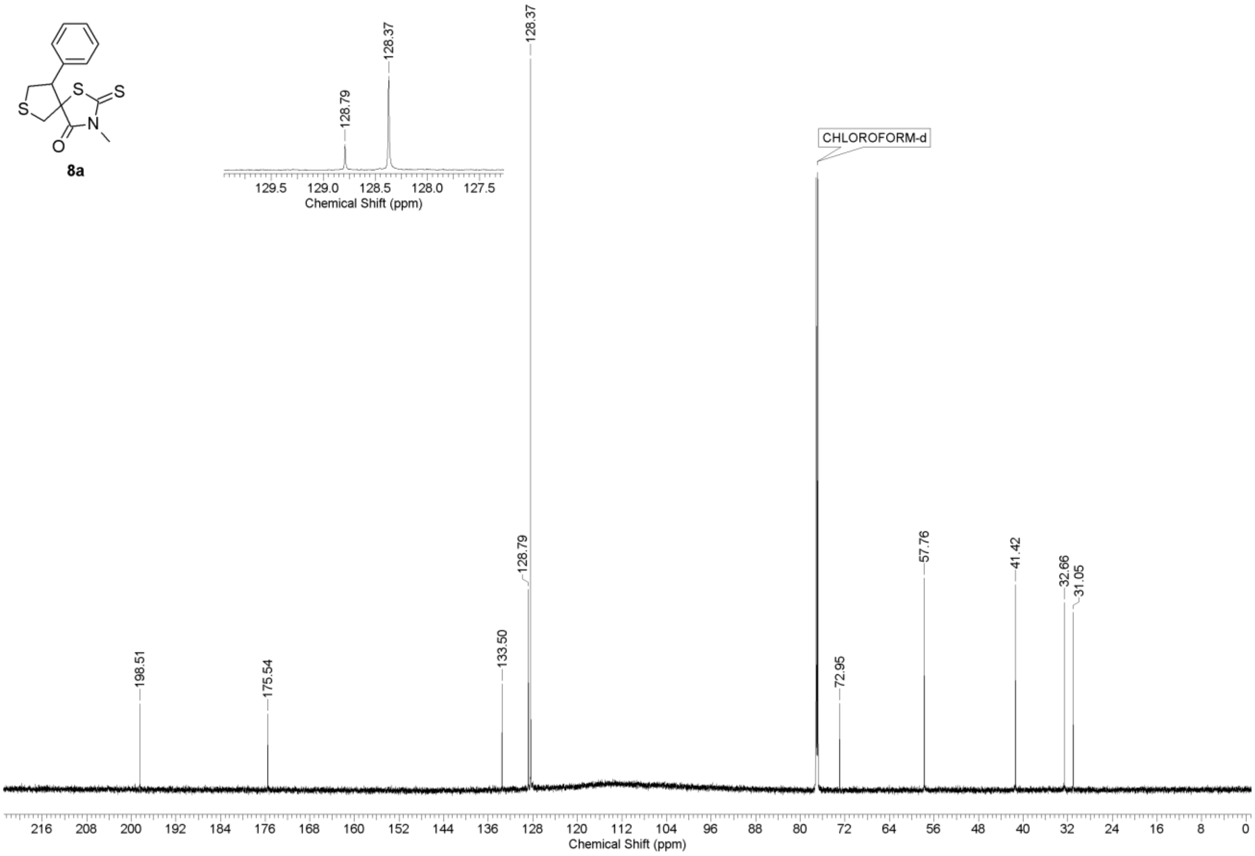
8a
CHLOROFORM-d
198.51
175.54
133.50
128.79
128.37
72.95
57.76
41.42
32.66
31.05
Chemical Shift (ppm)

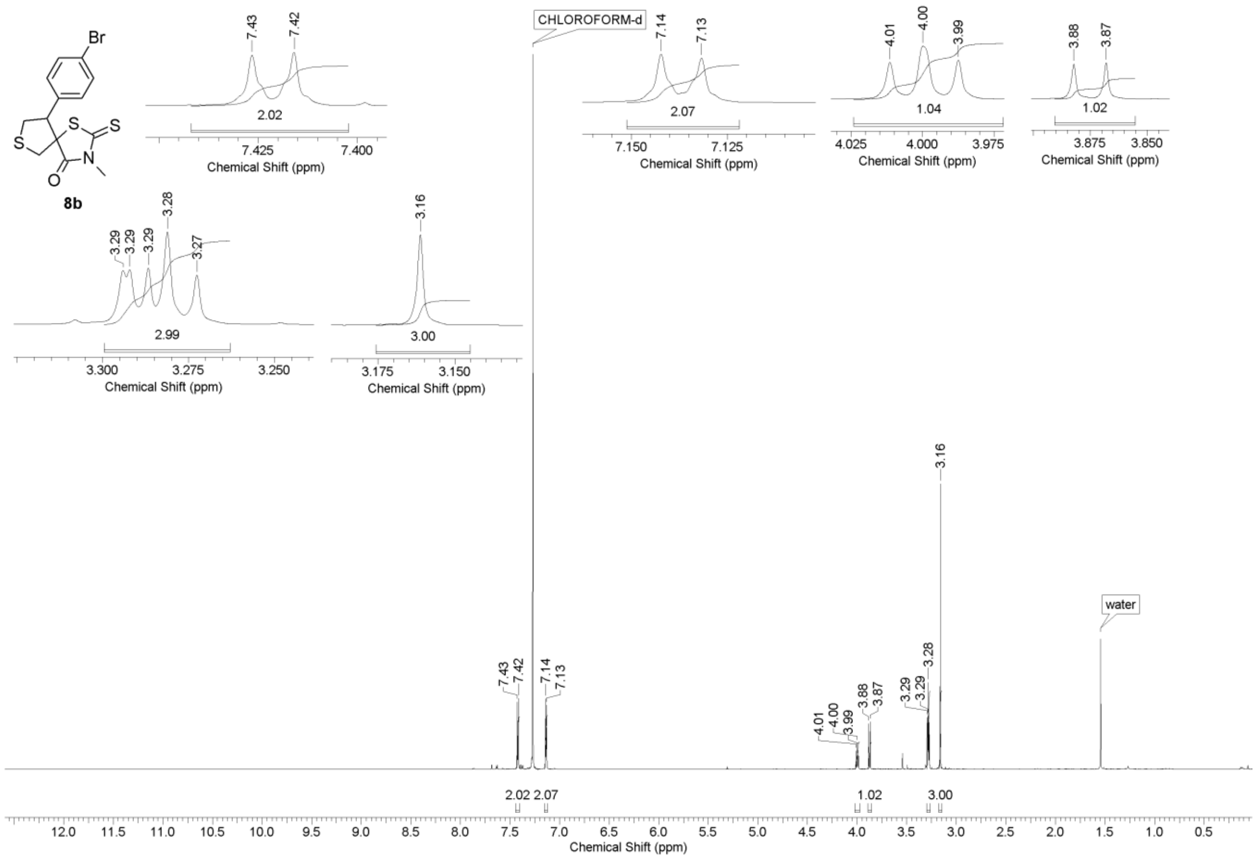

8b
CHLOROFORM-d
water
Chemical Shift (ppm)

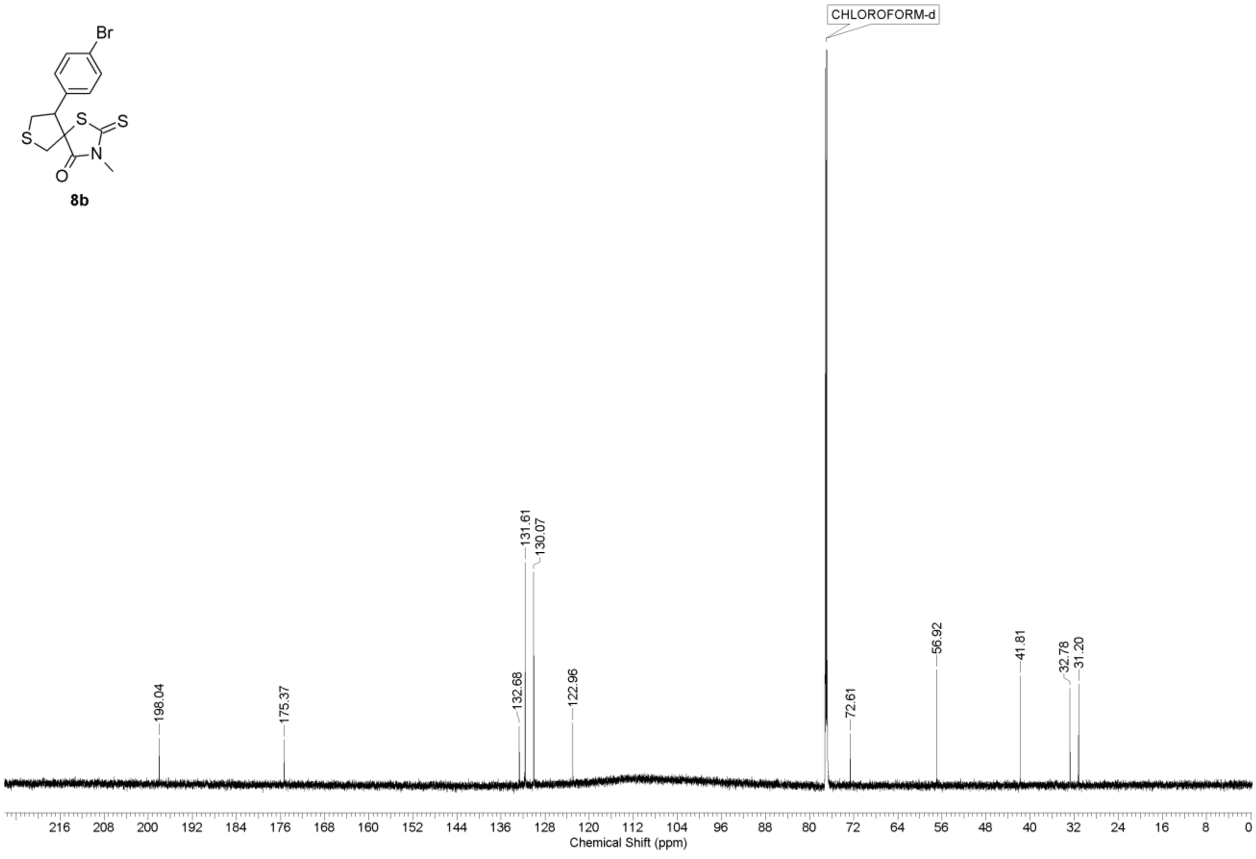

8b
CHLOROFORM-d
Chemical Shift (ppm)

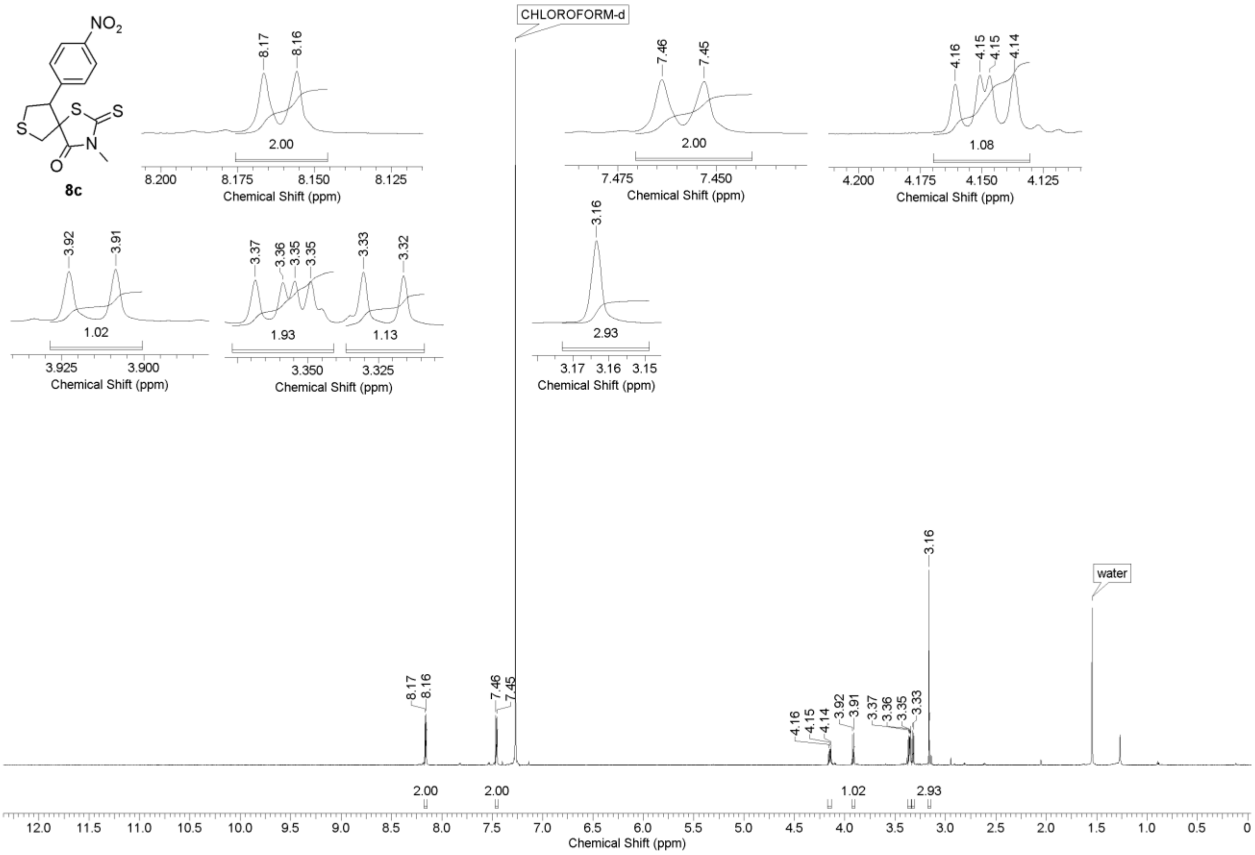
8c
CHLOROFORM-d
water
Chemical Shift (ppm)
8.17 8.16 7.46 7.45 4.16 4.15 4.14 3.92 3.91 3.37 3.36 3.35 3.33 3.16
2.00 2.00 1.02 2.93
1.08 1.93 1.13

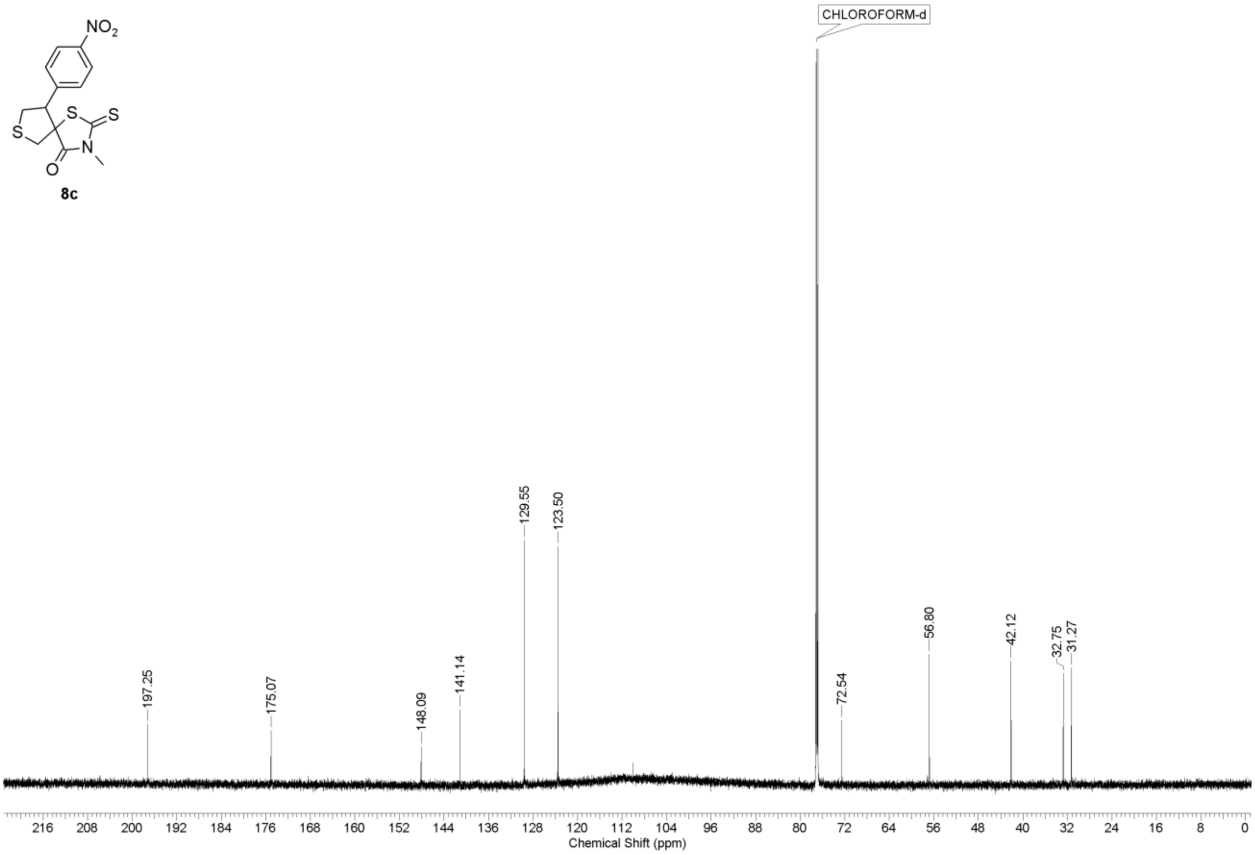
8c
CHLOROFORM-d
Chemical Shift (ppm)
197.25 175.07 148.09 141.14 129.55 123.50 72.54 56.80 42.12 32.75 31.27

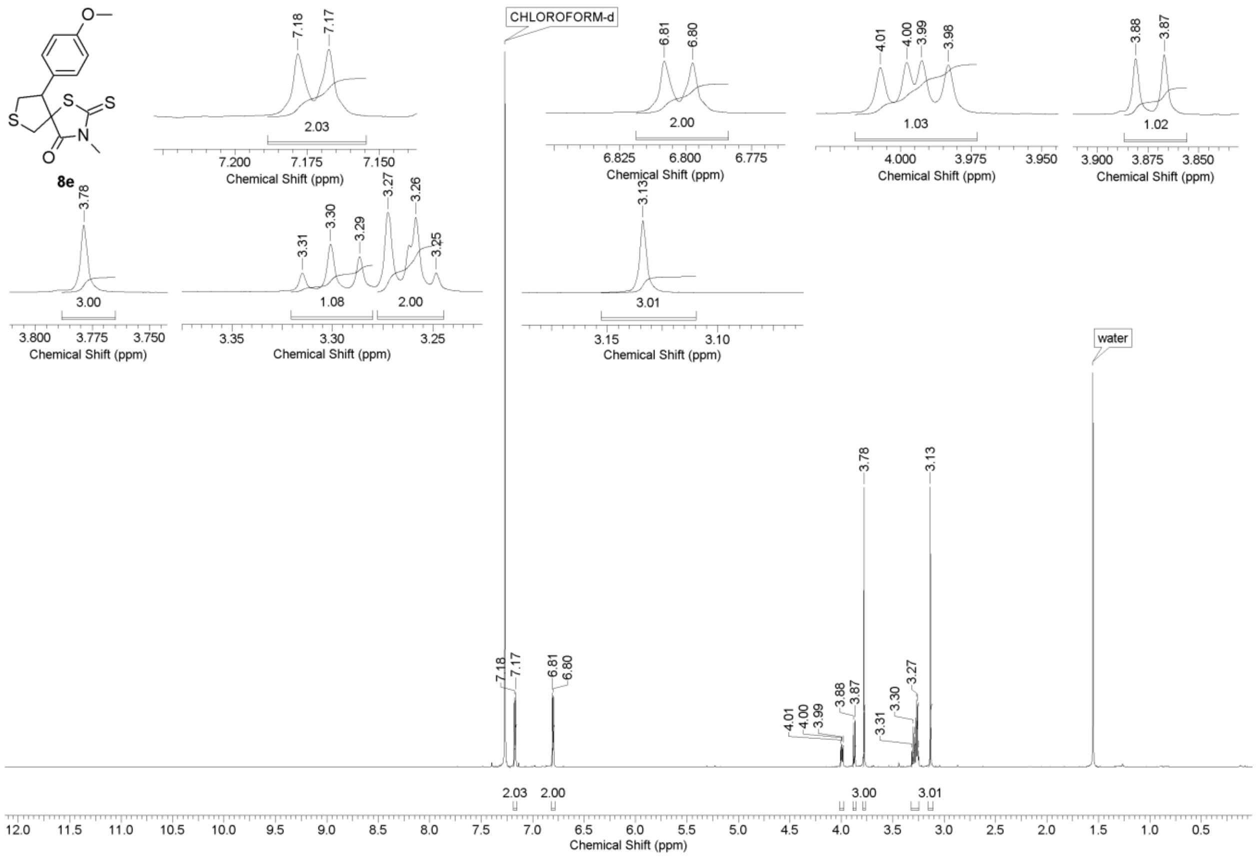
8e
CHLOROFORM-d
water
Chemical Shift (ppm)
7.18
7.17
6.81
6.80
4.01
4.00
3.99
3.98
3.88
3.87
3.78
3.31
3.30
3.29
3.27
3.26
3.25
3.13
2.03
2.00
1.03
1.02
3.00
1.08
3.01

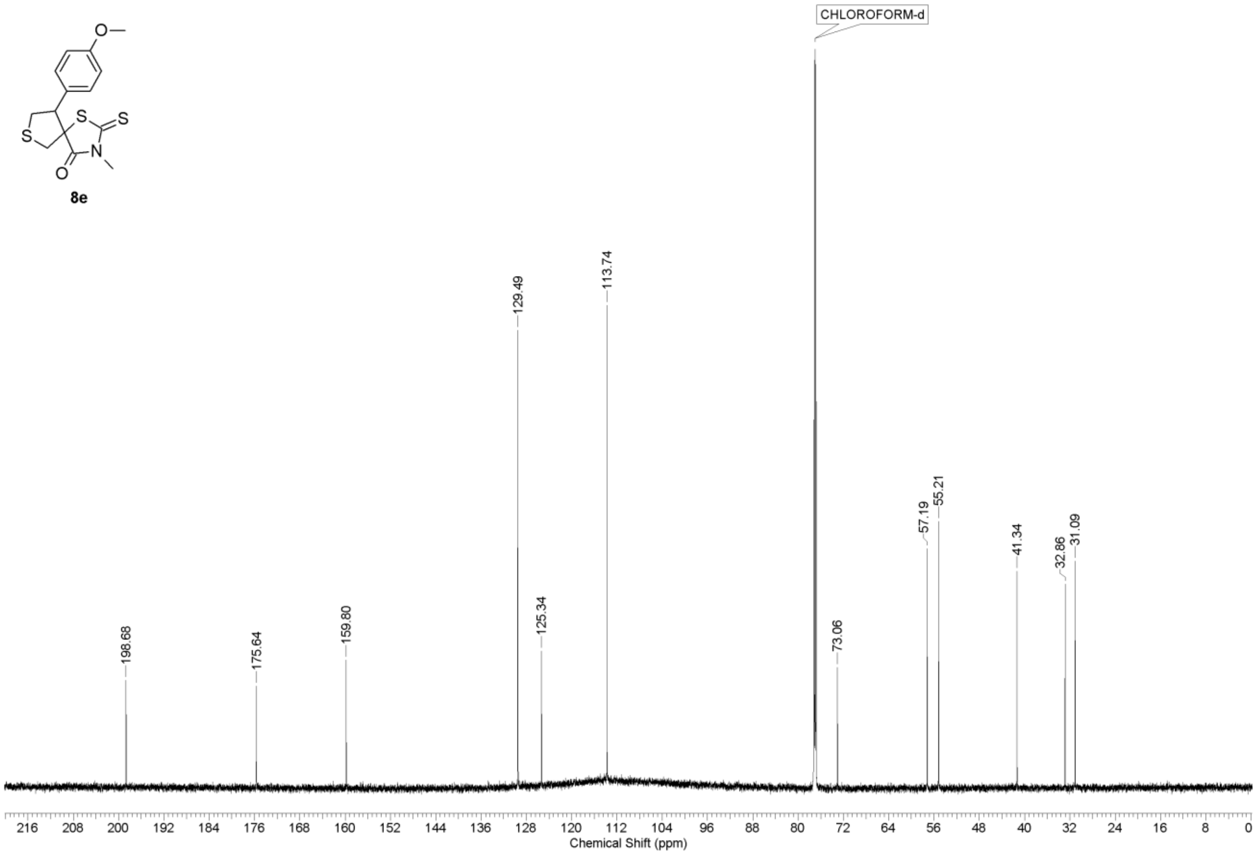
8e
CHLOROFORM-d
Chemical Shift (ppm)
198.68
175.64
159.80
129.49
125.34
113.74
73.06
57.19
55.21
41.34
32.86
31.09

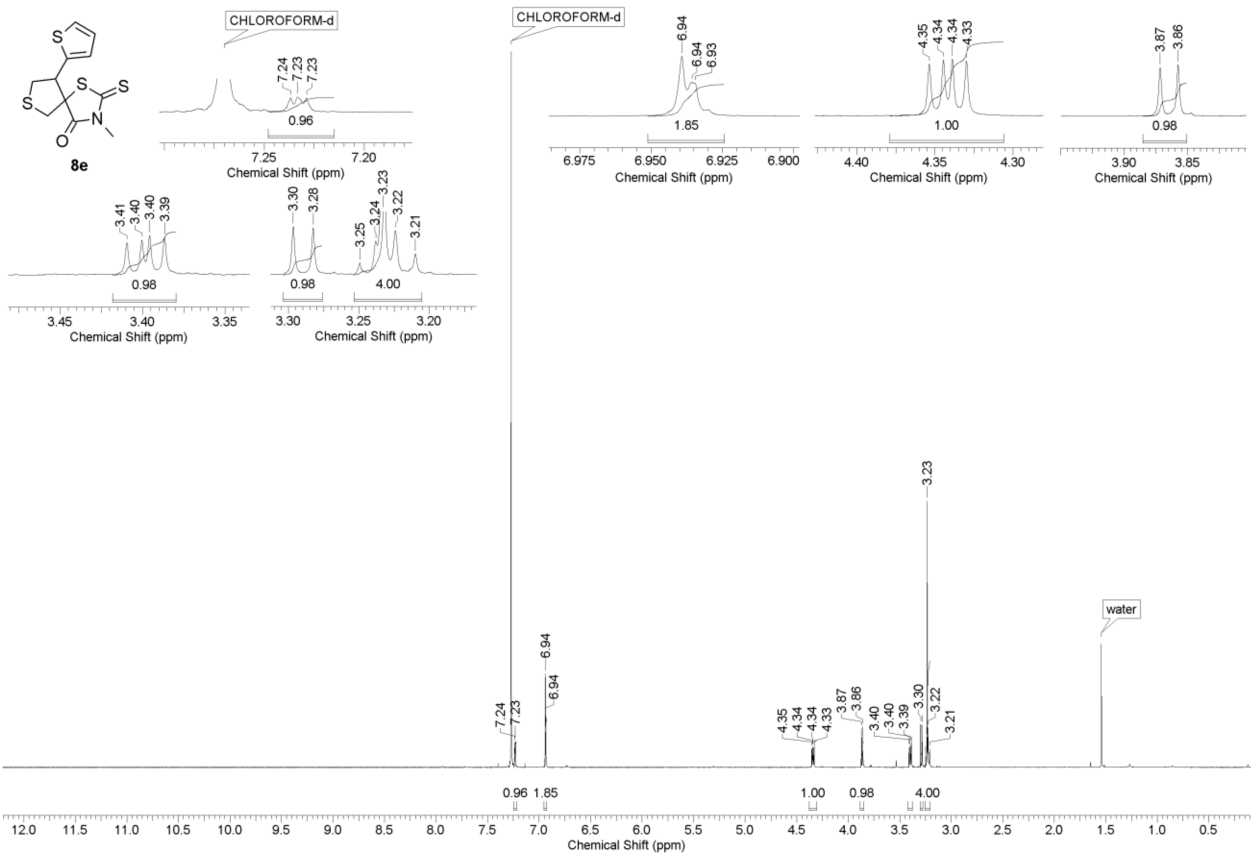
CHLOROFORM-d
Chemical Shift (ppm)
water
7.24
7.23
6.94
6.94
4.35
4.34
4.34
4.33
3.87
3.86
3.40
3.40
3.39
3.30
3.23
3.22
3.21
0.96
1.85
1.00
0.98
4.00

8e

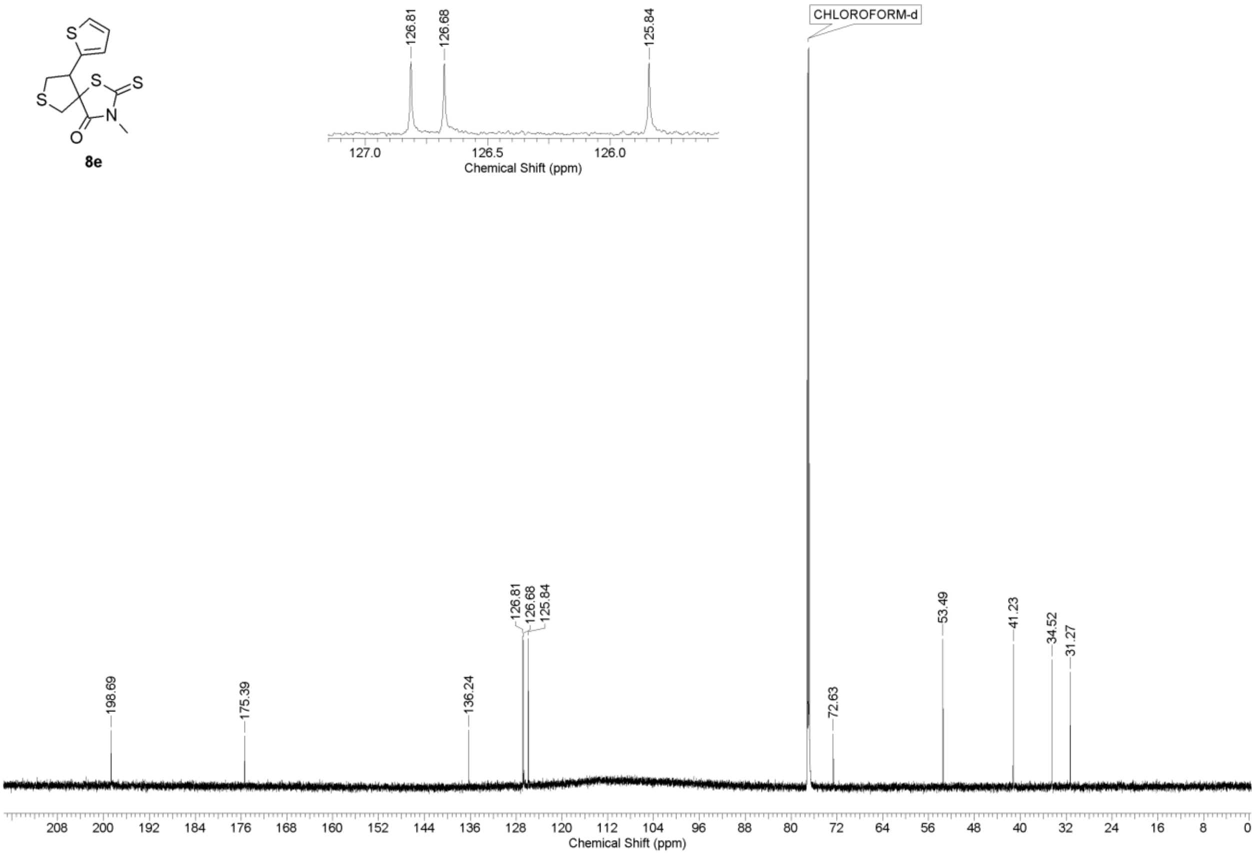
CHLOROFORM-d
Chemical Shift (ppm)
198.69
175.39
136.24
126.81
126.68
125.84
72.63
53.49
41.23
34.52
31.27

8e

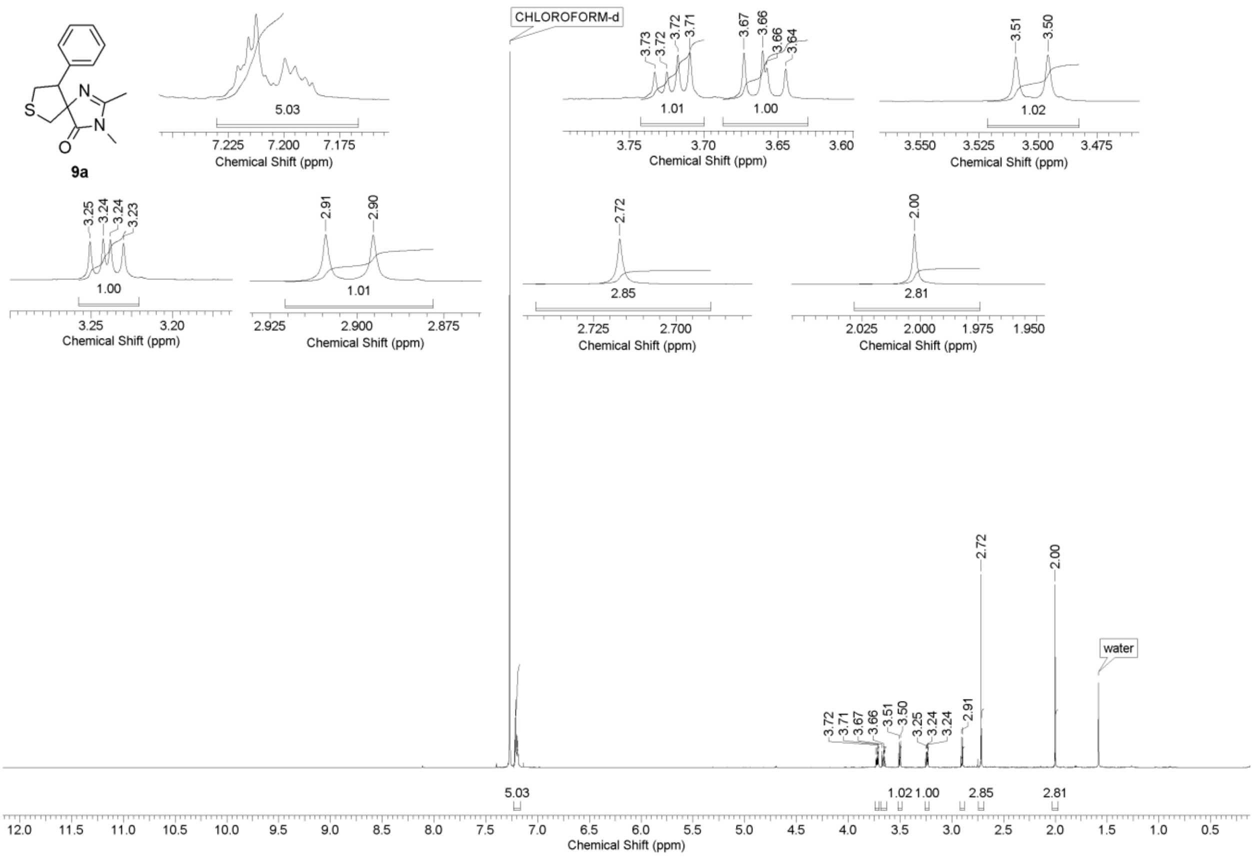
CHLOROFORM-d
water
5.03
1.01
1.00
1.02
2.85
2.81
2.72
2.00
3.72 3.71 3.67 3.66 3.51 3.50 3.25 3.24 3.24 2.91
Chemical Shift (ppm)

9a

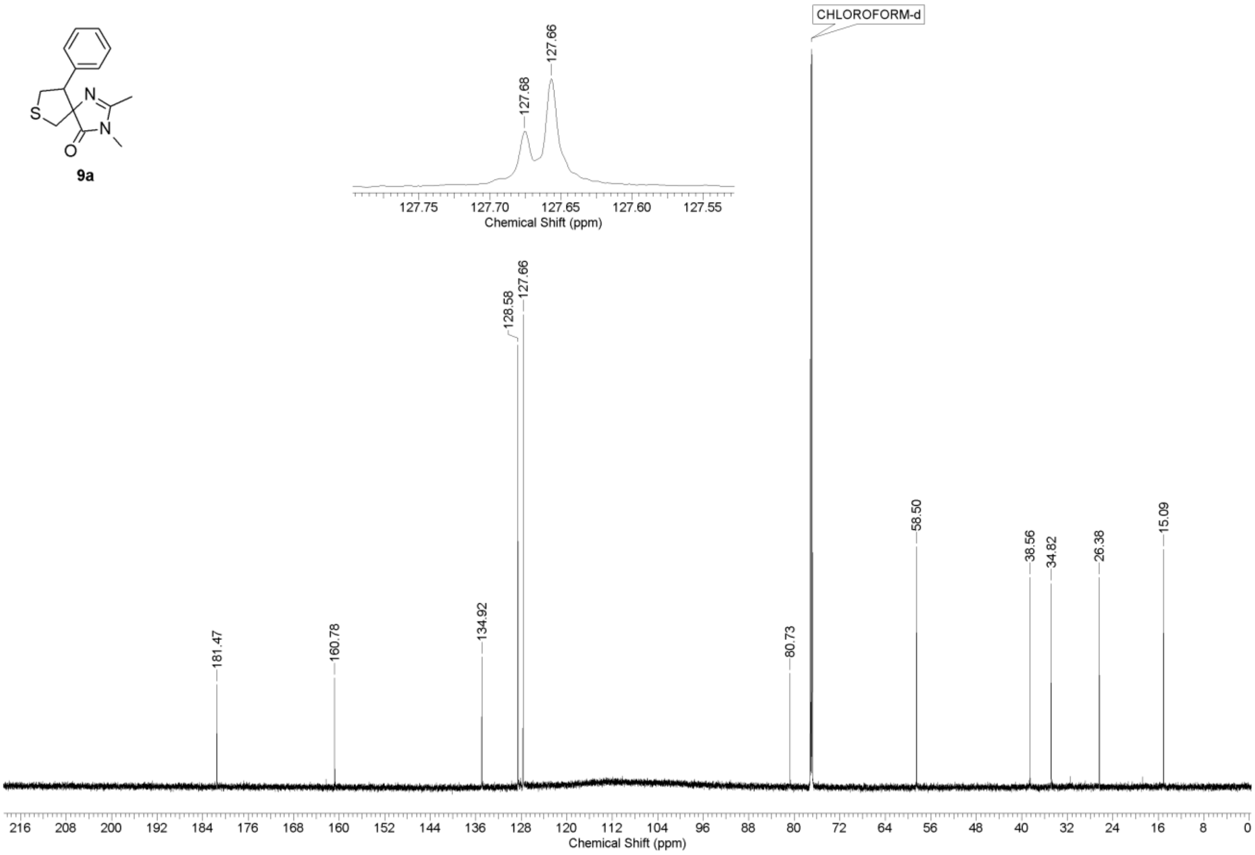
CHLOROFORM-d
181.47
160.78
134.92
128.58
127.66
127.68
80.73
58.50
38.56
34.82
26.38
15.09
Chemical Shift (ppm)

9a

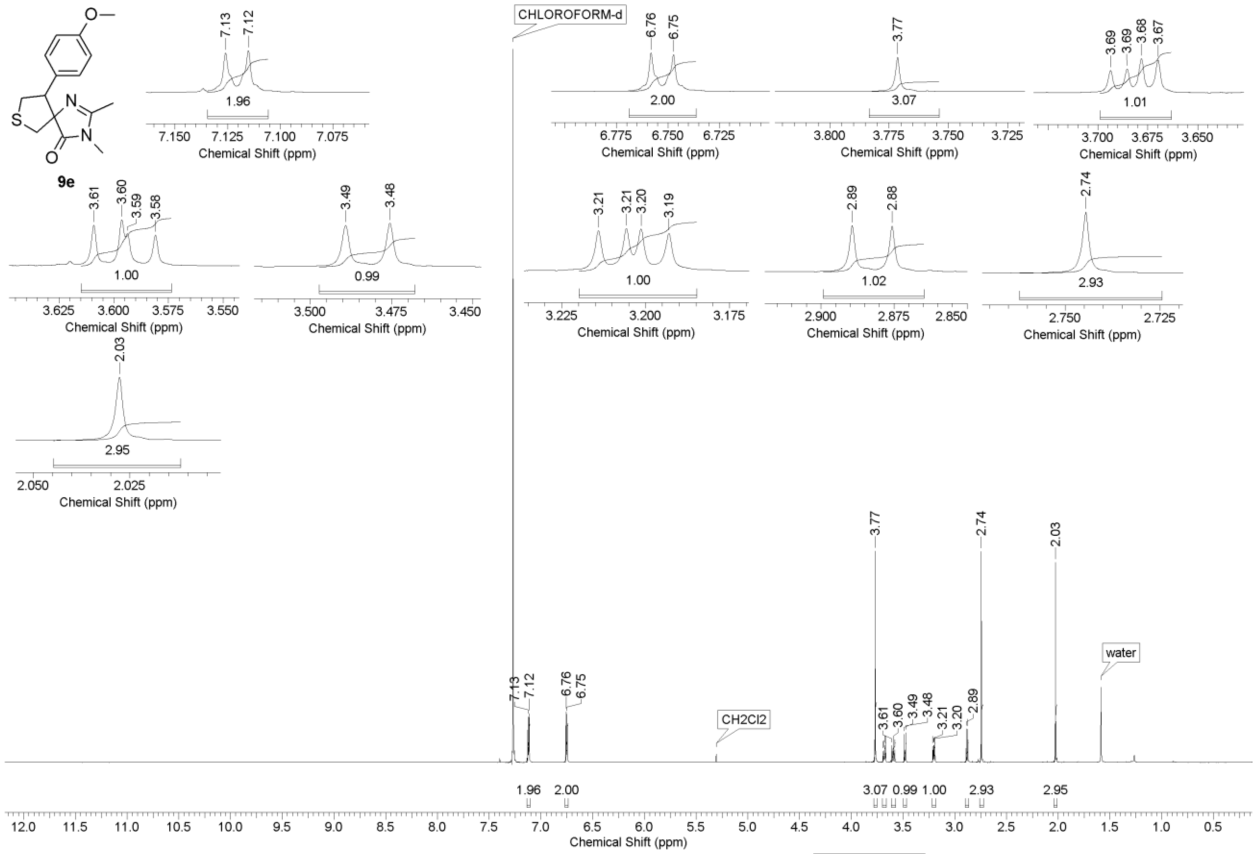

9e
CHLOROFORM-d
CH2Cl2
water
Chemical Shift (ppm)

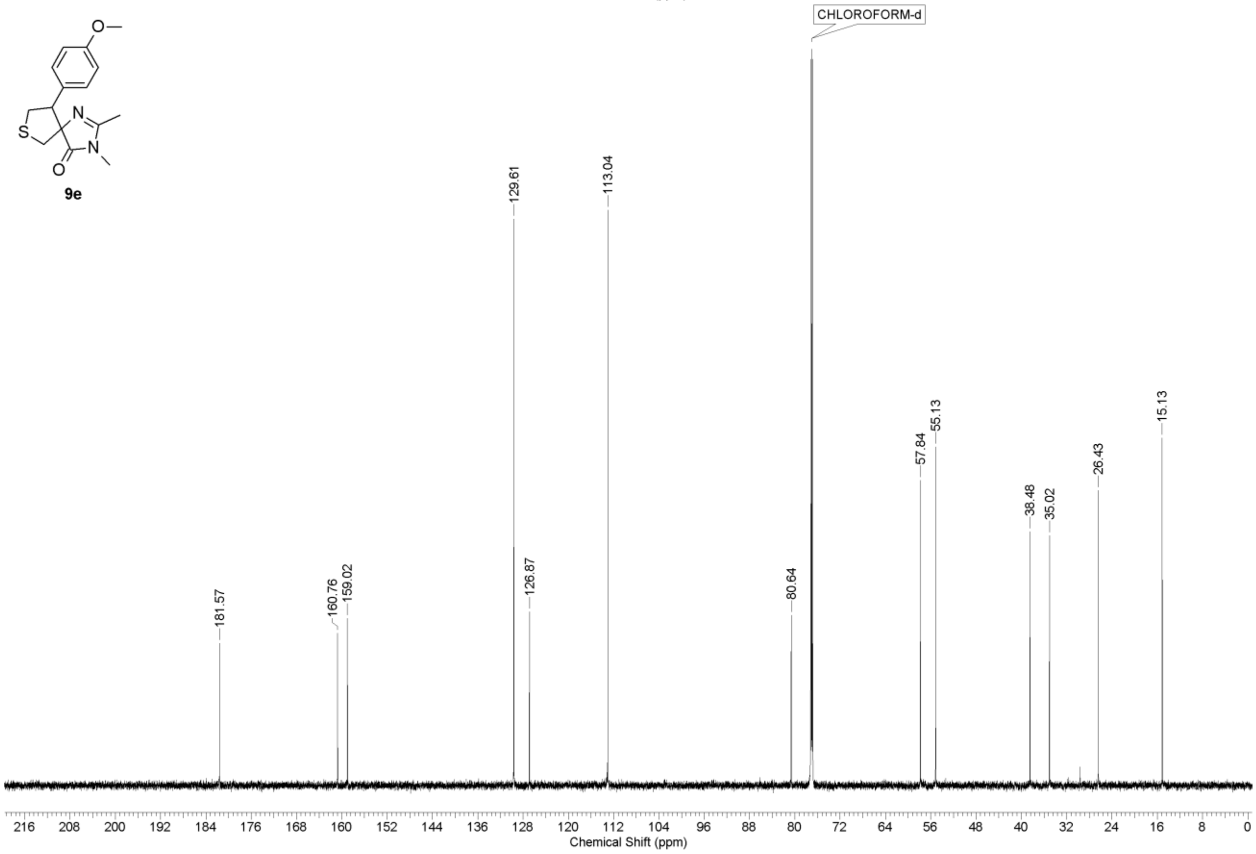

9e
CHLOROFORM-d
181.57
160.76
159.02
129.61
126.87
113.04
80.64
57.84
55.13
38.48
35.02
26.43
15.13
Chemical Shift (ppm)

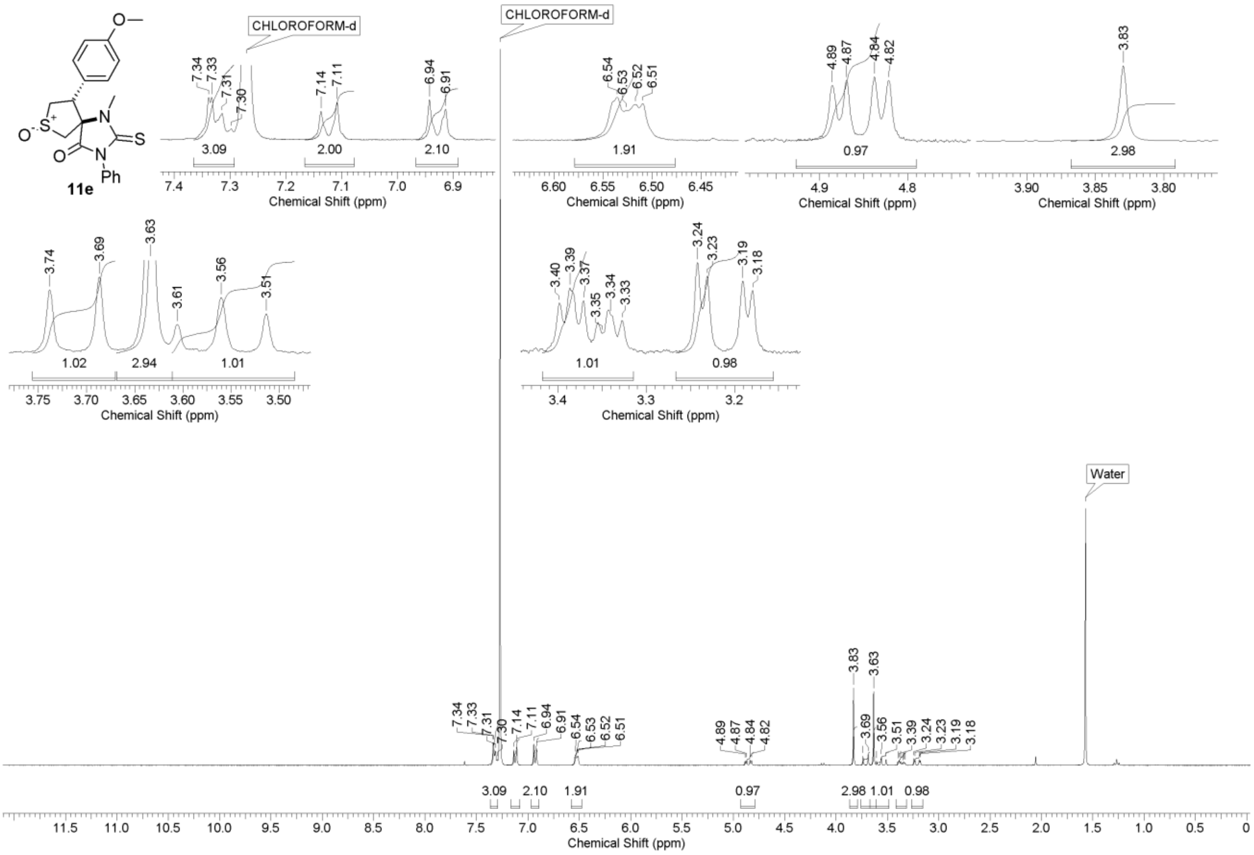
11e
CHLOROFORM-d
CHLOROFORM-d
Water
Chemical Shift (ppm)

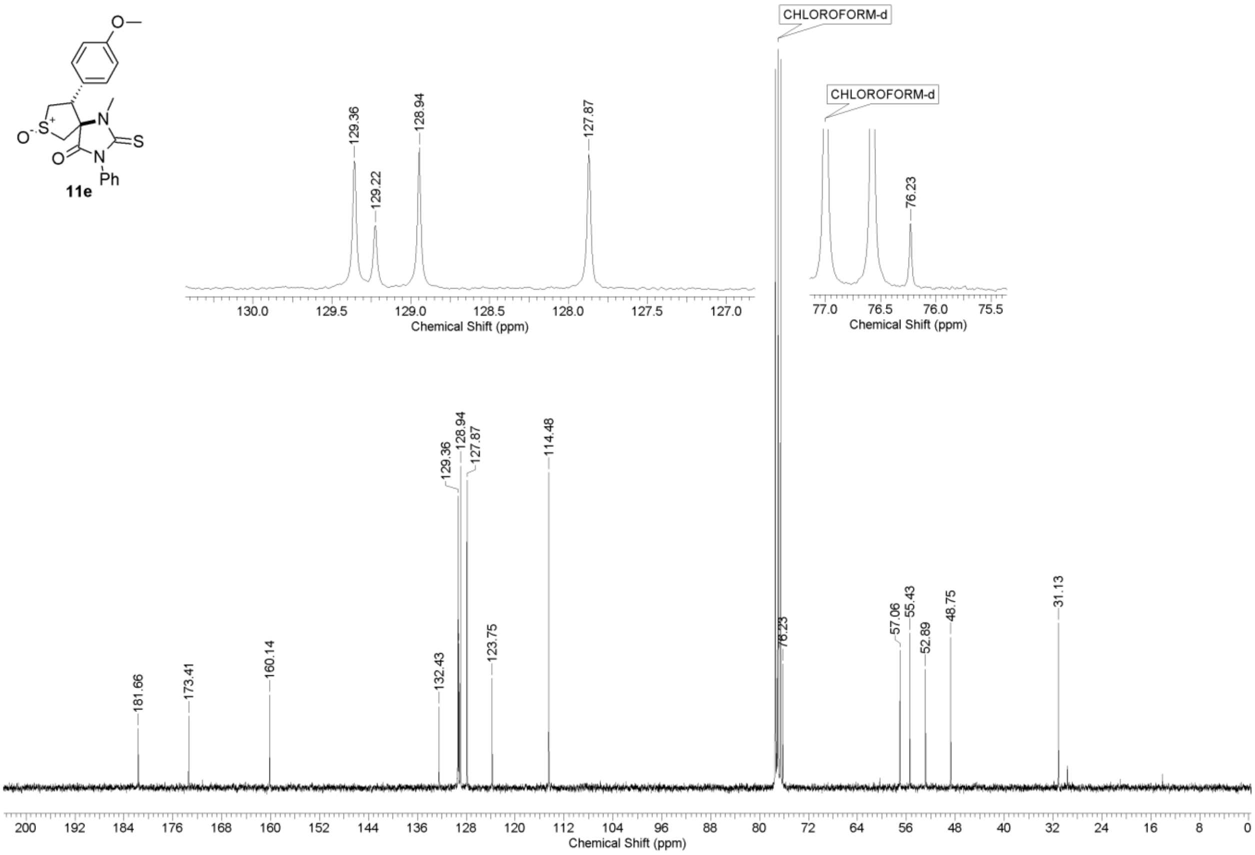
11e
CHLOROFORM-d
CHLOROFORM-d
Chemical Shift (ppm)

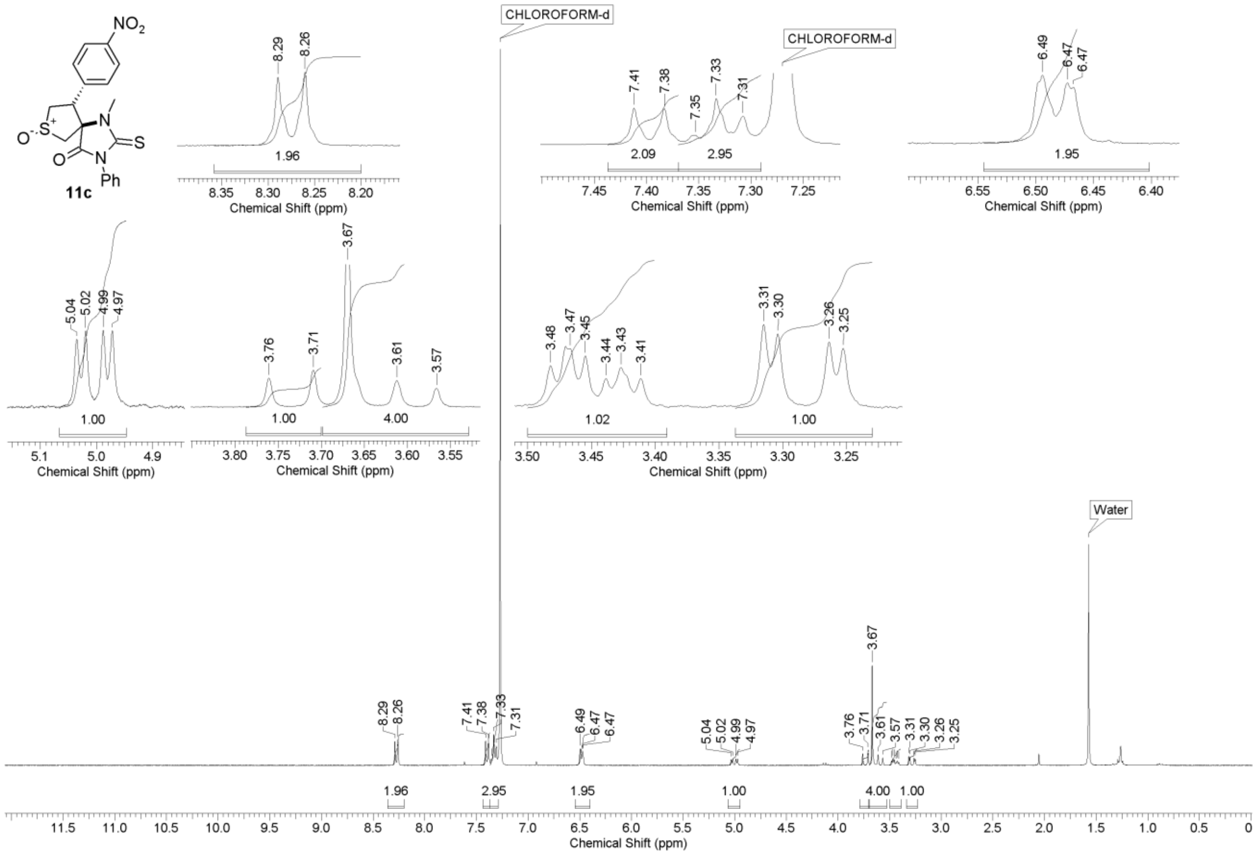
NO2
O-S+
N
S
O
N
Ph
11c
CHLOROFORM-d
Water
Chemical Shift (ppm)

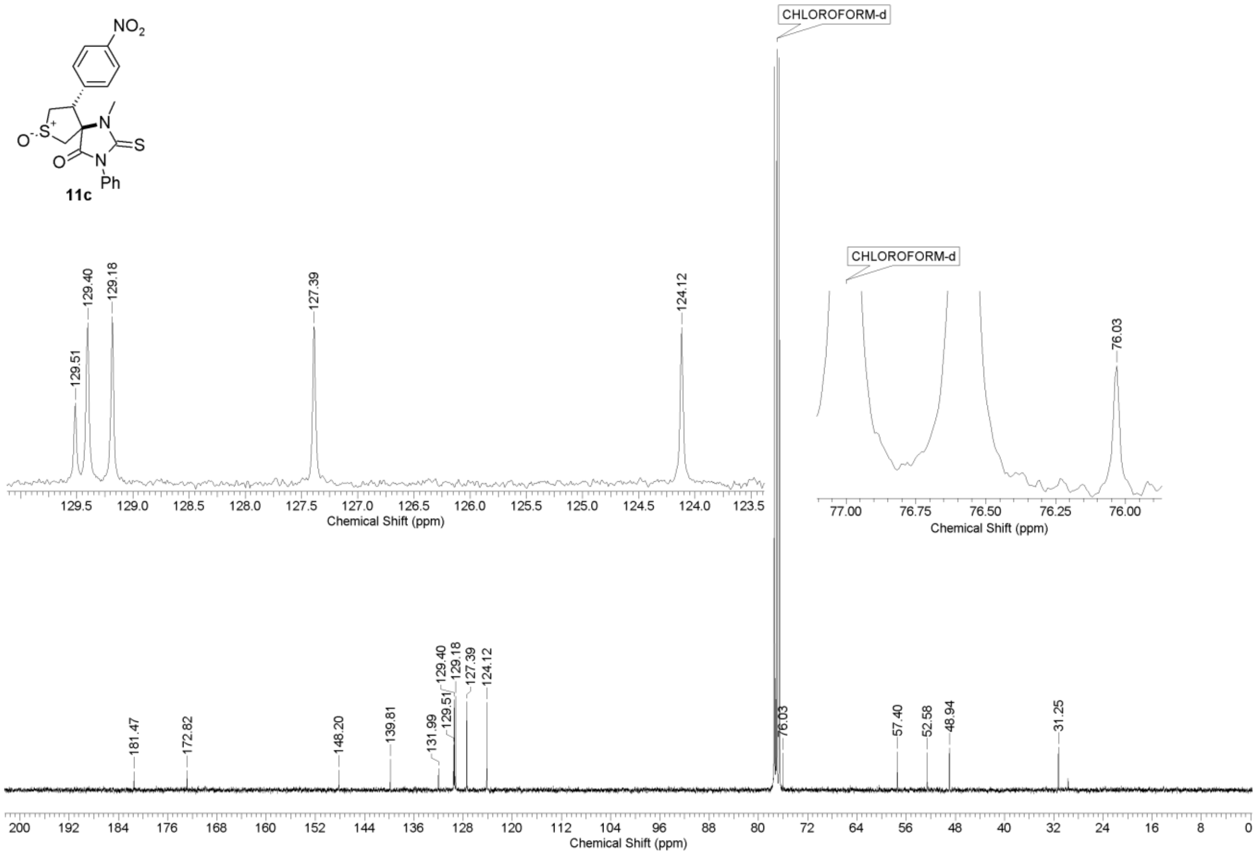
NO2
O-S+
N
S
O
N
Ph
11c
CHLOROFORM-d
Chemical Shift (ppm)

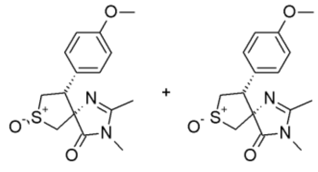

13e
*minor isomer

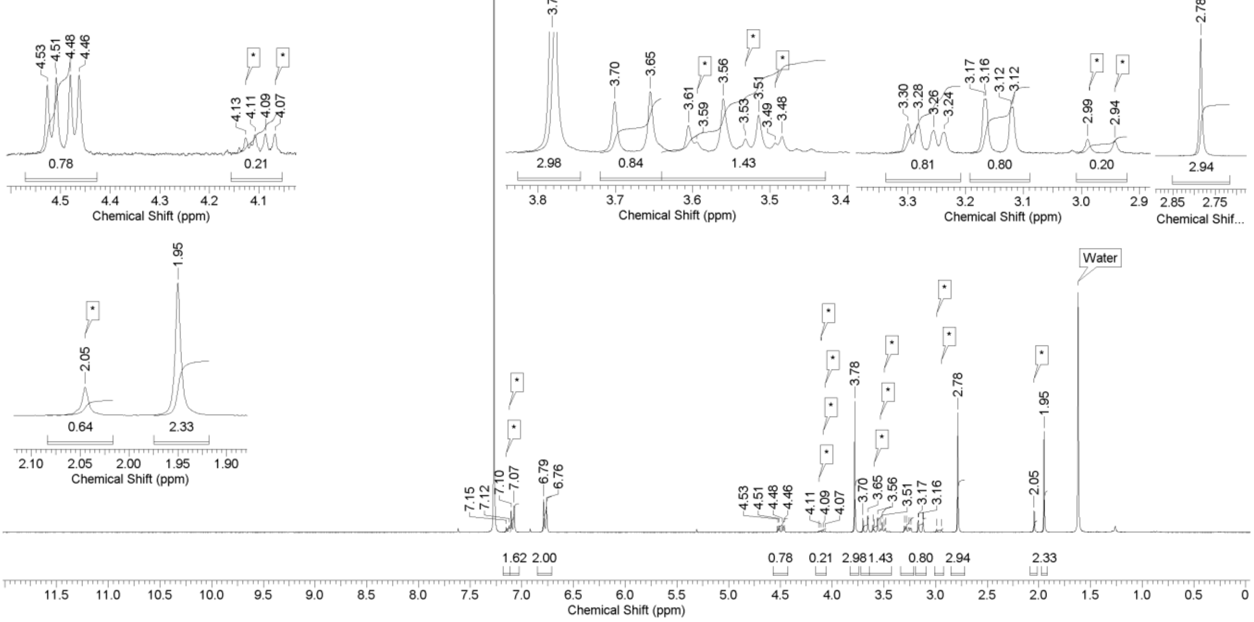

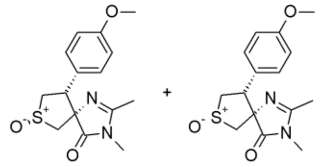

13e
*minor isomer

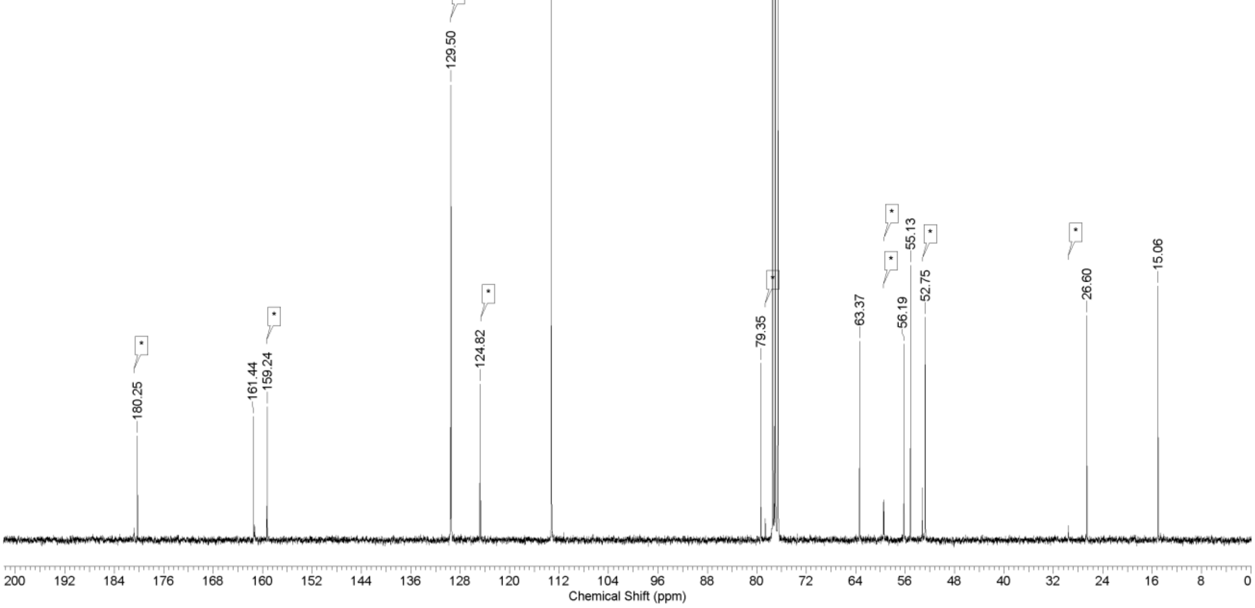

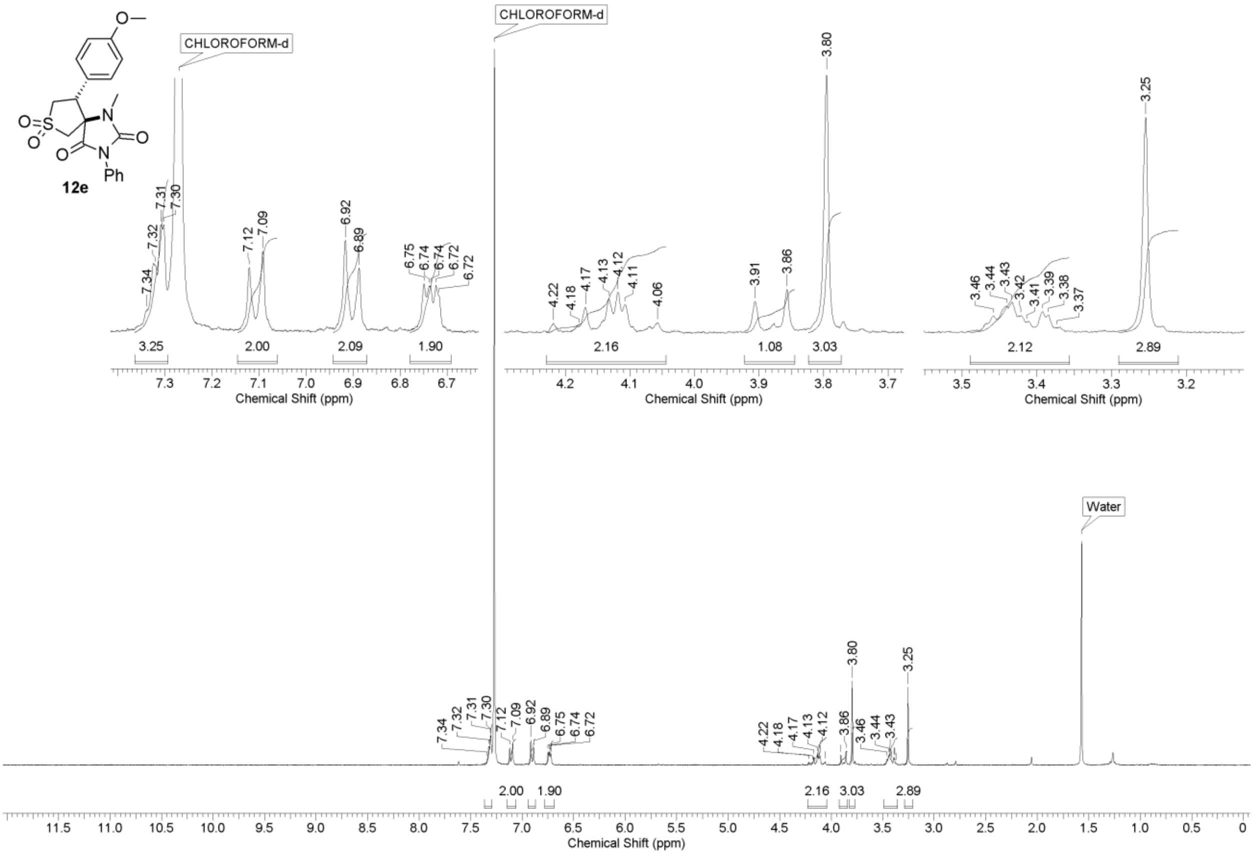
12e
CHLOROFORM-d
CHLOROFORM-d
Water
Chemical Shift (ppm)

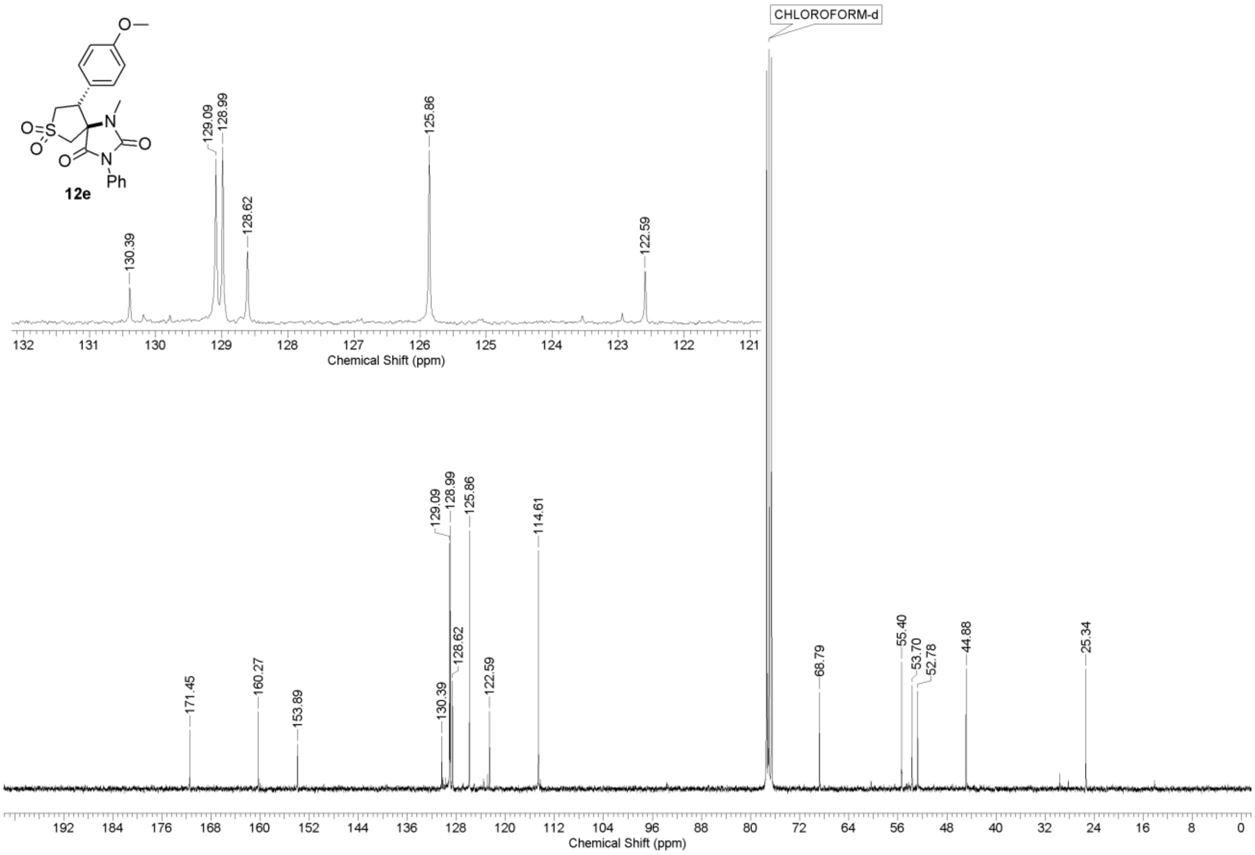
12e
CHLOROFORM-d
Chemical Shift (ppm)

8. Copies of mass-spectra

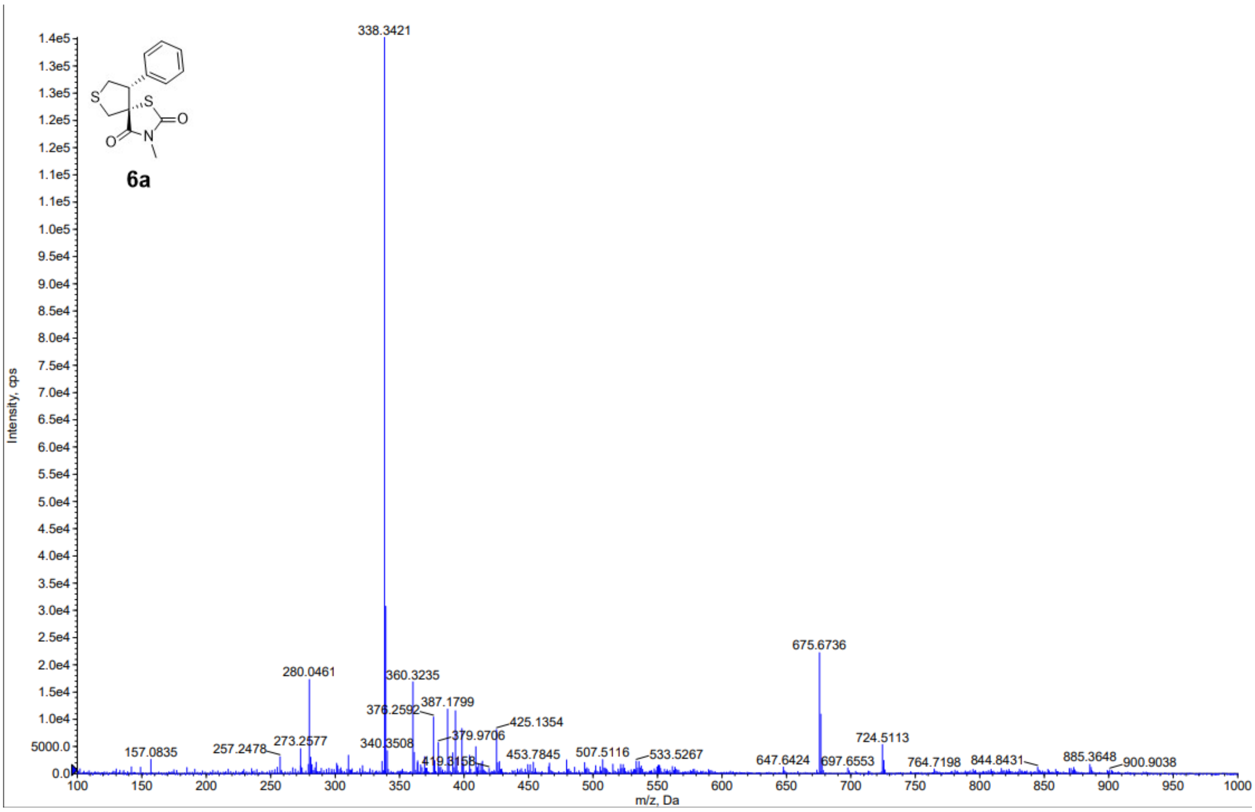

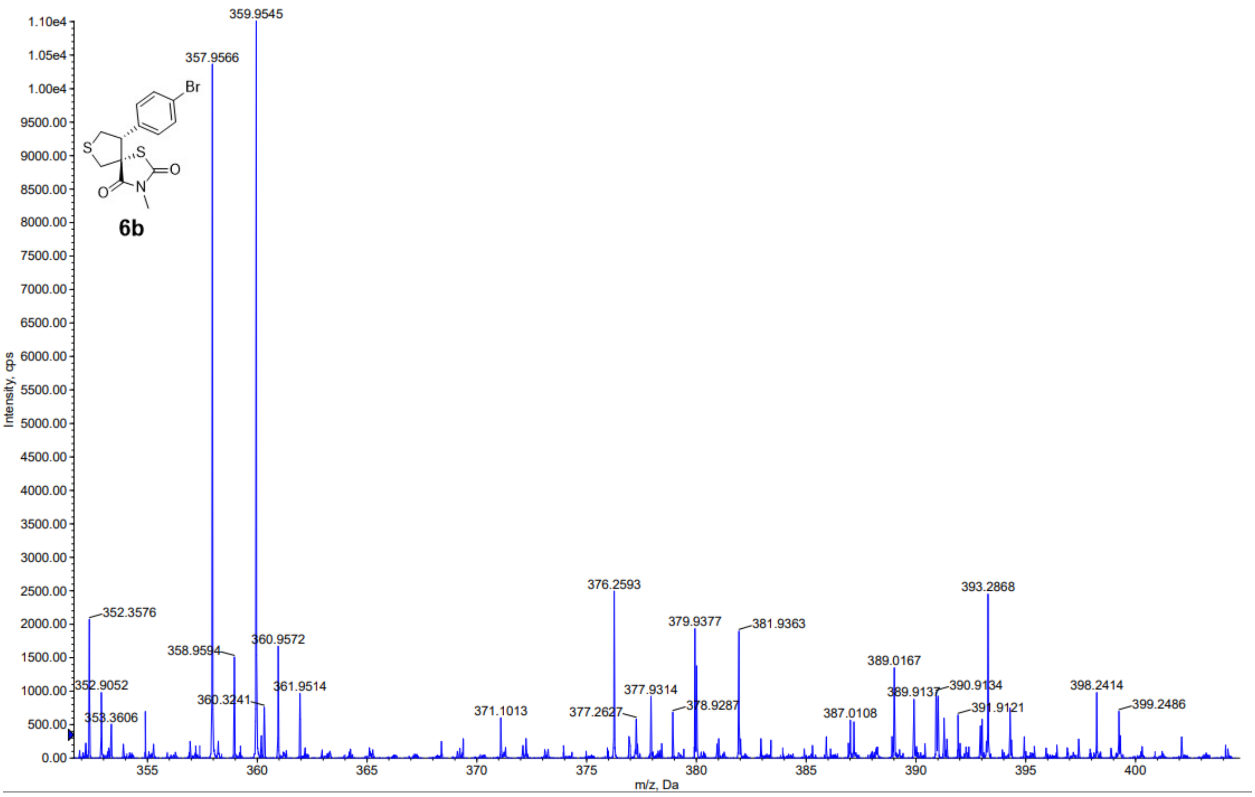

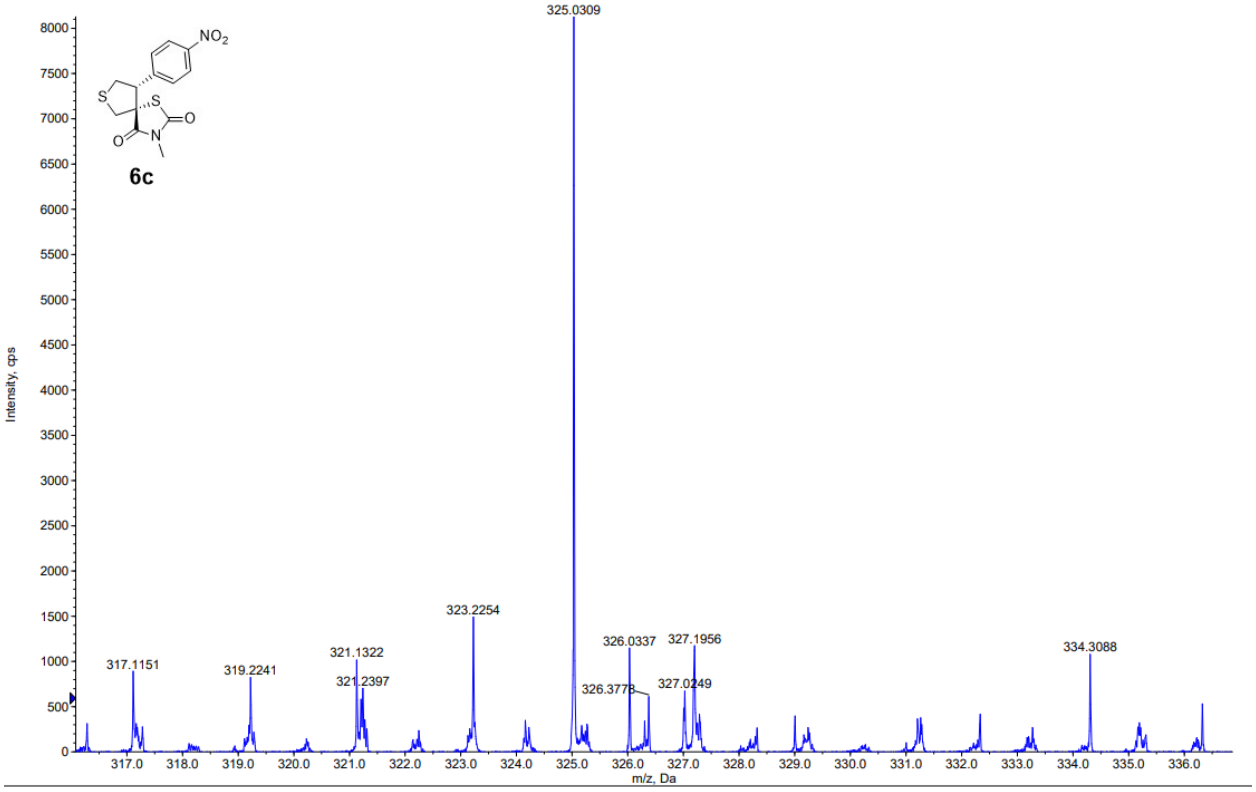

NO_2
S
S
O
O
N
6c
Intensity, cps
m/z, Da
325.0309
323.2254
321.1322
321.2397
317.1151
319.2241
326.0337
327.1956
326.3778
327.0249
334.3088

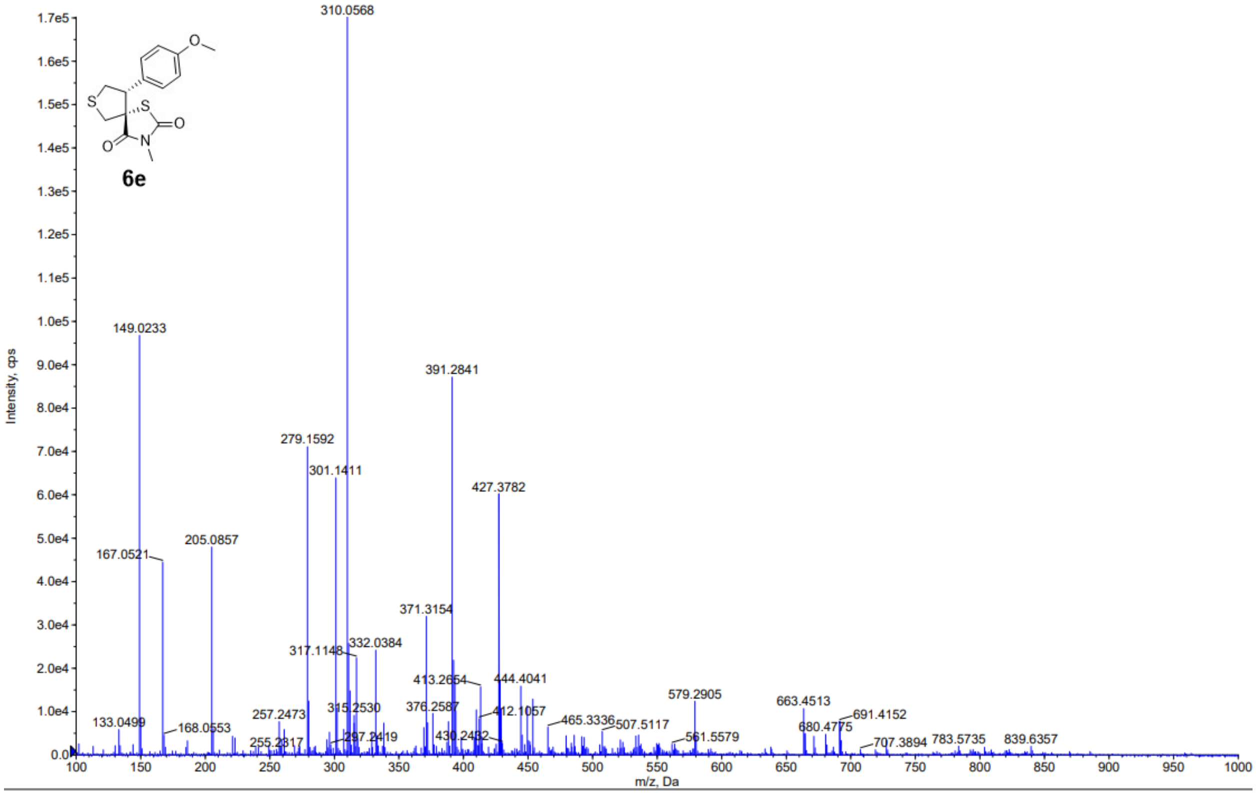

O
S
S
O
O
N
6e
Intensity, cps
m/z, Da
310.0568
149.0233
391.2841
279.1592
301.1411
427.3782
167.0521
205.0857
371.3154
317.1148
332.0384
413.2654
444.4041
579.2905
663.4513
691.4152
133.0499
168.0553
257.2473
315.2530
376.2587
412.1057
465.3336
507.5117
680.4775
255.2317
297.2419
430.2452
561.5579
707.3894
783.5735
839.6357

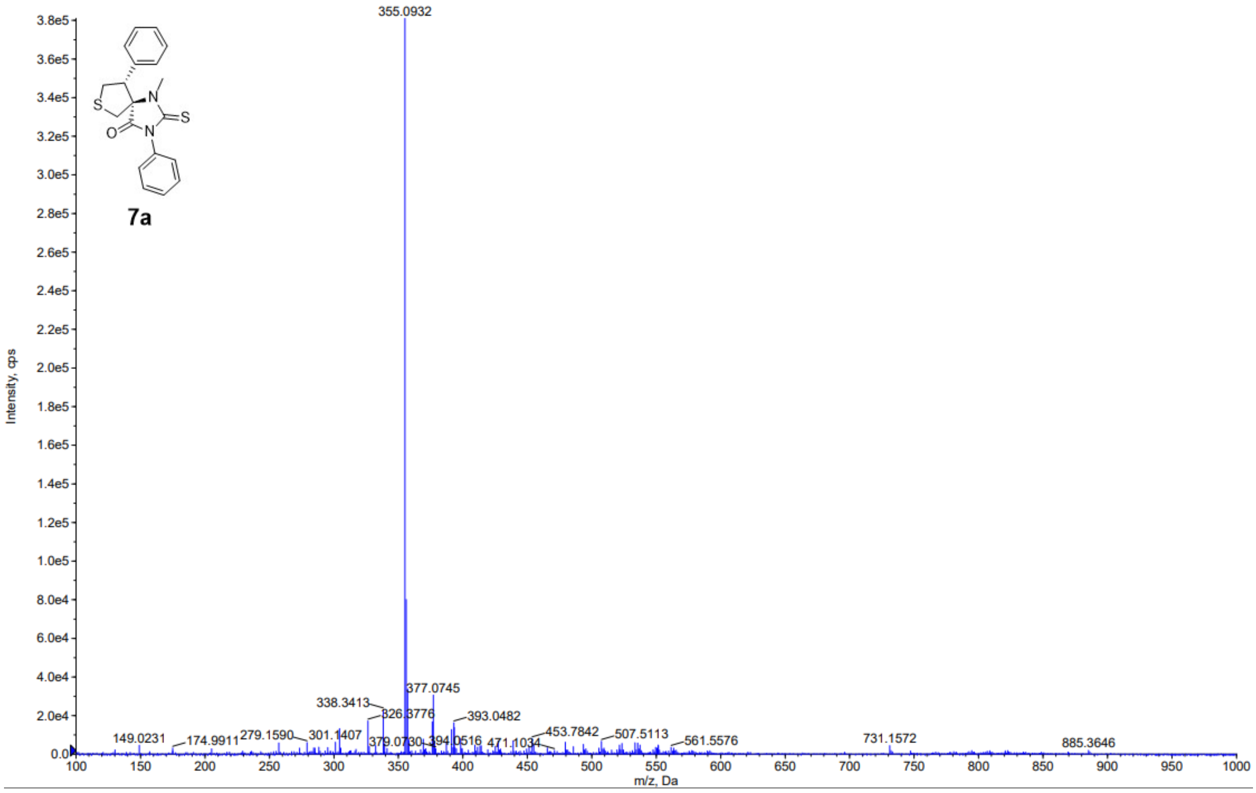
7a
355.0932
377.0745
338.3413
326.3776
393.0482
149.0231
174.9911
279.1590
301.1407
379.0730
394.0516
471.1034
453.7842
507.5113
561.5576
731.1572
885.3646
Intensity, cps
m/z, Da

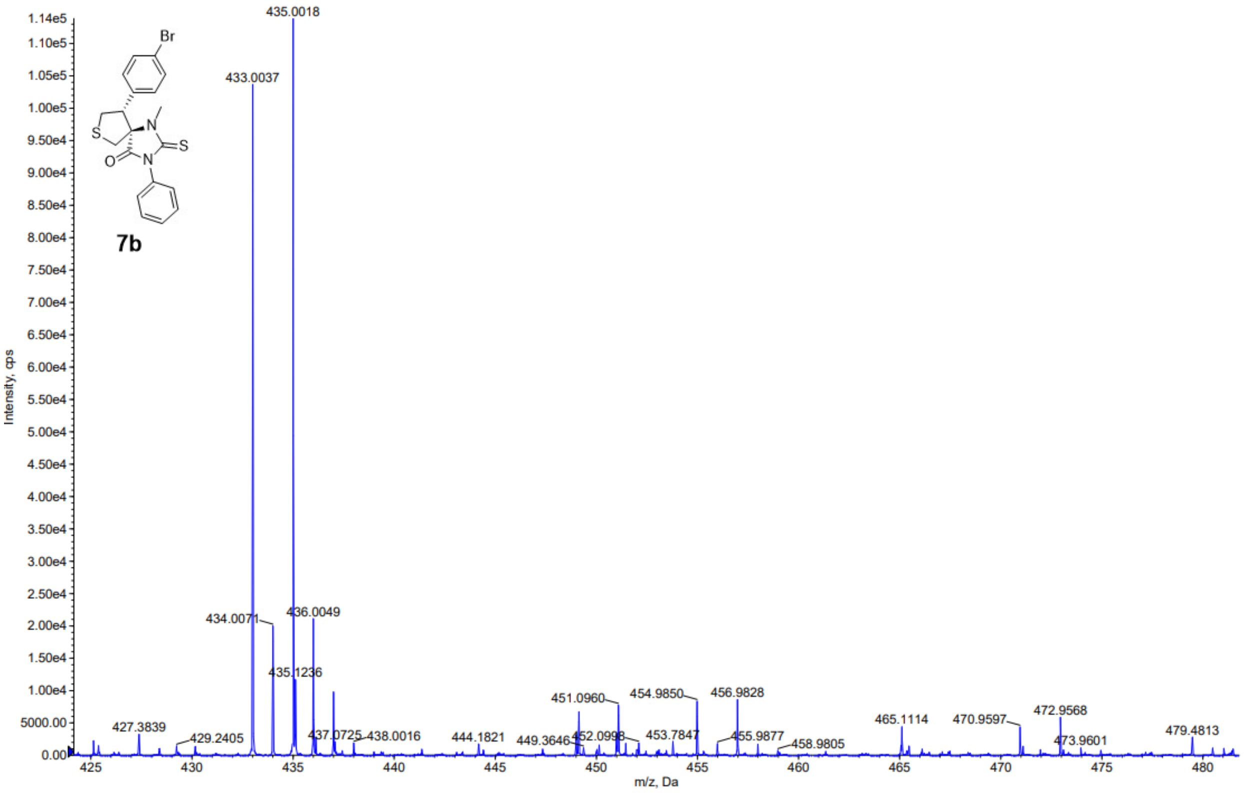
Br
7b
435.0018
433.0037
434.0071
436.0049
435.1236
427.3839
429.2405
437.0725
438.0016
444.1821
449.3646
451.0960
452.0998
453.7847
454.9850
455.9877
456.9828
458.9805
465.1114
470.9597
472.9568
473.9601
479.4813
Intensity, cps
m/z, Da

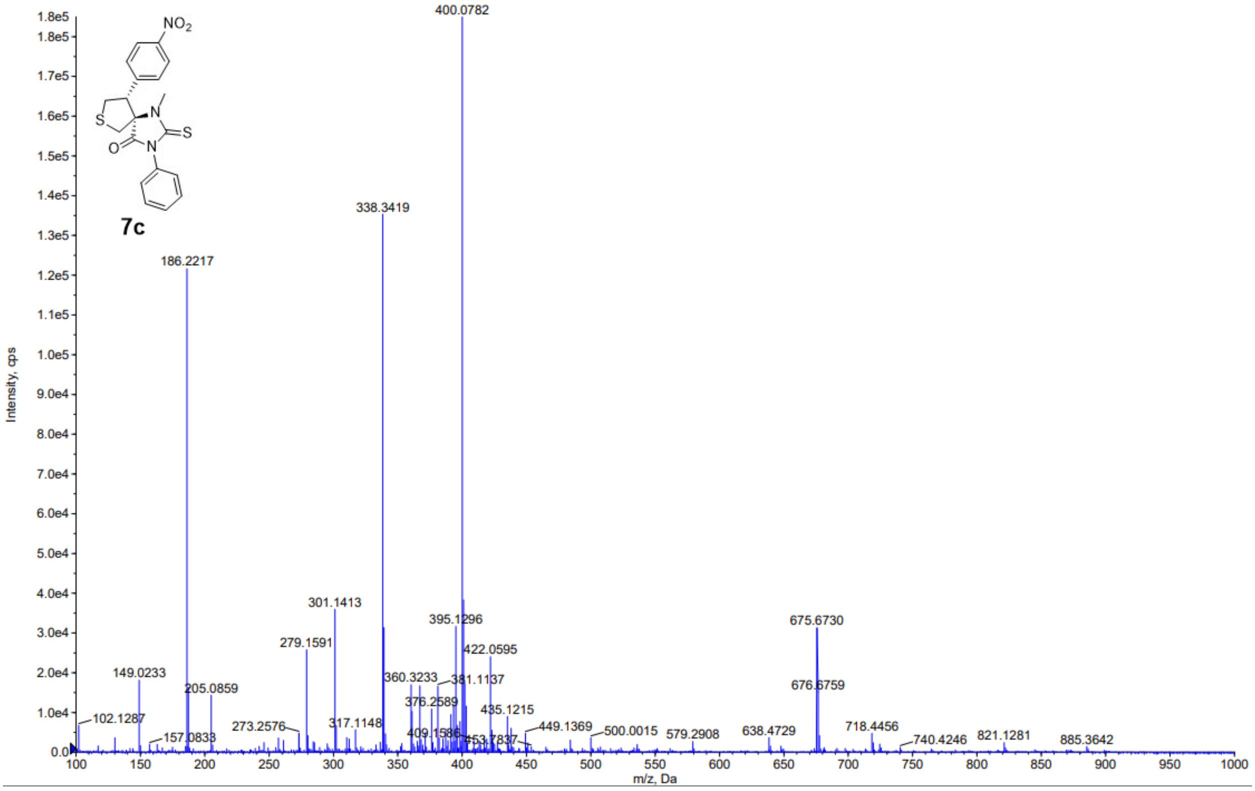
7c
NO_2
S
N
S
O
N
Intensity, cps
m/z, Da
400.0782
338.3419
186.2217
301.1413
395.1296
675.6730
279.1591
422.0595
149.0233
360.3233
381.1137
205.0859
676.6759
376.2589
435.1215
102.1287
449.1369
273.2576
317.1148
718.4456
500.0015
579.2908
638.4729
409.1586
821.1281
157.0833
453.7837
740.4246
885.3642

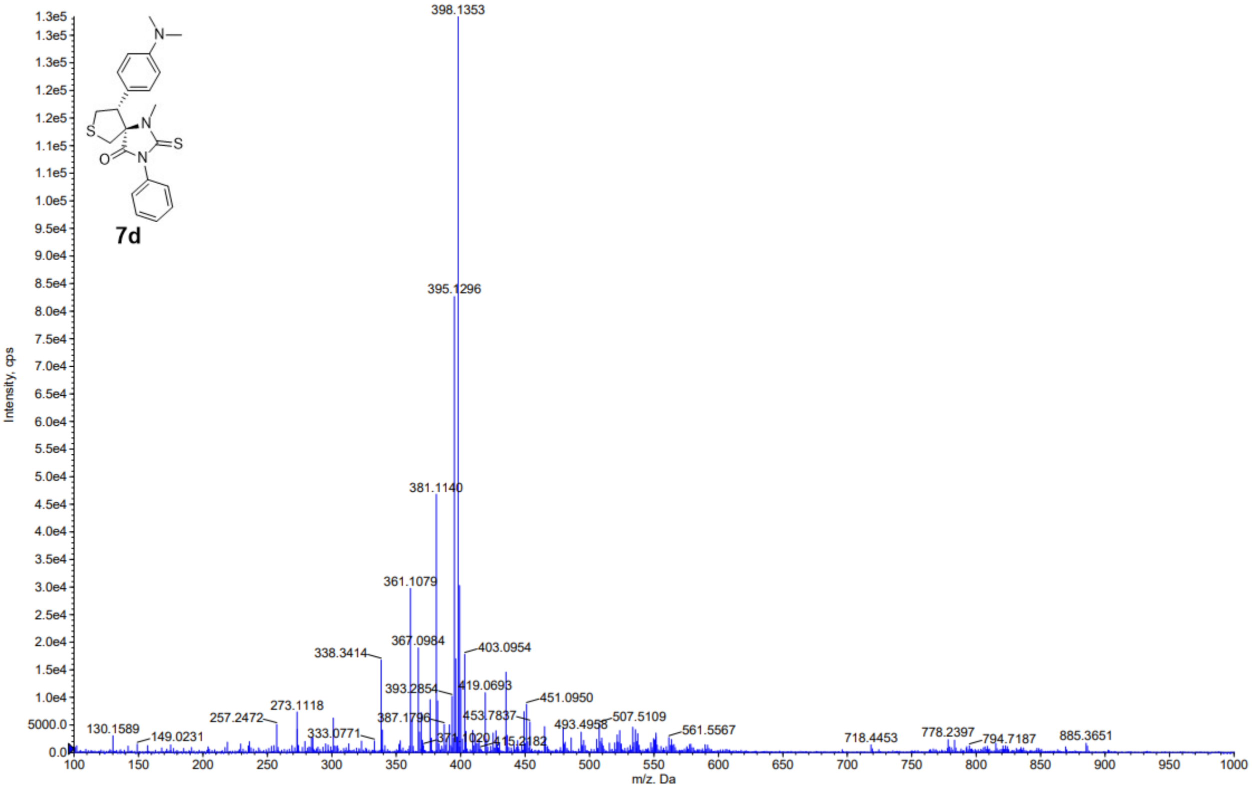
7d
N
S
N
S
O
N
Intensity, cps
m/z, Da
398.1353
395.1296
381.1140
361.1079
367.0984
338.3414
403.0954
393.2854
419.0693
273.1118
451.0950
257.2472
387.1796
453.7837
507.5109
493.4958
130.1589
333.0771
561.5567
149.0231
778.2397
885.3651
718.4453
794.7187

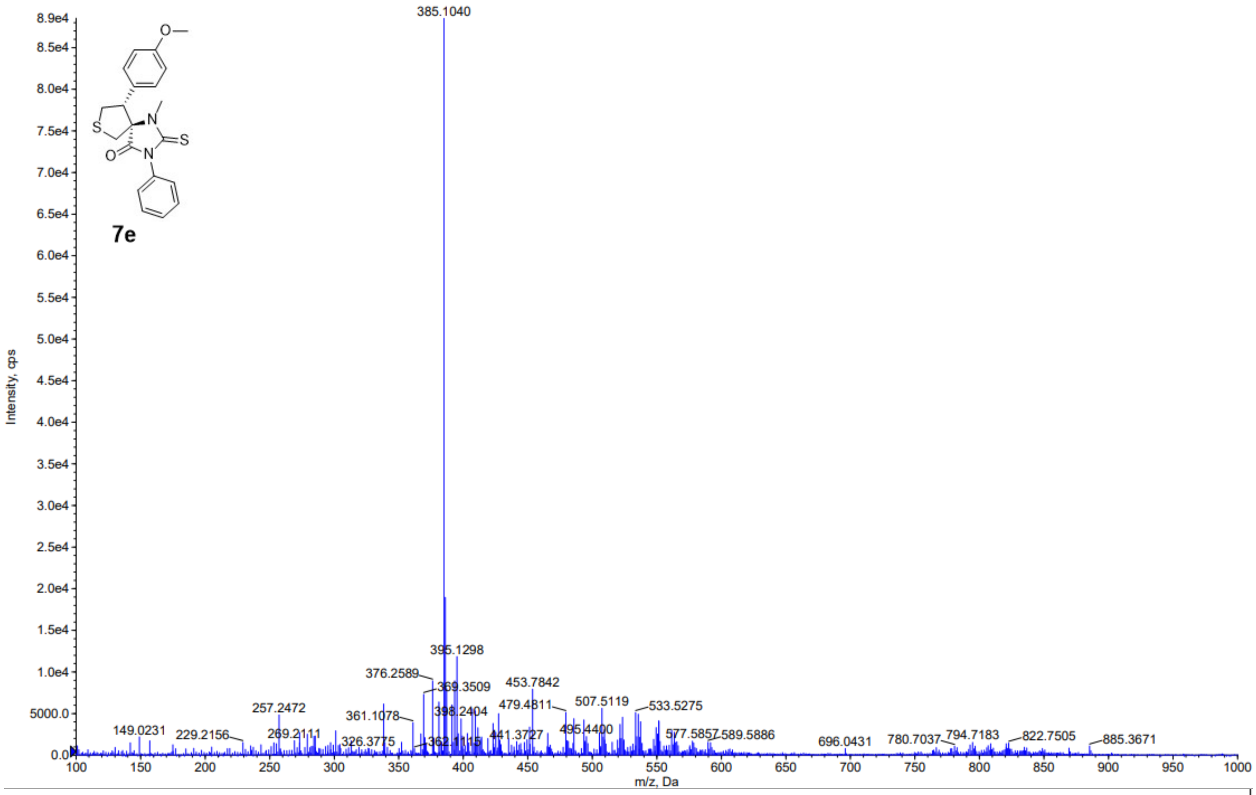
7e
385.1040
Intensity, cps
m/z, Da

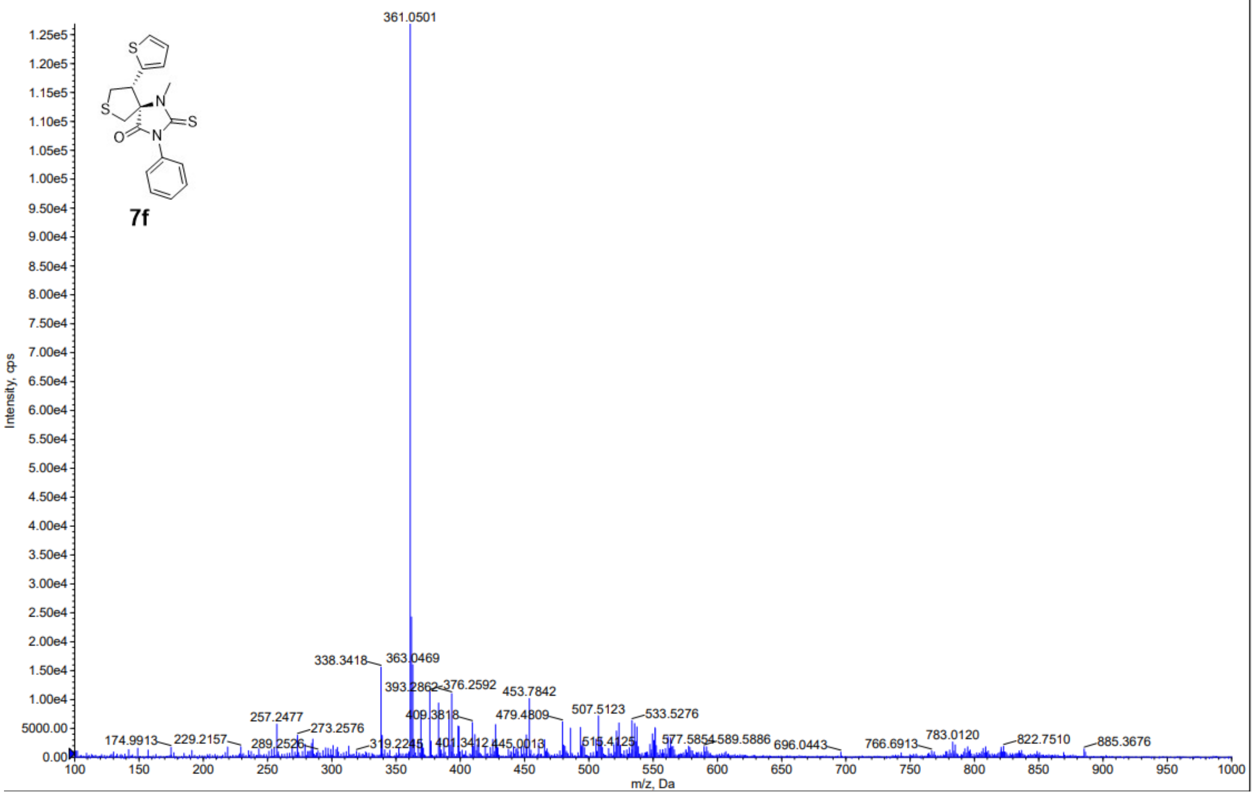
7f
361.0501
Intensity, cps
m/z, Da

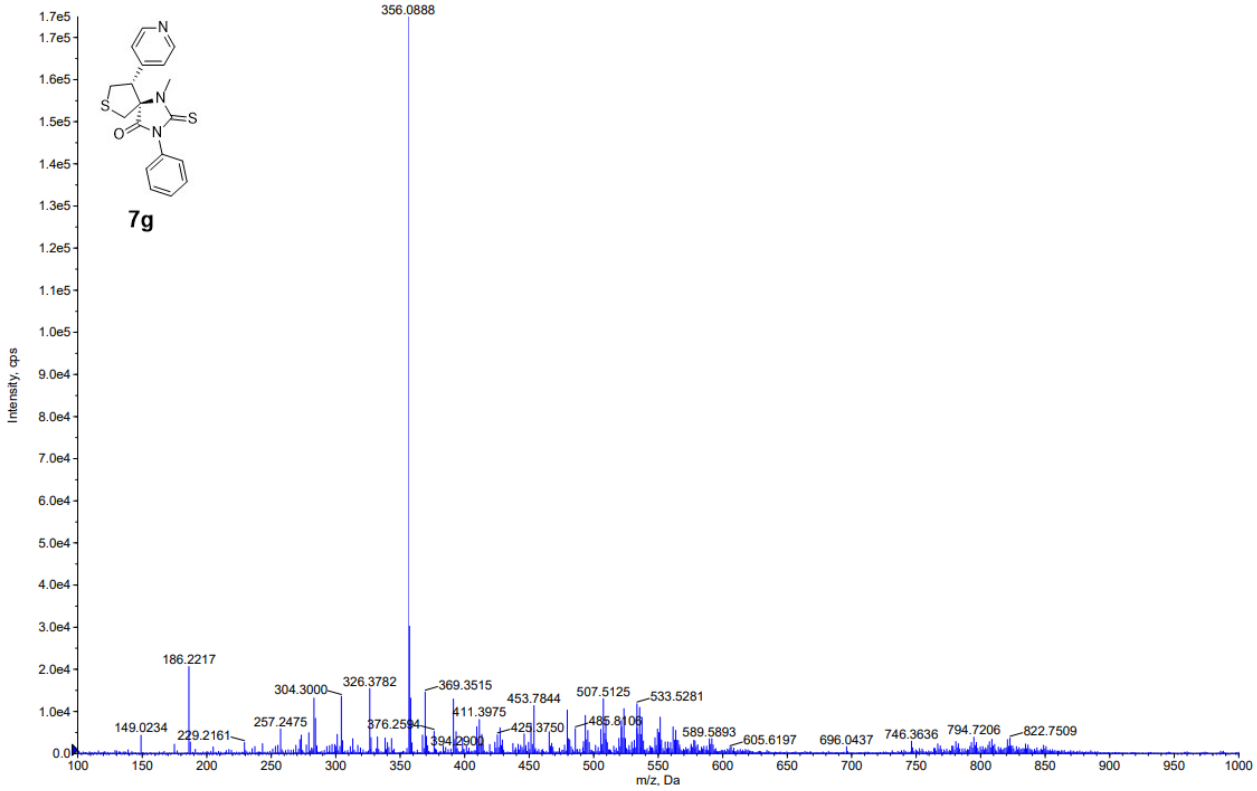
7g
356.0888
186.2217
304.3000
326.3782
369.3515
453.7844
507.5125
533.5281
149.0234
229.2161
257.2475
376.2594
411.3975
425.3750
485.8106
589.5893
605.6197
696.0437
746.3636
794.7206
822.7509
Intensity, cps
m/z, Da

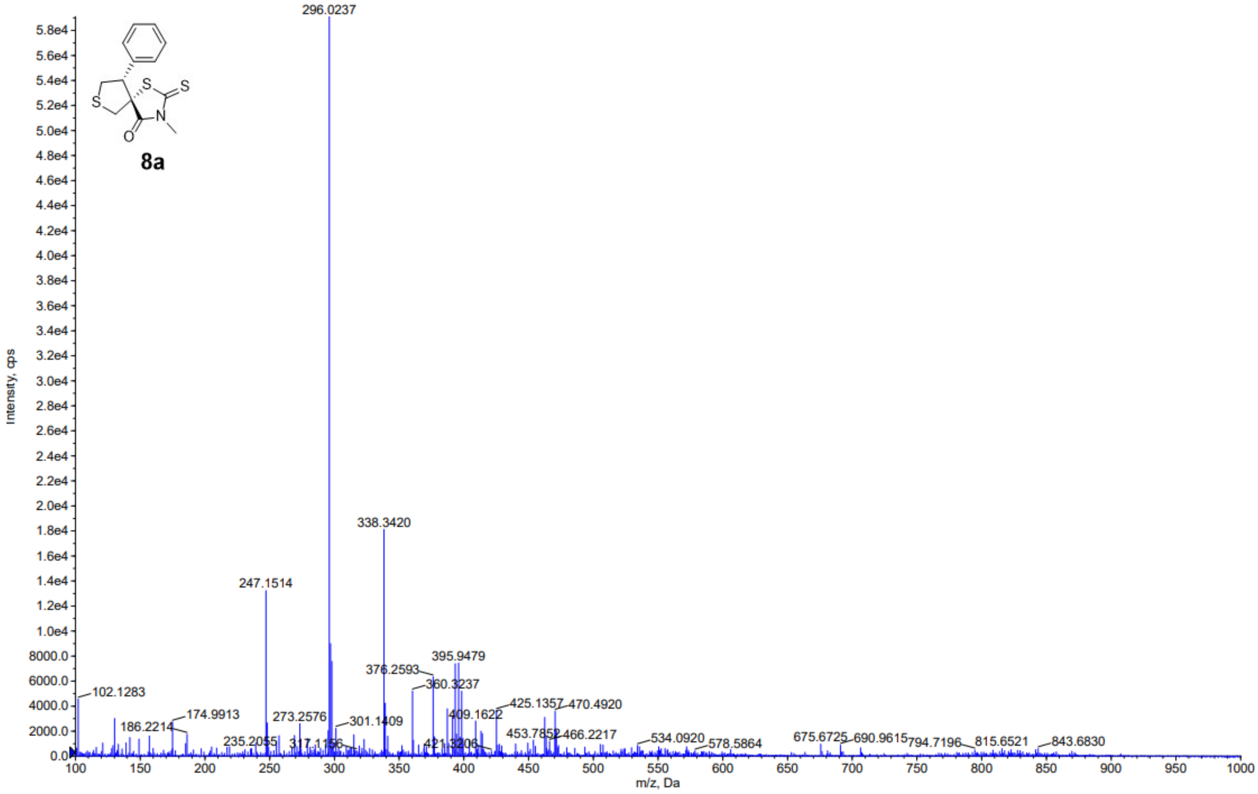
8a
296.0237
338.3420
247.1514
395.9479
376.2593
360.3237
102.1283
174.9913
186.2214
273.2576
301.1409
409.1622
425.1357
470.4920
235.2055
317.1156
421.3206
453.7852
466.2217
534.0920
578.5864
675.6725
690.9615
794.7196
815.6521
843.6830
Intensity, cps
m/z, Da

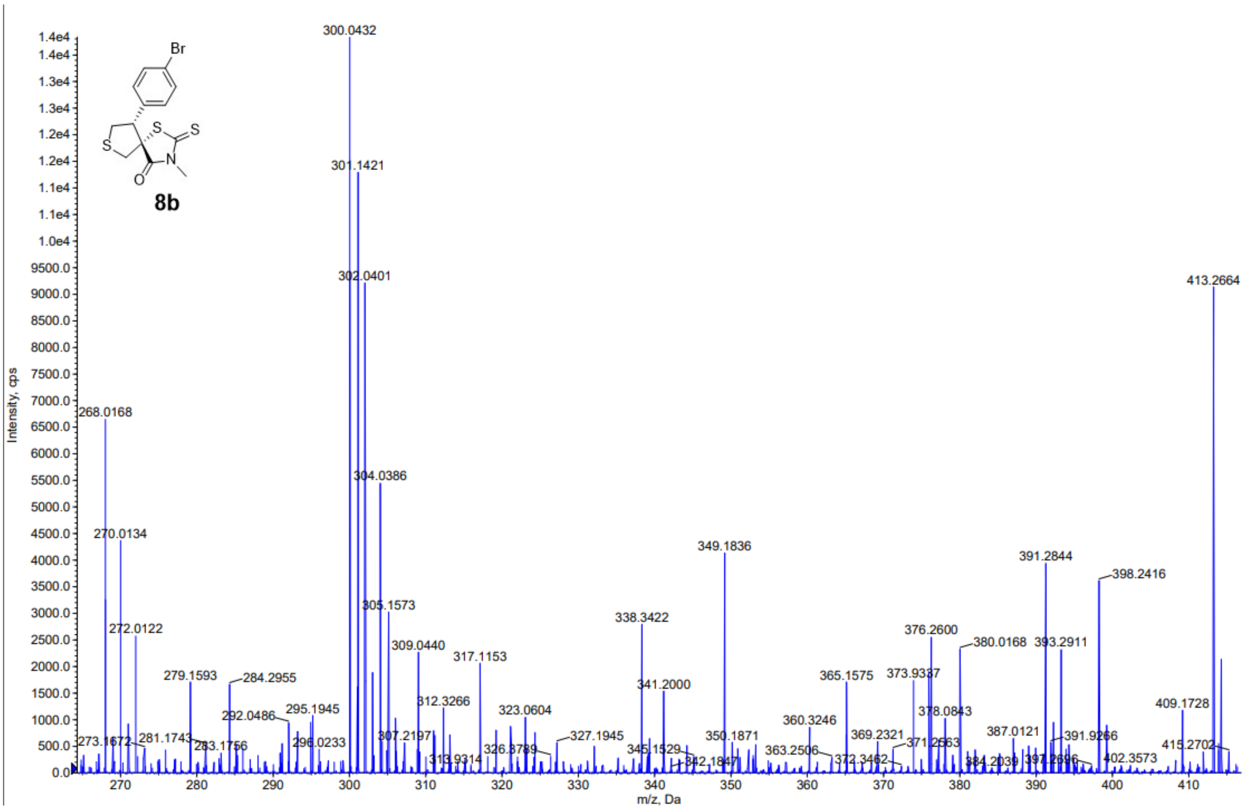
8b
Br
Intensity, cps
m/z, Da
300.0432
301.1421
302.0401
304.0386
413.2664
268.0168
270.0134
349.1836
391.2844
398.2416

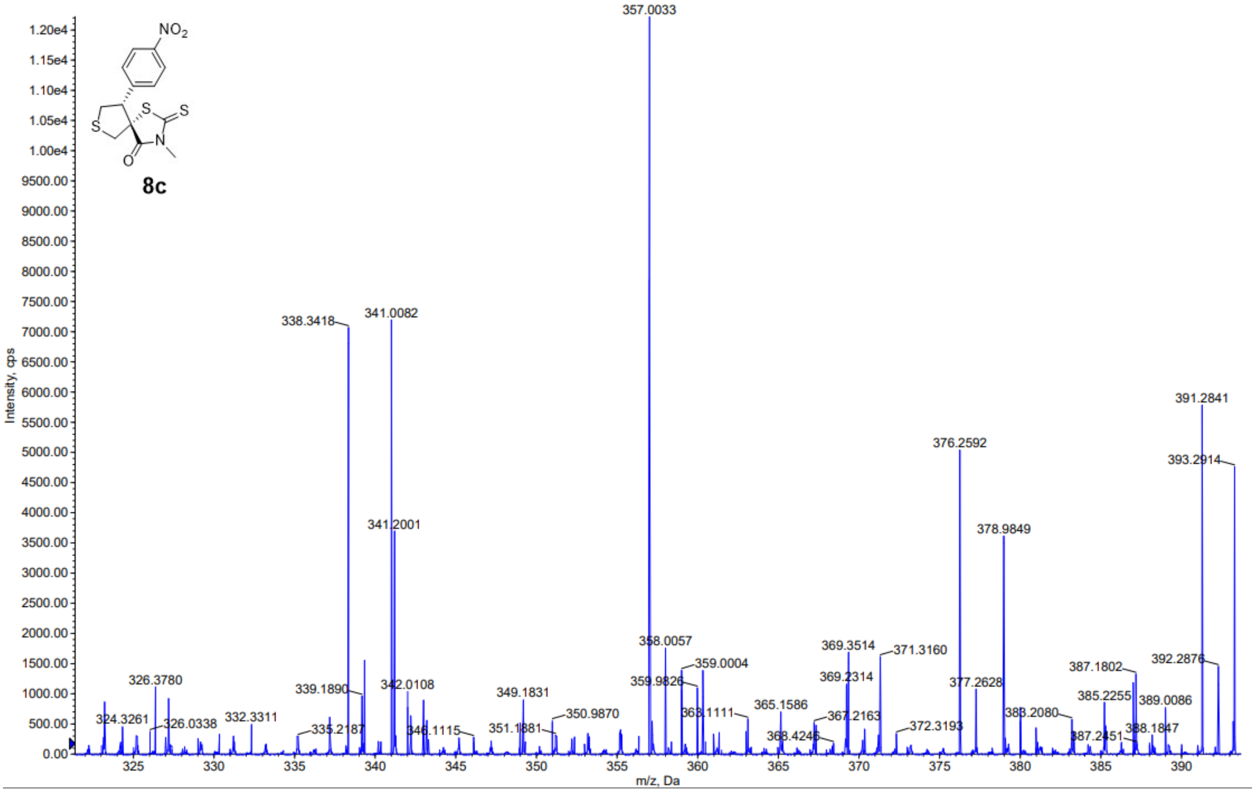
8c
NO2
Intensity, cps
m/z, Da
357.0033
338.3418
341.0082
341.2001
391.2841
393.2914
376.2592
378.9849

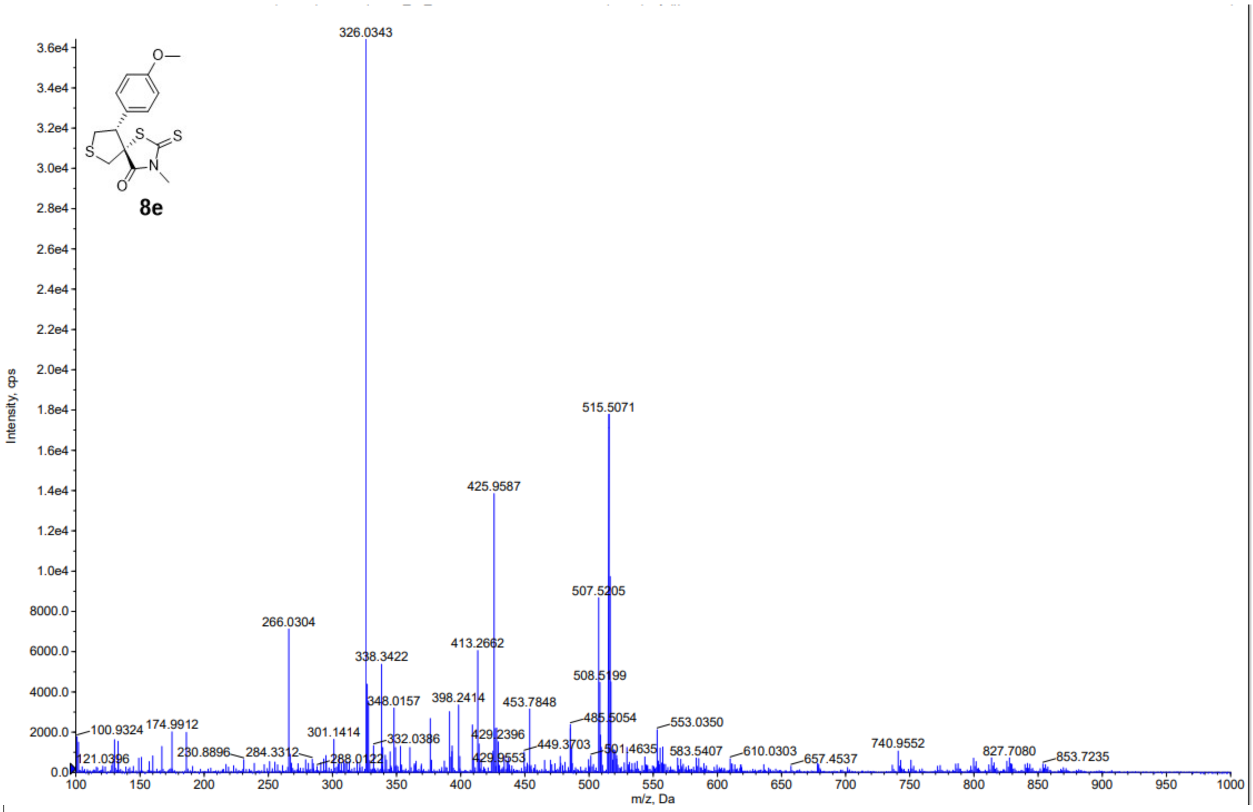
8e
Intensity, cps
m/z, Da
326.0343
515.5071
425.9587
507.5205
266.0304
413.2662
338.3422
508.5199
348.0157
398.2414
453.7848
485.5054
553.0350
100.9324
174.9912
301.1414
740.9552
827.7080
853.7235

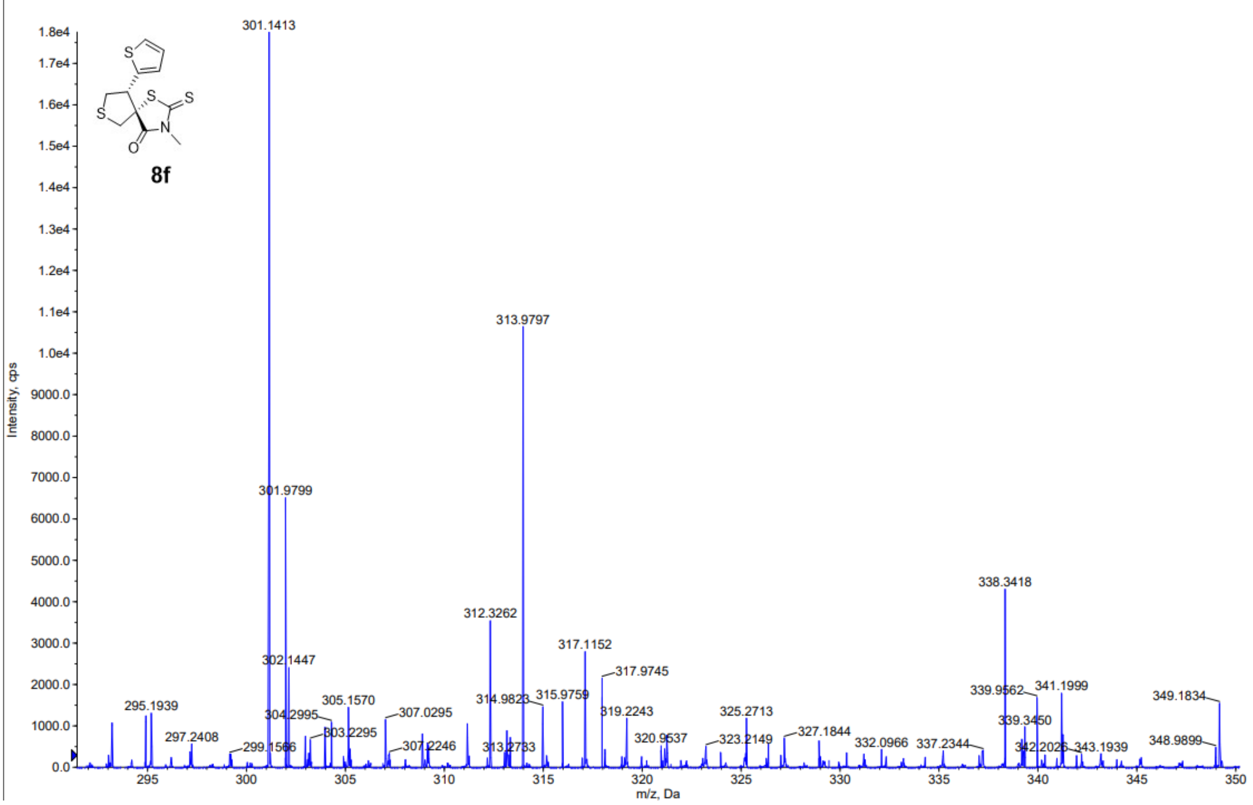
8f
Intensity, cps
m/z, Da
301.1413
313.9797
301.9799
338.3418
312.3262
317.1152
302.1447
317.9745
341.1999
339.9562
349.1834
295.1939
305.1570
307.0295

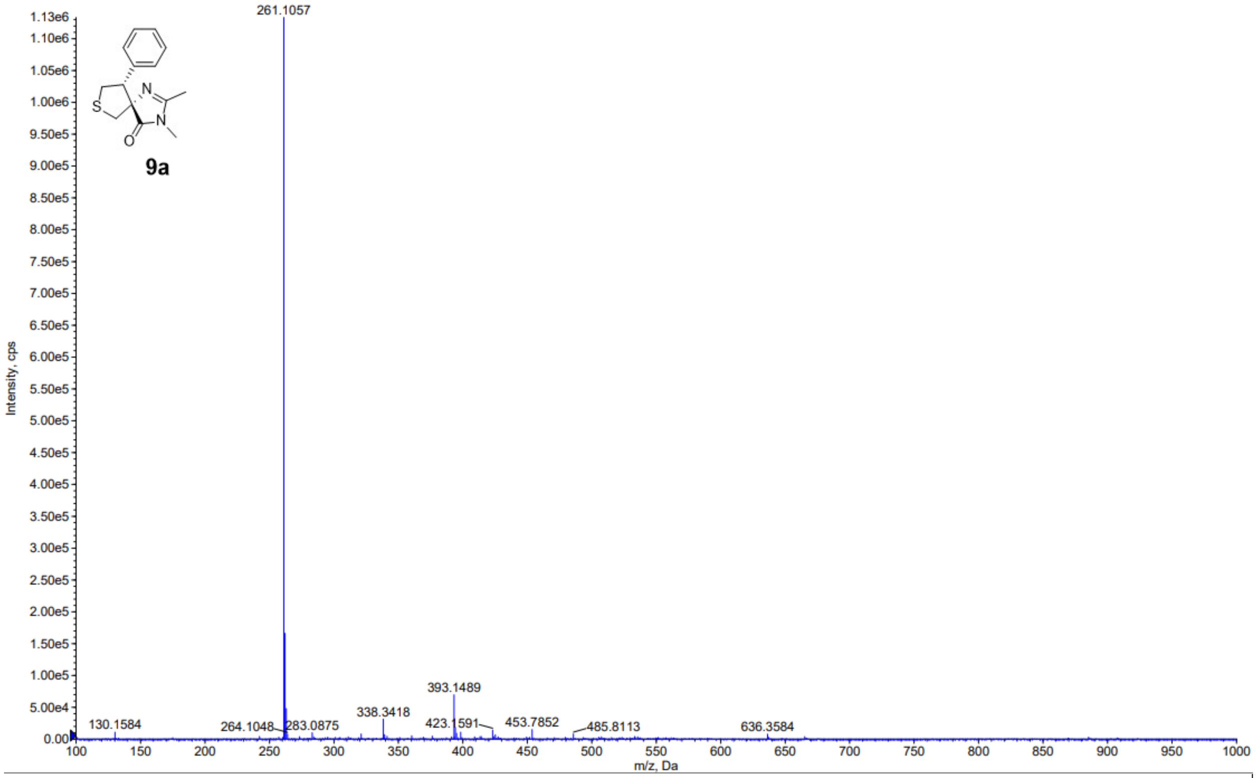
9a
261.1057
393.1489
338.3418
130.1584
264.1048
283.0875
423.1591
453.7852
485.8113
636.3584
Intensity, cps
m/z, Da

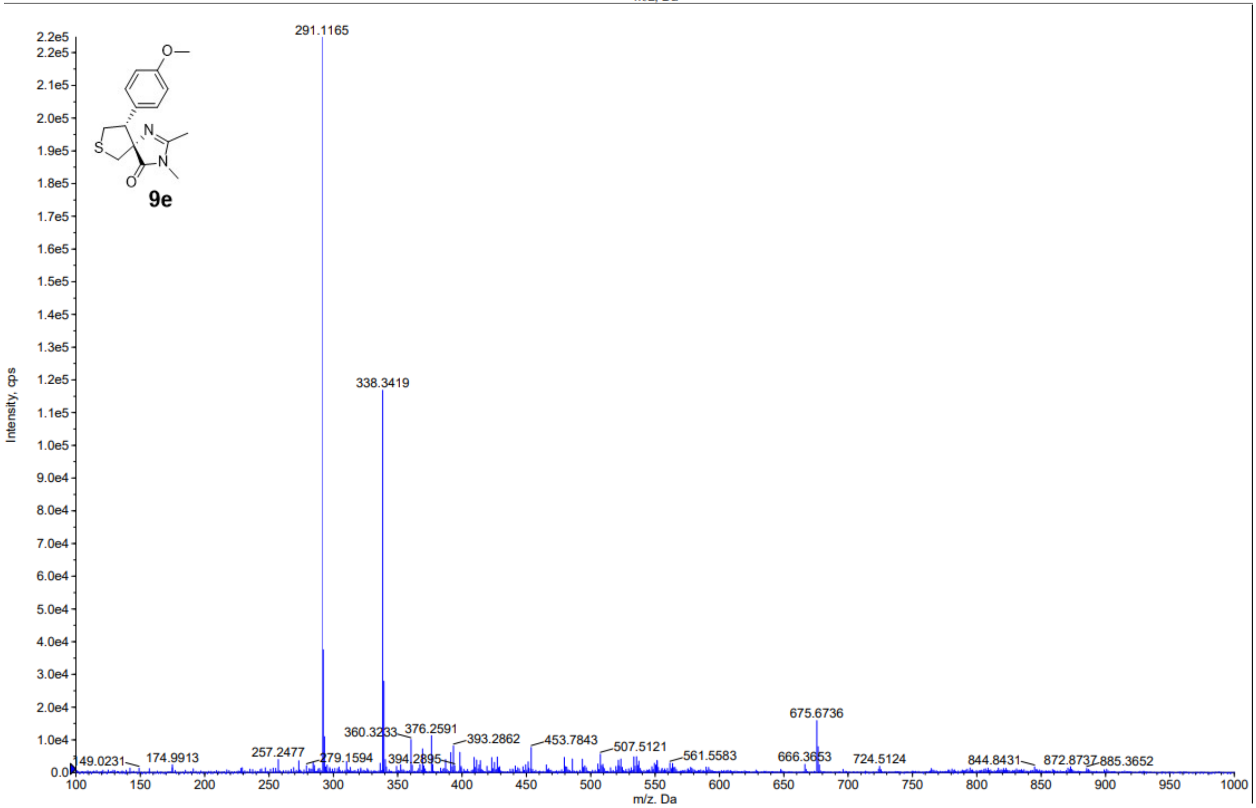
9e
291.1165
338.3419
675.6736
149.0231
174.9913
257.2477
279.1594
360.3233
376.2591
393.2862
394.2895
453.7843
507.5121
561.5583
666.3653
724.5124
844.8431
872.8737
885.3652
Intensity, cps
m/z, Da
